# Multicomponent, Functionalized HKUST-1 Analogues Assembled via Reticulation of Prefabricated Metal–Organic Polyhedral Cavities

**DOI:** 10.1021/jacs.2c06131

**Published:** 2022-08-16

**Authors:** Akim Khobotov-Bakishev, Cornelia von Baeckmann, Borja Ortín-Rubio, Laura Hernández-López, Alba Cortés-Martínez, Jordi Martínez-Esaín, Felipe Gándara, Judith Juanhuix, Ana E. Platero-Prats, Jordi Faraudo, Arnau Carné-Sánchez, Daniel Maspoch

**Affiliations:** † Catalan Institute of Nanoscience and Nanotechnology (ICN2), CSIC, and Barcelona Institute of Science and Technology, Campus UAB, 08193 Bellaterra, Barcelona, Spain; ∥ Departament de Química, Facultat de Ciències, Universitat Autònoma de Barcelona, 08193 Bellaterra, Spain; ‡ Consejo Superior de Investigaciones Científicas (CSIC), Materials Science Institute of Madrid (ICMM), Calle Sor Juana Inés de la Cruz, 3, 28049 Madrid, Spain; # ALBA Synchrotron, Carrer de la Llum, 2, 26, 08290 Cerdanyola del Vallès, Barcelona, Spain; § Departamento de Química Inorgánica, Facultad de Ciencias, Universidad Autónoma de Madrid, 28049 Madrid, Spain; ¶ Condensed Matter Physics Center (IFIMAC), Universidad Autónoma de Madrid, 28049 Madrid, Spain; ⊥ Institut de Ciència de Materials de Barcelona (ICMAB-CSIC), 08193 Bellaterra, Spain; ⊗ ICREA, Pg. Lluís Companys 23, 08010 Barcelona, Spain

## Abstract

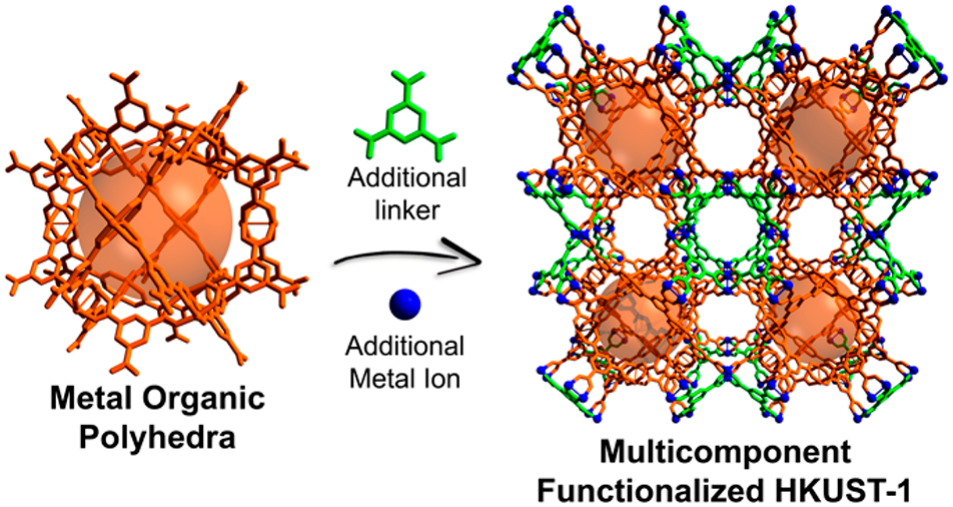

Metal–organic frameworks (MOFs) assembled from multiple building blocks exhibit greater chemical complexity and superior functionality in practical applications. Herein, we report a new approach based on using prefabricated cavities to design isoreticular multicomponent MOFs from a known parent MOF. We demonstrate this concept with the formation of multicomponent HKUST-1 analogues, using a prefabricated cavity that comprises a cuboctahedral Rh(II) metal–organic polyhedron functionalized with 24 carboxylic acid groups. The cavities are reticulated in three dimensions via Cu(II)-paddlewheel clusters and (functionalized) 1,3,5-benzenetricarboxylate linkers to form three- and four-component HKUST-1 analogues.

## Introduction

The combination of multiple organic linkers and metal ions into multicomponent or multivariate metal–organic frameworks (MOFs) is a fruitful strategy to achieve greater chemical complexity in MOFs, expand the catalogue of MOFs accessible by synthesis, and optimize the use of MOFs for applications such as gas storage,^1^ water harvesting,^2^ and catalysis.^3^ In these MOFs, complexity derives from the random or periodic arrangement of multiple organic and metallic functionalities into the same structure.^4^ To date, strategies to design multicomponent or multivariate MOFs include bottom-up synthesis by using any of the following: distinct linkers that have identical backbones but differ in their respective side groups or isostructural clusters comprising different metal ions, to produce multivariate MOFs;^5,6^ or structurally different linkers or metal clusters, to generate ordered multicomponent MOFs.^7−10^ Alternatively, the complexity of parent MOF structures can be augmented through post-synthetic modification via covalent and coordination chemistry,^11,12^ ligand installation,^13^ and linker- or metal-exchange.^14^

Herein we propose a new approach to isoreticular, multicomponent MOFs by starting with a known MOF. Reticular chemistry enables the rational synthesis of MOFs through the connection of basic molecular building blocks (MBBs).^15,16^ For example, the archetypical HKUST-1 is typically described as a 3,4-connected (3,4-c) network with an underlying **tbo** topology that is assembled from two MBBs: the 4-c Cu(II)-paddlewheel cluster and the 3-c 1,3,5-benzenetricarboxylate (btc) linker.^17^ However, on a conceptual level, MOFs can also be seen as the product of connecting higher order structures^18,19^ such as different cavities or metal–organic polyhedral (MOP)^20^ units, whether directly or through additional, small MBBs.^21,22^ Herein, we propose using MOPs as prefabricated cavities from which a parent MOF structure can be replicated, whereby its composition is changed.

Our strategy begins with a de-reticulation exercise in which a repetitive cavity of the parent MOF is identified. Following the example of HKUST-1, this enabled us to identify a repetitive cavity that defines a 24-c cuboctahedral MOP. Thus, we reasoned that the formation of the HKUST-1 structure would require the connection of these MOPs through the original 4-c Cu(II)-paddlewheel cluster and the 3-c btc. This leads to a change in the structural description of HKUST-1, from a binary 3,4-c structure that comprises one inorganic (Cu(II)-paddlewheel cluster) and one organic (btc) MBB to a tertiary 3,4,24-c structure that comprises these two MBBs and the 24-c MOP. We anticipated that this would enable use of three components in the synthesis of HKUST-1 that, if distinct, would occupy specific positions in the replicated structure, thereby generating ordered multicomponent MOFs isoreticular to HKUST-1. The basis of our approach is also supported by the supermolecular building block approach described by Eddaoudi et al., in which an *in situ*-synthesized^23,24^ or pre-assembled^25^ MOP is used as a highly connected node encoded with specific geometric and connectivity information to reduce the degrees of freedom of the network’s constituents and direct their assembly toward a target highly connected structure. However, herein, we use MOPs in a different way. In the prefabricated directed synthesis, the MOP does not behave as an *in situ*-formed node in a network, but rather as a preformed tiling of the targeted network, one which dictates the arrangement of the metallic and organic MBBs around it to ultimately generate a structure that is not necessarily described as highly connected (in this case, HKUST-1). Thus, the work that we present here expands the utility of MOPs in MOF chemistry, thereby providing a new route to complex multicomponent networks.

## Results and Discussion

### Prefabricated Cavity-Directed Synthesis of Multimetallic HKUST-1

**Figure 1:**
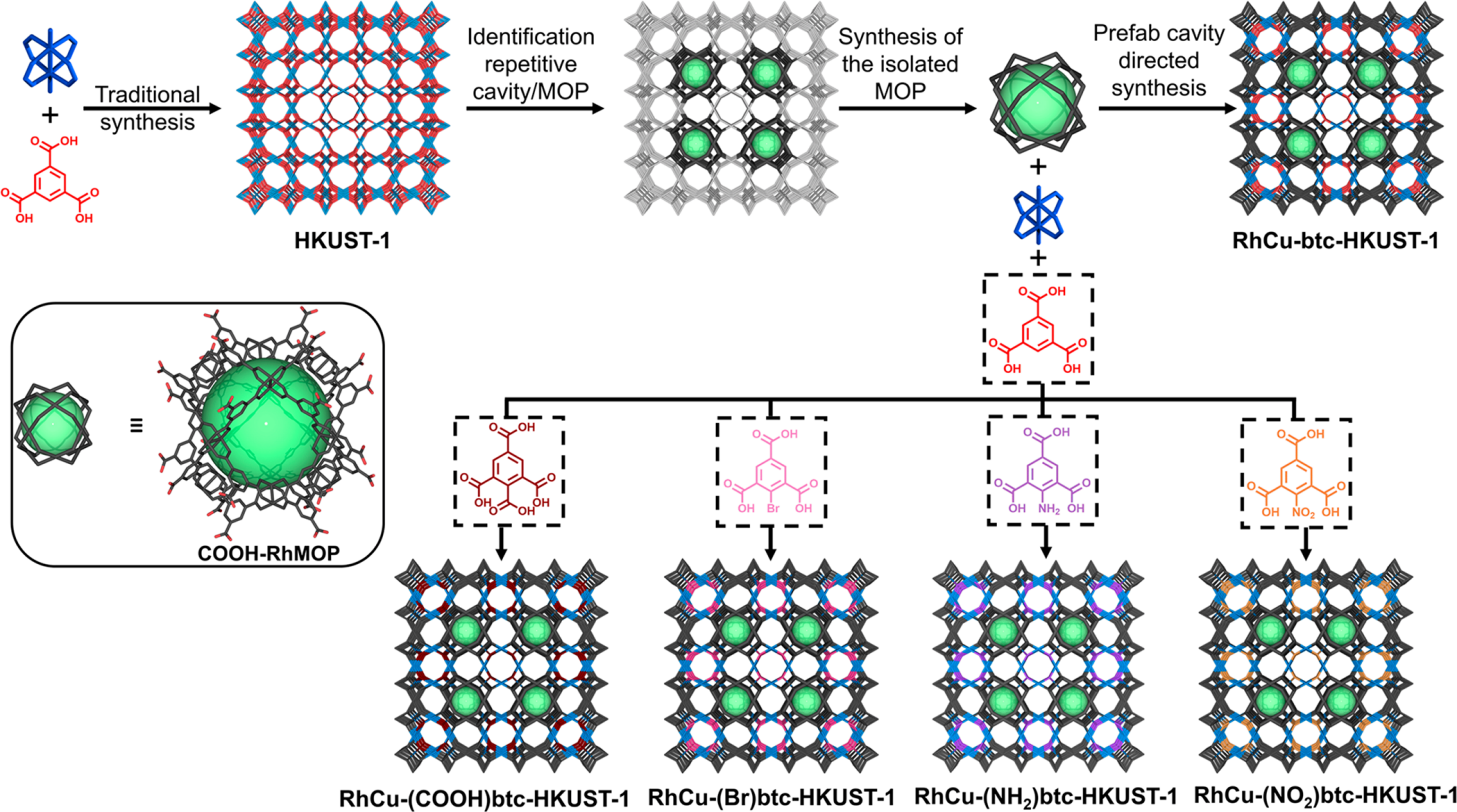
Schematic of the synthesis of three- and four-component HKUST-1 analogues using our design approach to multicomponent MOFs, based on the identification and exploitation of prefabricated cavities in the corresponding parent MOF.

We first applied our prefabricated cavity approach to HKUST-1 (Figure 1), choosing our previously reported Rh(II) cuboctahedral MOP functionalized with 24 carboxylic acid groups (hereafter named COOH-RhMOP) as the prefabricated cavity.^25,26^ We selected COOH-RhMOP because of its high chemical stability^27^ and its structural difference relative to its Cu(II) analogue, which would lead to pure HKUST-1. Using this MOP as a prefabricated cavity enabled us to replicate the structure of HKUST-1, thereby forming the isoreticular three-component RhCu-btc-HKUST-1 comprising COOH-RhMOPs, Cu(II)-paddlewheel clusters, and btc.

**Figure 2:**
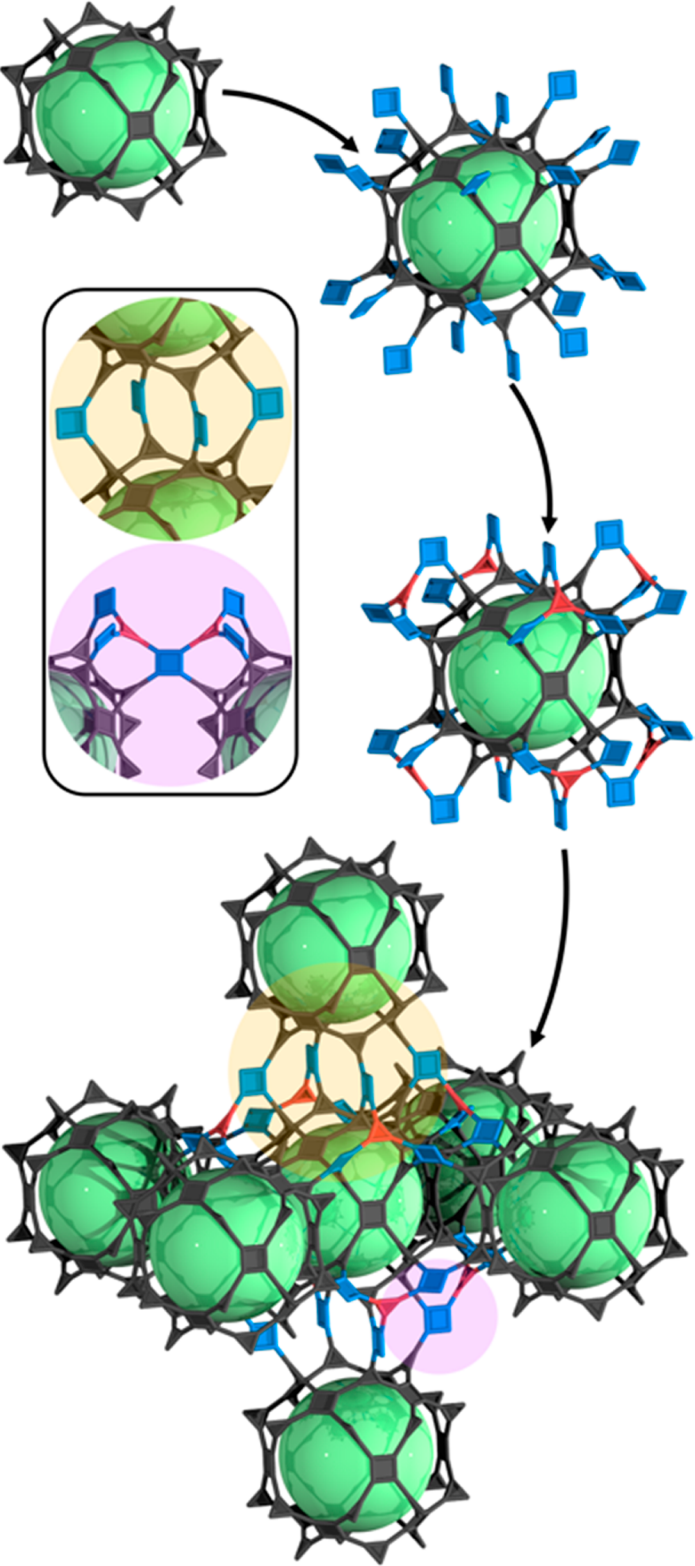
Schematic of the connectivity of COOH-RhMOPs (dark gray cages), Cu(II)-paddlewheel clusters (blue squares), and btc linkers (red triangles) to form RhCu-btc-HKUST-1. The inset details the connectivity between two COOH-RhMOPs.

Using COOH-RhMOP as a prefabricated cavity in the synthesis of RhCu-btc-HKUST-1 requires the stoichiometric addition of the three MBBs: COOH-RhMOP, the Cu(II)-paddlewheel cluster, and btc. To determine this stoichiometry, we studied the connectivity of the three MBBs in the targeted structure (Figure 2). To mimic this structure, each COOH-RhMOP must be connected to six neighboring COOH-RhMOPs through 24 Cu(II)-paddlewheel clusters. In this connectivity, each COOH-RhMOP is bridged to a neighboring COOH-RhMOP via four Cu(II)-paddlewheel clusters (Figure 2, yellow inset). Each Cu(II)-paddlewheel cluster must then be connected to four other Cu(II) clusters, via coordination of two btc linkers to their two remaining adjacent positions (Figure 2, violet inset). Overall, this connectivity defines a Cu(II)-cluster/btc/COOH-RhMOP ratio of 12:8:1. This connectivity also defines the relative position of each metal ion within the HKUST-1 network. Thus, RhCu-btc-HKUST-1 would present two types of cuboctahedral cavities in its structure: the Rh(II)-based cavity that derives from the prefabricated cavity, and a mixed-metal cavity containing Cu(II) and Rh(II) ions generated upon the self-assembly reaction. These cavities alternate throughout the structure (Figure S1). This degree of control over the relative position of cavities that contain different functionalities within porous frameworks has only been demonstrated for mixed-cage porous solids, in which different MOPs are co-precipitated.^28−30^

**Figure 3:**
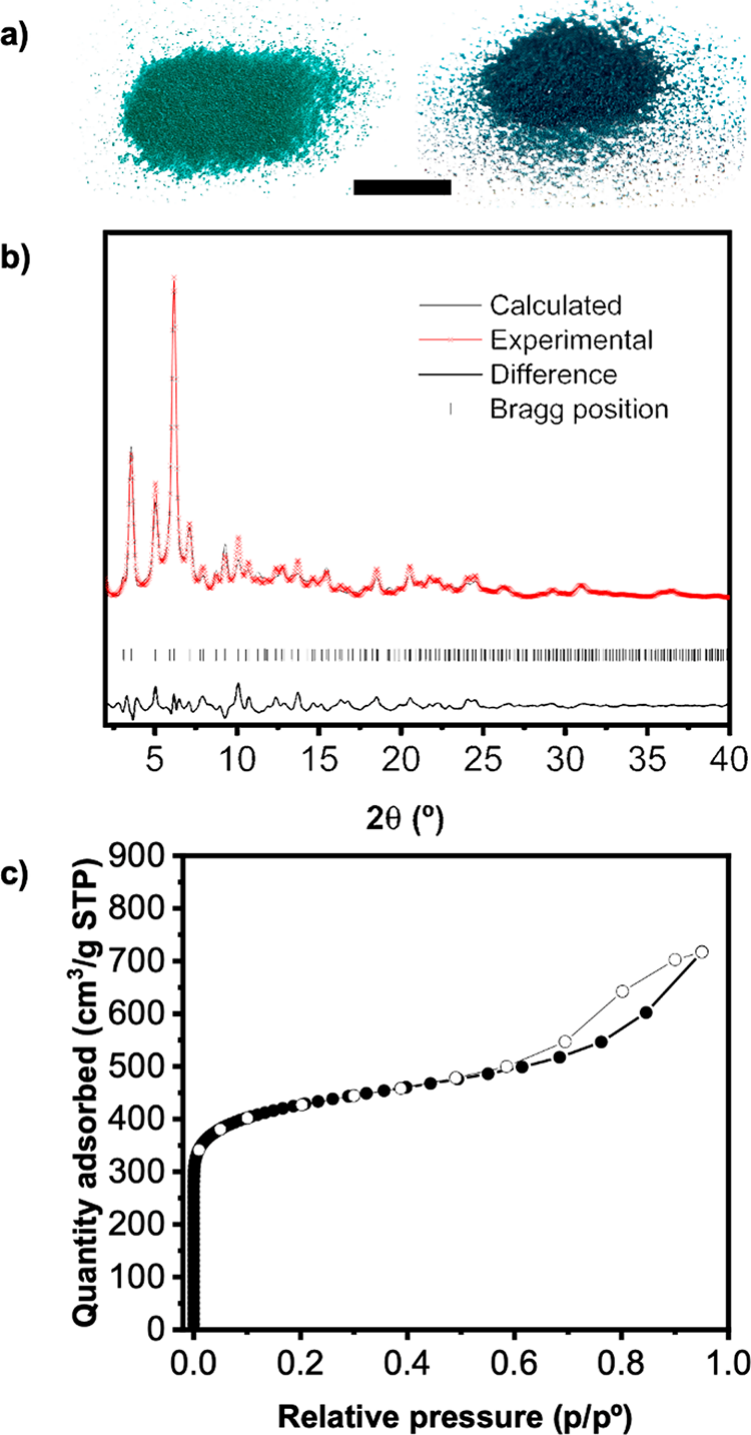
(a) Photographs of as-made (left) and activated (right) RhCu-btc-HKUST-1 powder. Scale bar: 1 cm. (b) Rietveld analysis of RhCu-btc-HKUST-1. (c) N_2_-sorption isotherms for RhCu-btc-HKUST-1.

We began the synthesis of RhCu-btc-HKUST-1, whose formula is [COOH-RhMOP(Cu)_24_(btc)_8_], by heating a mixture of COOH-RhMOP with 24 mol equiv of Cu(NO_3_)_2_·3H_2_O and 8 mol equiv of btc in *N*,*N*-dimethylformamide (DMF) at 85 °C for 1 day. The solvothermal reaction yielded a colloidal green dispersion. A green crystalline solid (yield: 78%; Figure 3a) was then isolated through centrifugation, washed with DMF, methanol, water, and acetone, and then dried at room temperature. Field emission scanning electron microscopy (FESEM) analysis of the green solid revealed the formation of a uniform sample comprising particles having an average size of 22 ± 3 nm (Figure S2). Energy-dispersive X-ray spectroscopy performed on these single particles using high-resolution transmission electron microscopy corroborated the presence of both Rh and Cu in each tested particle (Figure S3). Moreover, the oxidation states of both Rh and Cu were found to be +2 through X-ray photoelectron spectroscopy (Figure S4). Inductively coupled plasma-mass spectrometry (ICP-MS) measurements performed in acid-digested samples revealed that the Cu/Rh ratio was 1.02 ± 0.02, in agreement with the expected ratio in RhCu-btc-HKUST-1.

Synchrotron powder X-ray diffraction (PXRD) data collected on RhCu-btc-HKUST-1 revealed a pattern nearly coincidental with that of the parent Cu(II)-based HKUST-1 (Figure S5), with only slight shifts in the position of the peaks attributable to small differences in lattice parameters. Starting with the reported HKUST-1 atomic positions in the cubic *Fmm*3̅*m* space group, a satisfactory Rietveld refinement was reached (Rp = 3.32%, Rwp = 4.68%), corresponding to a structure in which Rh(II) and Cu(II) atoms each occupy 50% of the crystallographic metal site in the paddlewheel clusters (Figure 3b and Table S1). This refinement demonstrated that RhCu-btc-HKUST-1 is isoreticular to HKUST-1, having the same network type. While the metal atoms in RhCu-btc-HKUST-1 are located at topologically and symmetrically equivalent positions, their framework distribution in the MBBs is directed by using COOH-RhMOP. In addition, pair distribution function analyses of synchrotron X-ray scattering data showed the uniquely occurrence of Cu···Cu and Rh···Rh distances, thereby demonstrating the lack of hetero-bimetallic paddlewheel clusters in RhCu-btc-HKUST-1 (Figures S6 and S7).

To further confirm the MOP-guided assembly of RhCu-btc-HKUST-1, we ran a series of control experiments (see the Supporting Information (SI)). Initially, we corroborated the stability of COOH-RhMOP under the reaction conditions (DMF, 85 °C, 1 day) by ^1^H NMR, UV–vis, and mass spectrometry (Figures S9–S11). Then, we ran three blank reactions under the above conditions but lacking one of the three MBBs. As expected, we did not observe the formation of RhCu-btc-HKUST-1 in any of those reactions. Specifically, the reaction of Cu(II) and btc yielded microcrystals of the expected parent, Cu(II)-HKUST-1. The reaction of COOH-RhMOP with btc produced a clear green solution without any precipitate. This result further confirms that there is no leaching of Rh(II) ions from COOH-RhMOP; as these eventual leached Rh(II) ions would react with btc to yield an extended coordination polymer.^26^ Finally, the reaction of COOH-RhMOP with Cu(II) yielded an amorphous coordination polymer. Additionally, we reacted preformed Cu(II)-HKUST-1 crystals with COOH-RhMOP in a mixture containing the same molar ratio of Cu(II)-cluster/btc/COOH-RhMOP as that (12:8:1) used for the synthesis of RhCu-btc-HKUST-1. Under these conditions, we did not observe the formation of RhCu-btc-HKUST-1 crystals (Figure S12). This experiment demonstrates that the reaction mechanism cannot proceed through an initial formation of Cu(II)-HKUST-1 crystals that evolve through solubilization–recrystallization toward the formation of RhCu-btc-HKUST-1. We reasoned that, conversely, the most plausible scenario is that the presence of the COOH-RhMOP rapidly nucleates the formation of RhCu-btc-HKUST-1, thereby suppressing the formation of Cu(II)-HKUST-1.

We confirmed the presence of COOH-RhMOP cavities and btc linkers within the structure of RhCu-btc-HKUST-1 through solid-state cross-polarized/magic angle spinning (CP/MAS) ^13^C NMR (Figure S13). To quantify the molar ratio between the prefab cavities and the added btc linkers, we developed a methodology to revert the assembly process into its initial components, which we identified and then quantified (Figures S14–S17). This was based on the high chemical stability of COOH-RhMOP. Upon exposing a DMF dispersion of RhCu-btc-HKUST-1 crystals to acidic conditions (see SI), we found that they become fully redissolved. ^1^H NMR (DMF-*d*_7_) of the resulting solution revealed a btc/COOH-RhMOP ratio of 8:1, in agreement with the ratio expected in RhCu-btc-HKUST-1 (Figure S16). We were able to quantify the amount of liberated COOH-RhMOP by UV–vis spectroscopy. From this experiment, we calculated a concentration of 93.3 μmol COOH-RhMOP/g of RhCu-btc-HKUST-1, which is very close to the theoretical value (94.2) (Figure S17 and Table S2). Altogether, our results confirmed the formation of RhCu-btc-HKUST-1 without significant defects and that COOH-RhMOP remains intact during its synthesis.

Next, we performed N_2_-sorption measurements on activated RhCu-btc-HKUST-1 at 77 K, finding that it is microporous to N_2_, with a BET surface area (*S*_BET_) of 1606 m^2^/g (Figure 3c, Figure S18). Furthermore, pore-size distribution analysis revealed the presence of the three characteristic cavities of the HKUST-1 structure together with some mesoporosity, which we ascribed to the interparticle voids (Figure S20). This extrinsic porosity is also responsible for the increased uptake at high pressure (*P*/*P*_0_ ≈ 0.6) and the observed hysteresis loop, as previously observed for other nanoscopic MOFs.^31^ PXRD diffractogram recorded after these sorption studies confirmed that the RhCu-btc-HKUST-1 had retained its crystallinity (Figure S21).

### Hydrolytic Stability of RhCu-btc-HKUST-1

**Figure 4:**
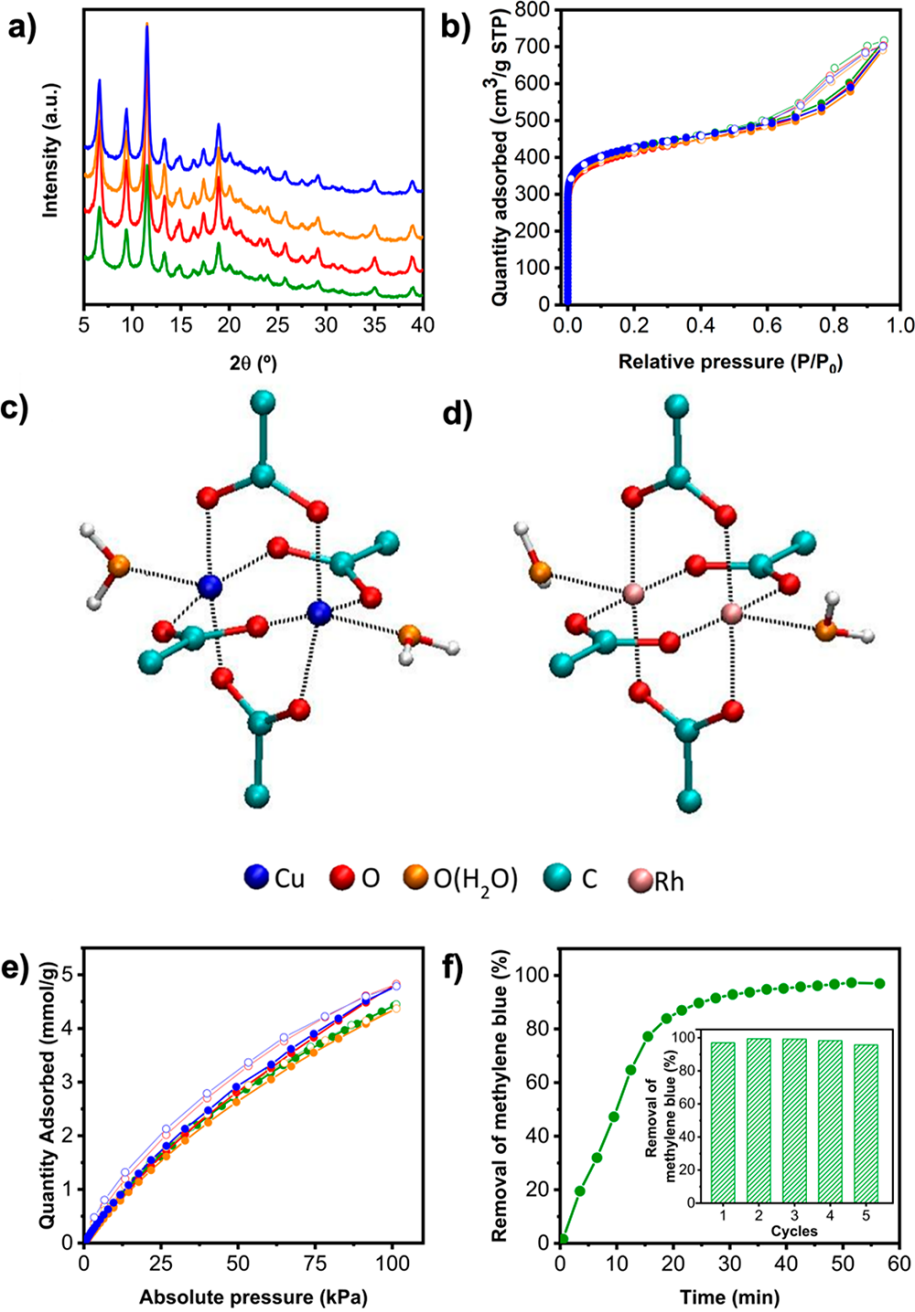
(a) PXRD diffractogram and (b) N_2_-sorption isotherms for RhCu-btc-HKUST-1 initially (green) and after incubation in water for 3 (red), 14 (orange), and 31 days (blue). Snapshots (orthographic views) of the optimized DFT structures of (c) Cu(II)- and (d) Rh(II)-paddlewheel clusters in water. (e) CO_2_-sorption isotherms at 298 K for RhCu-btc-HKUST-1 initially (green) and after incubation in water for 3 days (red), 14 days (orange), and 31 days (blue). (f) Removal of MB in water by RhCu-btc-HKUST-1 as a function of time. Inset: five consecutive MB-removal/regeneration cycles using RhCu-btc-HKUST-1.

We reasoned that the presence of the water-stable COOH-RhMOP cavity within RhCu-btc-HKUST-1 could confer the overall structure with greater hydrolytic stability relative to the parent Cu(II)-HKUST-1. To test this hypothesis, we incubated RhCu-btc-HKUST-1 and the parent Cu(II)-HKUST-1 in liquid water at room temperature from 1 to 31 days. The water-incubated samples were then characterized through FESEM, PXRD, and N_2_ sorption. To our surprise, RhCu-btc-HKUST-1 had retained its morphology, crystallinity, composition, and porosity, even after 1 month of incubation in liquid water (Figure 4a,b, Figures S23 and S30–S35). Conversely, upon exposure to water, the parent Cu(II)-HKUST-1 had undergone the well-reported phase change, with a corresponding loss of porosity from 1888 to 502 m^2^/g within the first day (Figures S24–S29).^32^

The hydrolytic stability that we observed for RhCu-btc-HKUST-1 implies that not only the COOH-RhMOP cavities, but also the Cu(II)-carboxylate bonds that link them, withstand the incubation in water. Thus, in an effort to rationalize the higher hydrolytic stability of RhCu-btc-HKUST-1, we performed electronic structure calculations in water of both Cu(II)- and Rh(II)-paddlewheel clusters (Figure 4c,d). For this, we employed Gaussian 16^33^ at the M06-L/SDD level of theory^34^ in the presence of implicit water solvent modeled with the IEFPCM formalism, as described in the SI.^35^ Our implicit solvent calculations showed that the Cu(II)-paddlewheel undergoes a significant torsion when exposed to water, whereas the Rh(II)-paddlewheel remains stable (Figures S36–S38). This torsion is enhanced after coordination of a water molecule to the axial position of Cu(II) (Figure 4c, Figures S39–S41), disrupting the original symmetrical bidentate binding with carboxylate ligands (Figure S42). We propose that Cu(II)-paddlewheels are hydrolyzed through this mechanical distortion as pivotal step, provoking the instability of the parent Cu(II)-HKUST-1. In the case of RhCu-btc-HKUST-1, each Cu(II)-paddlewheel is connected to two Rh(II)-paddlewheel clusters, which are not altered by water. The inertness of the Rh(II)-paddlewheel blocks the mechanical instability of the neighboring Cu(II)-paddlewheel, thereby inhibiting the hydrolysis process.

We envisioned that the stability of RhCu-btc-HKUST-1 could enable its use as an adsorbent in aqueous environments or after aqueous exposure. As a proof of concept, we evaluated the CO_2_ adsorption capacity of RhCu-btc-HKUST-1 after being incubated in water for up to 31 days. As observed in Figure 4e, CO_2_ uptake capacity did not decrease after the incubation. Additionally, we tested the adsorption capabilities of RhCu-btc-HKUST-1 in liquid water. To this end, we incubated RhCu-btc-HKUST-1 in an aqueous solution of methylene blue (MB) at 20 ppm (pH = 7) and then monitored the decrease of MB over 1 h. We found that, after 51 min, 97% of the MB had been removed by the RhCu-btc-HKUST-1 (Figure 4f). Moreover, after the MB-adsorption, the RhCu-btc-HKUST-1 fully retained its crystallinity (Figure S44). This contrasts sharply to the case of its parent, Cu(II)-HKUST-1, which, in the same amount of time, could adsorb 62% of the MB, due to its degradation and amorphization in water (Figures S43 and S45). To explore the MB-removal performance of each analogue after reutilization, we tested them over five consecutive removal/regeneration cycles. The removal step was identical to the one followed above. The regeneration step entailed the recovery of the adsorbent through centrifugation, followed by successive washings with water and acetone. Finally, the adsorbent was activated at 85 °C under vacuum for 1 h. The results showed that uptakes of RhCu-btc-HKUST-1 were similar among the five cycles (Figure 4f, inset), meaning that it had remained stable and that the regeneration was sufficient to maintain its removal capacity. Contrariwise, under these conditions, the MB-removal capacity of Cu(II)-HKUST-1 dropped from 62% to ∼10% (from the second to third cycles), and then to ∼3–4% (for the fourth and fifth cycles) (Figure S43). Thus, the difference in MB-removal performance between RhCu-btc-HKUST-1 and Cu(II)-HKUST-1 only widened after reutilization, suggesting a new mechanism for stabilization of Cu(II) paddlewheel clusters based on the mechanical interlock between Rh(II) and Cu(II) paddlewheels, which results in water-resistant adsorbents.

### Reticulation of Varied Linkers into the HKUST-1 Structure via Prefabricated Cavity-Directed Synthesis

**Figure 5:**
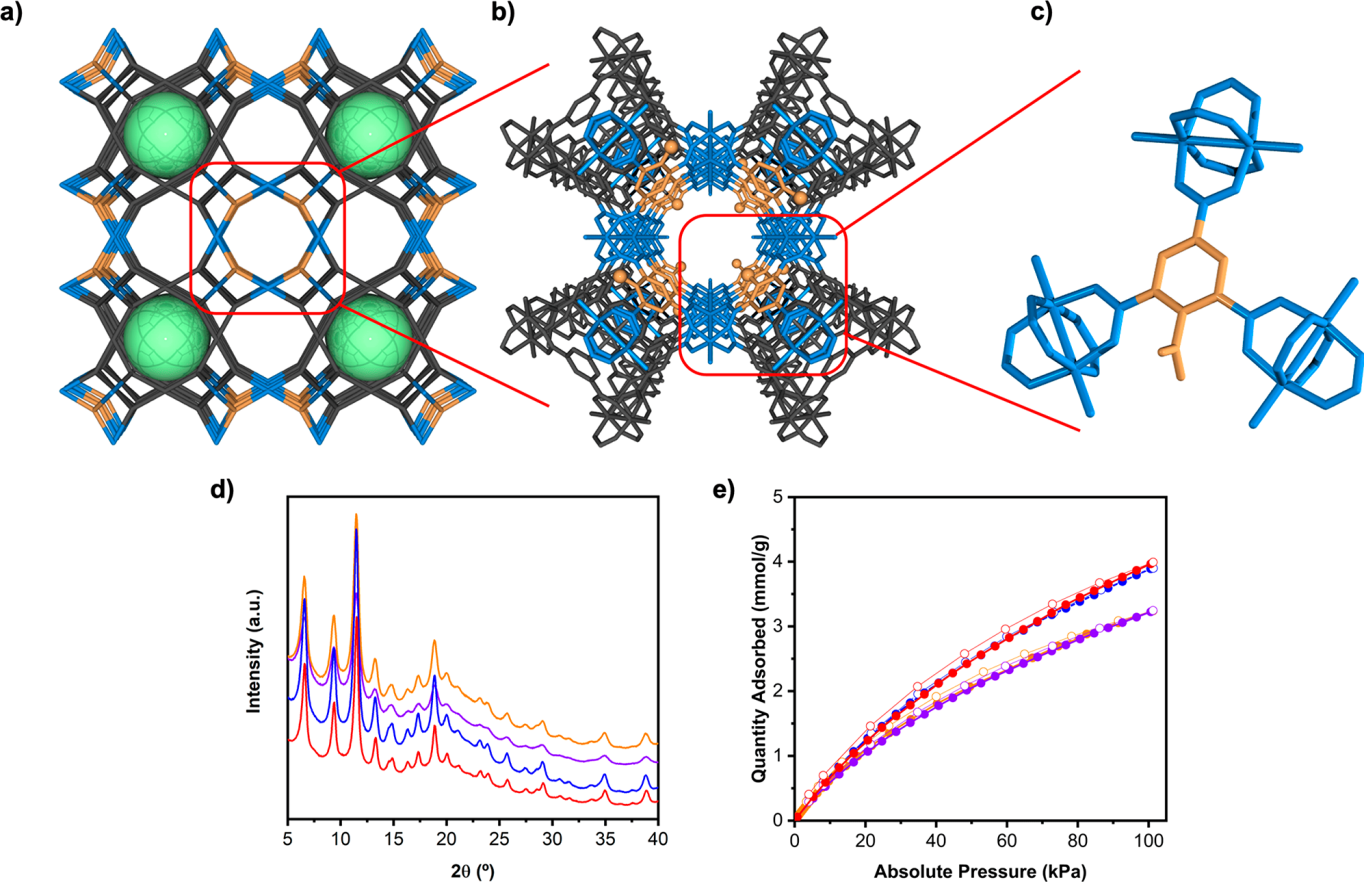
(a) Schematic of the four-component HKUST-1 analogues, showing the three types of channels that are generated when the COOH-RhMOP (structure depicted in gray and cavity depicted in green) is co-assembled with Cu(II) paddlewheels (blue) and (functionalized) btc linkers (orange). (b) Highlight of the chemical structure of the 1D channel decorated exclusively with functionalized btc linkers. (c) Highlight of the coordination environment of the (COOH)btc linker within the channels of RhCu-(COOH)btc-HKUST-1. (d) PXRD diffractogram of RhCu-(NH_2_)btc-HKUST-1 (blue), RhCu-(NO_2_)btc-HKUST-1 (orange), RhCu-(Br)btc-HKUST-1 (purple), and RhCu-(COOH)btc-HKUST-1 (red). (e) CO_2_-adsorption isotherms at 298 K for RhCu-(NH_2_)btc-HKUST-1 (blue), RhCu-(NO_2_)btc-HKUST-1 (orange), RhCu-(Br)btc-HKUST-1 (purple), and RhCu-(COOH)btc-HKUST-1 (red).

We envisaged that our prefabricated cavity strategy would provide access to four-component HKUST-1 analogues, given the possibility to differentiate the btc linkers that form the COOH-RhMOP from those that bridge the Cu(II)-paddlewheel clusters, during the synthesis. However, we reasoned that such four-component analogues would require a functionalized btc linker, rather than the previously used btc linker. In this new configuration, the connectivity of the prefabricated COOH-RhMOP cavity dictates that the added functionalized btc linkers will be located on top of the triangular windows that connect three Cu(II) paddlewheels. These positions align into 1D channels, thus generating four-component HKUST-1 analogues in which alternating functionalized and non-functionalized 1D channels coexist (Figure 5a). This scenario differs from the outcome obtained when linkers with the same connectivity but different side functionalities are combined to generate isoreticular frameworks. In this latter case, one generally obtains structures in which the different linkers are randomly distributed or are organized into a non-atomically precise pattern.^36^

Based on the prefab cavity-induced desymmetrization of the organic linkers within the HKUST-1 network, we attempted the synthesis of four-component HKUST-1 analogues, using btc linkers having a pendant functional group [(Br)btc, (NO_2_)btc and (NH_2_)btc] at the second position of the phenylic ring as one of the reagents. Thus, we followed a synthetic strategy identical to the one that we had used for RhCu-btc-HKUST-1, except that we substituted the non-functionalized btc linker with either (Br)btc, (NO_2_)btc or (NH_2_)btc to generate RhCu-(Br)btc-HKUST-1, RhCu-(NO_2_)btc-HKUST-1, and RhCu-(NH_2_)btc-HKUST-1, respectively. The three reactions afforded green crystalline samples composed of particles with an average size of 24 ± 2 nm, for RhCu-(Br)btc-HKUST-1 (yield: 82%); 23 ± 2 nm, for RhCu-(NO_2_)btc-HKUST-1 (yield: 78%); and 21 ± 3 nm, for RhCu-(NH_2_)btc-HKUST-1 (yield: 74%) (Figures S46, S53, and S60).

Next, we characterized RhCu-(Br)btc-HKUST-1, RhCu-(NO_2_)btc-HKUST-1, and RhCu-(NH_2_)btc-HKUST-1 by PXRD, finding that their patterns matched the one that we had previously obtained for RhCu-btc-HKUST-1 (Figure 5d). ICP-MS on fully digested RhCu-(Br)btc-HKUST-1, RhCu-(NO_2_)btc-HKUST-1, and RhCu-(NH_2_)btc-HKUST-1 gave Cu/Rh molar ratios of 1.16 ± 0.01, 1.10 ± 0.01, and 1.09 ± 0.02, respectively. These values are in good agreement with the value (1) expected for their molecular formula. Moreover, ^1^H NMR signals of the digested materials showed btc/(Br)btc, btc/(NO_2_)btc, and btc/(NH_2_)btc ratios of 8:1, also in perfect agreement with the expected ratio according to their formula (Figures S48, S55, and S62).

We measured the porosity of RhCu-(Br)btc-HKUST-1, RhCu-(NO_2_)btc-HKUST-1, and RhCu-(NH_2_)btc-HKUST-1 in N_2_-sorption experiments, finding *S*_BET_ values of 1215, 1133, and 1212 m^2^/g, respectively (Figures S49, S56, and S63). In all cases, PXRD diagrams collected after the sorption studies also confirmed their stability (Figures S51, S58, and S65). All these *S*_BET_ values are lower than the *S*_BET_ value for RhCu-btc-HKUST-1. We ascribed their inferior porosity to steric hindrance of the side groups located within the pores—a feature common to many other MOFs, such as those of the UiO-66 family.^37^ Whereas N_2_-sorption isotherms at 77 K accounted for the steric hindrance of the functional groups introduced into these four-component HKUST-1 analogues, CO_2_-adsorption measured at 298 K highlighted their different affinities toward CO_2_. Thus, the presence of free amine groups in RhCu-(NH_2_)btc-HKUST-1 made it a better adsorbent for CO_2_ than its Br or NO_2_ analogues (Figure 5e).

### COOH-Functionalized HKUST-1 Analogue

Having observed the structure-directing properties of the COOH-RhMOP prefabricated cavity on the synthesis of multicomponent HKUST-1 analogues, we envisaged that it could be employed to reticulate tetracarboxylate linkers to functionalize the HKUST-1 architecture with free carboxylic acid groups. Thus, we employed 1,2,3,5-benzenetetracarboxylic acid (hereafter named (COOH)btc) as the organic MBB in the co-assembly of COOH-RhMOP with Cu(II) paddlewheels to yield RhCu-(COOH)btc-HKUST-1 (Figure 5b,c). The solvothermal reaction between COOH-RhMOP, Cu(NO_3_)_2_·3H_2_O, and (COOH)btc afforded a green crystalline sample made of particles of an average size of 20 ± 3 nm (yield: 65%) (Figure S67). PXRD analysis of RhCu-(COOH)btc-HKUST-1 revealed a pattern consistent with RhCu-btc-HKUST-1 (Figure 5d). The successful reticulation of (COOH)btc within the HKUST-1 network was confirmed by the ^1^H NMR spectrum of the acid-digested sample, which showed a btc/(COOH)btc ratio of 8:1 (Figure S69). The thermally activated sample retained its crystallinity (Figure S72), which enabled measurement of its gas sorption, for which an *S*_BET_ of 1380 m^2^/g (Figure S70) and a CO_2_ uptake (at 298 K and 1 bar) of 4.0 mmol/g were found (Figure 5e).

To further confirm that RhCu-(COOH)btc-HKUST-1 contained free carboxylic acid groups within its channels, we performed a series of spectroscopic characterizations. First, ICP measurements performed on the acid-digested sample revealed that the Cu:Rh molar ratio was 0.94. The fact that there is no excess of Cu(II) ions in the HKUST-1 structure suggests that only three of the four COOH groups of the (COOH)btc are coordinated to Cu(II) ions. Next, infrared spectroscopy performed on RhCu-(COOH)btc-HKUST-1 showed a clear vibration band at 1702 cm^–1^, which we ascribed to the stretching band of C=O, which indicates the presence of uncoordinated carboxylic acid groups (Figure S73). Altogether, our results illustrate that our prefabricated-cavity approach can be employed to restrict the connectivity of polycarboxylate linkers to introduce free carboxylic acid groups into multicomponent isoreticular structures. Accordingly, this approach enabled the synthesis of a COOH-functionalized HKUST-1 analogue without generation of any defective structures.^38,39^

Finally, as a proof-of-concept, we aimed to demonstrate that the carboxylic acid groups located within the RhCu-(COOH)btc-HKUST-1 structure are functional and accessible. To this end, we evaluated the behavior of RhCu-(COOH)btc-HKUST-1 as catalyst in a model acid-catalyzed reaction: the conversion of benzaldehyde dimethyl acetal to benzaldehyde.^40,41^ We observed that, under identical conditions, RhCu-(COOH)btc-HKUST-1 could convert up to 64% of benzaldehyde dimethyl acetal into benzaldehyde, whereas non-functionalized RhCu-btc-HKUST-1 only afforded 32% conversion (Figure S74). Considering that the acidic groups of RhCu-btc-HKUST-1 can only be located at its surface, we ascribed the superior conversion obtained with RhCu-(COOH)btc-HKUST-1 to the activity of the inner carboxylic acid groups, which would confirm their accessibility. Importantly, both RhCu-btc-HKUST-1 and RhCu-(COOH)btc-HKUST-1 fully retained their crystallinity after the acid catalysis (Figure S75).

## Conclusions

We have presented an alternative methodology to synthesize multicomponent MOFs, which is based on the co-assembly of prefabricated cavities (in the form of carboxylic acid-functionalized MOPs) and small MBBs. The methodology benefits from the structure-directing influence of the MOP to organize varied organic and metallic MBBs through the crystal lattice of the targeted MOF, thus providing a greater degree of control over the synthesis of atomically precise multicomponent MOFs.

## Supporting Information Available

Detailed syntheses, FESEM data and images, PXRD diffractogram, XPS, UV–vis, and NMR, as well as porosity measurements, including Figures S1–S75 and Tables S1–S3 (PDF)
