# Supplementary material for: Multicomponent, Functionalized HKUST-1 Analogues Assembled via Reticulation of Prefabricated Metal–Organic Polyhedral Cavities
Source: J Am Chem Soc. 2022 Aug 16;144(34):15745–53. doi: 10.1021/jacs.2c06131 (PMC9437915; doi:10.1021/jacs.2c06131)
Supplement: Supplementary file 1 — ja2c06131_si_001.pdf [file ja2c06131_si_001.pdf]

Supporting Information

Multicomponent, functionalized HKUST-1 analogues assembled via reticulation of prefabricated metal-organic polyhedral cavities

Akim Khobotov-Bakishev, Cornelia von Baeckmann, Borja Ortín-Rubio, Laura Hernández-López, Alba Cortés-Martínez, Jordi Martínez-Esaín, Felipe Gándara, Judith Juanhuix, Ana E. Platero-Prats, Jordi Faraudo, Arnau Carné-Sánchez*, and Daniel Maspoch*

Table of Contents

S1. Materials and methods.

S1.1. Materials and characterization.

S1.2. Experimental methods.

S1.2.1. Synthesis of COOH-RhMOP, (Br)btc, (NO_2)btc and (COOH)btc.

S1.2.2. Stability of COOH-RhMOP under solvothermal conditions.

S1.2.3. Synthesis of RhCu-btc-HKUST-1, RhCu-(Br)btc-HKUST-1, RhCu-(NO_2)btc-HKUST-1, RhCu-(NH_2)btc-HKUST-1 and RhCu-(COOH)btc-HKUST-1.

S.1.2.4. Blank reactions for RhCu-btc-HKUST-1.

S.1.2.5. Acidic disassembly of RhCu-btc-HKUST-1.

S.1.2.6. Acidic digestion of RhCu-(Br)btc-HKUST-1, RhCu-(NO_2)btc-HKUST-1, RhCu-(NH_2)btc-HKUST-1 and RhCu-(COOH)btc-HKUST-1.

S1.2.7. Study of the hydrolytic stability of RhCu-btc-HKUST-1 and Cu(II)-HKUST-1.

S1.2.8 Study of the methylene blue removal with RhCu-btc-HKUST-1 and Cu(II)-HKUST-1.

S1.2.9. Study of the catalytic activity of RhCu-btc-HKUST-1 and RhCu-(COOH)btc-HKUST-1.

S1.3. Computational methods

S2. Characterization of RhCu-btc-HKUST-1.

S3. Characterization of Cu(II)-HKUST-1.

S4. Hydrolytic stability study of RhCu-btc-HKUST-1 and Cu(II)-HKUST-1.

S4.1. DFT calculations of Rh(II) and Cu(II) paddlewheels in water

S5. Characterization of RhCu-(Br)btc-HKUST-1.

S6. Characterization of RhCu-(NO_2)btc-HKUST-1.

S7. Characterization of RhCu-(NH_2)btc-HKUST-1.

S8. Characterization of RhCu-(COOH)btc-HKUST-1.

S9. References

S1. Materials and methods

S1.1 Materials and characterization

Rhodium acetate, sodium carbonate, 1,3,5-Benzenetricarbonyl trichloride, 2-(trimethylsilyl)ethanol, anhydrous pyridine, tetrabutylammonium fluoride (1.0 M in tetrahydrofuran (THF)), sodium hydroxide, copper(II) nitrate trihydrate and methylene blue (MB) were purchased from SigmaAldrich. 2-bromomesitylene, 2-nitromesitylene, and 1,2,3,5-tetramethylbenzene were purchased from Abcr. 2-Aminobenzene-1,3,5-tricarboxylic acid was purchased from Chem Extension. Solvents at HPLC grade were purchased from Fischer Chemicals. All deuterated solvents were purchased from Eurisotop. All reagents and solvents were used without further purification. Deionized water was obtained with a Milli-Q® system (18.2 MΩ·cm).

Field-Emission scanning electron microscopy (FESEM) was performed in an FEI Quanta 650FEG ESEM, and a FEI Magellan 400L XHR SEM. The average size distribution of each synthesized HKUST-1 analogue was statistically estimated from FESEM images by counting the sizes of 150 particles at different areas from each sample.

Transmission electron microscopy-energy Dispersive X-ray spectroscopy (TEM-EDX) was performed in an FEI Tecnai G2 F20 S-TWIN HRSTEM.

Ultraviolet-visible (UV−vis) spectra were measured using a Thermo Scientific™ NanoDrop 2000 at room temperature (ca. 25 °C).

Proton nuclear magnetic resonance (^{1}H-NMR) spectra were acquired in a Bruker Ascend 300 MHz and a Bruker Avance III 400SB NMR spectrometer at “Servei d’Análisi Química” from Autonomus University of Barcelona (UAB).

Solid state nuclear magnetic resonance spectroscopy (ssNMR) spectra were obtained on a Bruker Avance NEO 400 using a 4 mm triple resonance magic angle spinning (MAS) probe. The MAS rotor spinning was set to 12 kHz and the samples were measured for 2323 scans in cross-polarization (CP) mode. The chemical shifts are reported in ppm and are referenced external for ^{13}C to adamantane.

Powder X-ray diffraction (PXRD) diagrams were collected on a Panalytical X’pert diffractometer with monochromatic Cu-K_α radiation (λ_{Cu} = 1.5406 Å). Samples were measured in a capillary.

Synchrotron powder X-ray diffraction (PXRD) of RhCu-btc-HKUST-1 in a colloidal solution was collected at the XALOC-BL13 beamline of the Alba Synchrotron Facility.[2] The experiment was carried out in transmission mode using a radiation with a wavelength, λ = 0.82653 Å directing the beam at the bottom of the capillary, with higher density of nanoparticles. Data were collected using a photon-counting PILATUS 6M DECTRIS pixel-array detector. The diffraction pattern was radially integrated using FIT2D Program.[3]

Rietveld refinement was carried out with the use of the Reflex Module of Materials Studio. Powder X-ray diffraction data was collected at the beamline XALOC-BL13 beamline of the Alba Synchrotron Facility, in transmission mode. A pseudo-Voigt function was used for profile description, with a 20-coefficient polynomial order background, and refinement of crystallize size for peak broadening description. Rhodium and copper atoms were placed at the same crystallographic site with 50% occupancy. Solvent water molecules were added in the pores, and their position refined.

X-ray Pair Distribution Function (PDF) analyses were performed using Synchrotron X-ray total scattering data suitable for PDF analyses collected at the I15-1 beamline (0.1617 Å, 76 keV) at the Diamond Light Source synchrotron. Samples were ground into borosilicate capillaries (1 mm i.d.) and sealed. Data scans were collected during 10 minutes. Empty capillary and background total scattering data were corrected for data processing. Geometric corrections and reduction to 1D data used DAWN Science software.[4] Total PDF data were obtained from the corrected total scattering data using PDFgetX3[5] within xPDFsuite[6] to a Qmax=25 Å−1. Differential pair distribution function (dPDF) data were obtained in real-space, after applying a normalization factor to the total PDF data.

X-ray photoelectron spectroscopy (XPS) measurements were performed at room temperature with a SPECS PHOIBOS 150 hemispherical analyzer (SPECS GmbH, Berlin, Germany)) in a base pressure of $5x10^{-10}$ mbar using monochromatic Al-K_α radiation (1486.74 eV) as excitation source operated at 300 W. The energy resolution was measured by the FWHM of the Ag $3d_{5/2}$ peak for a sputtered silver foil was 0.62 eV. The spectra were calibrated with respect to the C1s at 284.8 eV.

Thermogravimetric analysis (TGA) curves were carried out in a Pyris Perkin Elmer TGA 8000 under nitrogen atmosphere and a heating rate of 10 °C/min.

Volumetric N_2 isotherms were collected at 77 K using an ASAP 2460 and High Resolution ASAP 2020 (Micromeritics). Temperature for N_2 isotherms measurement was controlled by using a liquid nitrogen bath. BET surface values were calculated according to the BETSI method.[1]

Inductively coupled plasma mass spectrometry (**ICP-MS**) analyses were performed in an external company, Leitat, in an ICP-MS Agilent 7500. 5 mg of sample were digested with 4 mL of concentrated ultrapure nitric acid (HNO_3 70%) in an analytical microwave at 250 °C. Subsequently, the digestion residue obtained was suitably diluted to analyze the elements of interest by ICP-MS. The quantification was performed by interpolation on a calibration curve prepared from commercial standards of the elements of interest. The assays were performed in duplicate.

Matrix-assisted laser desorption/ionization-time of flight (MALDI-TOF) mass spectrometry (MS) measurements were performed using a 4800 Plus MALDI TOF/TOF (ABSCIEX – 2010) operating in positive-ionization mode using sinapinic acid as ionization matrix.

S1.2 Experimental Methods

S1.2.1 Synthesis of COOH-RhMOP, (Br)btc, (NO_2)btc and (COOH)btc.

COOH-RhMOP was synthesized according to a previously described methodology.[7] 2-bromobenzene-1,3,5-tricarboxylic acid ((Br)btc), 2-nitrobenzene-1,3,5-tricarboxylic acid ((NO_2)btc), and 1,2,3,5-benzenetetracarboxylic acid (COOH)btc were synthesized according to the previously described methodologies.[8]

S1.2.2 Stability of COOH-RhMOP under solvothermal conditions.

5 mg of COOH-RhMOP were dissolved in 2 mL of DMF-d_7 and the resulting green solution was heated at 85ºC for 12 h. After this incubation time, the green solution was analyzed through mass spectrometry, UV-Vis, and ^{1}H-NMR to confirm the integrity of COOH-RhMOP.

S1.2.3 Synthesis of RhCu-btc-HKUST-1, RhCu-(Br)btc-HKUST-1, RhCu-(NO_2)btc-HKUST-1, RhCu-(NH_2)btc-HKUST-1 and RhCu-(COOH)btc-HKUST-1.

RhCu-btc-HKUST-1 ($[Rh_{1.5}Cu_{1.5}(btc)_2(H_2O)_3]$). A N,N-dimethylformamide (DMF) solution (0.5 ml) containing 3.9 mg (16.02 μmol) of copper nitrate trihydrate ($Cu(NO_3)_2{\cdot}3H_2O$) was added to a DMF solution (1 ml) containing 5 mg (0.67 μmol) of COOH-RhMOP. Next, a DMF solution (0.5 ml) containing 1.12 mg (5.33 μmol) of trimesic acid (btc) was added to the former solution. The resulting green mixture was heated at 85 °C for 24 h. The obtained suspension was centrifuged to obtain a green solid that was washed with DMF (x3), methanol (x3), water (x5), and acetone (x2), and dried at room temperature for 6 h. Elemental analysis for $[Rh_{1.5}Cu_{1.5}(btc)_2(H_2O)_3]{\cdot}2$Acetone: C, 34.28 %; H, 3.57 %; N, 0 %. Found: C, 34.42 %; H, 3.60 %; N, 0.1 %.

The sample was exchanged with acetone for 3 days and activated at 120ºC under vacuum for 12 h before the gas adsorption measurements. The yield of the reaction was calculated from the activated phase. The obtained yield was 70%.

RhCu-(Br)btc-HKUST-1 ($[Rh_{1.5}Cu_{1.5}(btc)_{1.5}(Brbtc)_{0.5}(H_2O)_3]$). A DMF solution (0.5 ml) containing 7.8 mg (32.04 μmol) of $Cu(NO_3)_2{\cdot}3H_2O$ was added to a DMF solution (1 ml) containing 10 mg (1.34 μmol) of COOH-RhMOP. Next, a DMF solution (0.5 ml) containing 3.09 mg (10.69 μmol) of (Br)btc was added to the former solution. The resulting green mixture was heated at 120 °C for 6 h. The obtained suspension was centrifuged to obtain a green solid that was washed with DMF (x3), methanol (x3), water (x5), and acetone (x2), and dried at room temperature for 6 h. Elemental analysis for $[Rh_{1.5}Cu_{1.5}(btc)_{1.5}(Brbtc)_{0.5}(H_2O)_3]{\cdot}H_2O{\cdot}0.5$DMF: C, 26.84 %; H, 3.33 %; N, 0.80 %. Found: C, 26.87 %; H, 2.96 %; N, 0.65 %.

The sample was exchanged with acetone for 3 days and activated at 120ºC under vacuum for 12 h before the gas adsorption measurements. The yield of the reaction was calculated from the activated phase. The obtained yield was 82%.

RhCu-(NO_2)btc-HKUST-1 ($[Rh_{1.5}Cu_{1.5}(btc)_{1.5}(NO_2btc)_{0.5}(H_2O)_3]$). A DMF solution (0.5 ml) containing 7.8 mg (32.04 μmol) of $Cu(NO_3)_2{\cdot}3H_2O$ was added to a DMF solution (1 ml) containing 10 mg (1.34 μmol) of COOH-RhMOP. Next, a DMF solution (0.5 ml) containing 2.72 mg (10.69

μmol) of (NO_2)btc was added to the former solution. The resulting green mixture was heated at 100ºC for 8 h. The obtained suspension was centrifuged to obtain a green solid that was washed with DMF (x3), methanol (x3), water (x5), and acetone (x2), and dried at room temperature for 6 h. Elemental analysis for $[Rh_{1.5}Cu_{1.5}(btc)_{1.5}(NO_2btc)_{0.5}(H_2O)_3]\cdot 3H_2O\cdot 1.5$Acetone: C, 30.39 %; H, 3.66 %; N, 0.79 %. Found: C, 30.58 %; H, 3.24 %; N, 0.67 %.

The sample was exchanged with acetone for 3 days and activated at 120ºC under vacuum for 12 h before the gas adsorption measurements. The yield of the reaction was calculated from the activated phase. The obtained yield was 78%.

RhCu-(NH_2)btc-HKUST-1 ($[Rh_{1.5}Cu_{1.5}(btc)_{1.5}(NH_2btc)_{0.5}(H_2O)_3]$). A DMF solution (0.5 ml) containing 7.8 mg (32.04 μmol) of $Cu(NO_3)_2\cdot 3H_2O$ was added to a DMF solution (1 ml) containing 10 mg (1.34 μmol) of COOH-RhMOP. Next, a DMF solution (0.5 ml) containing 2.4 mg (10.69 μmol) of (NH_2)btc was added to the former solution. The resulting green mixture was heated at 85ºC for 24 h. The obtained suspension was centrifuged to obtain a green solid that was washed with DMF (x3), methanol (x3), water (x5), and acetone (x2), and dried at room temperature for 6 h. Elemental analysis for $[Rh_{1.5}Cu_{1.5}(btc)_{1.5}(NH_2btc)_{0.5}(H_2O)_3]\cdot 2H_2O\cdot 1.5$Acetone: C, 31.59 %; H, 3.69 %; N, 0.82 %. Found: C, 31.46 %; H, 3.72 %; N, 0.92 %.

The sample was exchanged with acetone for 3 days and activated at 120ºC under vacuum for 12 h before the gas adsorption measurements. The yield of the reaction was calculated from the activated phase. The obtained yield was 74%.

RhCu-(COOH)btc-HKUST-1 ($[Rh_{1.5}Cu_{1.5}(btc)_{1.5}(COOHbtc)_{0.5}(H_2O)_3]$). A DMF solution (0.5 ml) containing 7.8 mg (32.04 μmol) of $Cu(NO_3)_2\cdot 3H_2O$ was added to a DMF solution (1 ml) containing 10 mg (1.34 μmol) of COOH-RhMOP. Next, a DMF solution (0.5 ml) containing 2.71 mg (10.69 μmol) of (COOH)btc was added to the former solution. The resulting green mixture was heated at 85ºC for 24 h. The obtained suspension was centrifuged to obtain a green solid that was washed with DMF (x3), methanol (x3), water (x5), and acetone (x2), and dried at room temperature for 6 h. Elemental analysis for $[Rh_{1.5}Cu_{1.5}(btc)_{1.5}(COOHbtc)_{0.5}(H_2O)_3]\cdot 2H_2O\cdot$Acetone: C, 30.71 %; H, 3.33 %; N, 0 %. Found: C, 30.76 %; H, 3.59 %; N, 0.1 %.

The sample was exchanged with acetone for 3 days and activated at 120ºC under vacuum for 12 h before the gas adsorption measurements. The yield of the reaction was calculated from the activated phase. The obtained yield was 65%.

S.1.2.4 Blank reactions for RhCu-btc-HKUST-1.

All blank reactions were conducted in analogy to the procedure used for the synthesis of RhCu-btc-HKUST-1, but without the presence of one of the components.

Copper(II) with btc. A DMF solution (1.5 ml) containing 3.9 mg (16.02 μmol) of $Cu(NO_3)_2\cdot 3H_2O$ was added into a DMF solution (0.5 ml) containing 1.12 mg (5.33 μmol) of btc. The resulting light blue solution was heated to 85 °C for 24 h. The obtained suspension was centrifuged, and the obtained parent HKUST-1 was washed with DMF (x3), and methanol (x3), and dried at room temperature for 6 h.

Copper(II) with COOH-RhMOP. A DMF solution (1 ml) containing 3.9 mg (16.02 μmol) of $Cu(NO_3)_2\cdot 3H_2O$ was added to a DMF solution (1 ml) containing 5 mg (0.67 μmol) of COOH-

RhMOP. The resulting green solution was heated up to 85 °C for 24 h. The obtained suspension was centrifuged, and the solid was washed with DMF (x3), methanol (x3), water (x5), and acetone (x2), and dried at room temperature for 6 h. The powder consisted in an amorphous solid. Note here that our previously reported RhCu-rht-MOF crystals were not obtained as the blank reaction conditions differ from the optimized reaction conditions to obtain the RhCu-rht-MOF.[9]

COOH-RhMOP with btc. A DMF solution (1 ml) containing 5 mg (0.67 µmol) of COOH-RhMOP was added to a DMF solution (1 ml) of 1.12 mg (5.33 µmol) of btc. The starting green solution was heated up to 85 °C and kept for 24 h. After this time, no precipitate was observed.

COOH-RhMOP with Cu(II)-HKUST-1. 15 mg of Cu(II)-HKUST-1 were added to a DMF solution (2 ml) containing 10 mg (1.34 µmol) of COOH-RhMOP. The starting green suspension was heated up to 85 ºC and kept for 24 h. The obtained suspension was centrifuged to obtain a light blue solid that was washed with DMF (x3) and methanol (x3) and dried at room temperature for 6 h. The crystalline product was further characterized by FESEM and PXRD.

S.1.2.5 Acidic disassembly of RhCu-btc-HKUST-1.

Acidic disassembly of RhCu-btc-HKUST-1. In a typical experiment, 5 mg of RhCu-btc-HKUST-1 were dispersed in 500 µL DMF-d_7. 5 µL of a 3M stock solution of deuterium chloride (DCl) were added to this dispersion. The mixture was gently stirred until RhCu-btc-HKUST-1 was completely dissolved forming a green solution. This solution was then analyzed through ^{1}H-NMR to determine the COOH-RhMOP:ligand molar ratio.

Isolation of pure COOH-RhMOP from RhCu-btc-HKUST-1. The identification of COOH-RhMOP extracted from RhCu-btc-HKUST-1 required to obtain it in its pure isolated form; that is, without Cu(II) or btc linkers. To this end, the acidic disassembly was slightly modified. In a typical experiment, 10 mg of RhCu-btc-HKUST-1 were incubated in 0.12M hydrogen chloride (HCl) aqueous solution for 4 h at room temperature. After this incubation time, the sample was washed with water to completely remove Cu(II) ions that were detached from the structure due to the protonation of all the carboxylic groups of COOH-RhMOP and btc. Next, free btc linkers were removed by washing the sample with a mixture of tetrahydrofuran:diethyl ether (x2) and chloroform ($CHCl_3$) (x4). The resulting green solid was incubated in diethyl ether overnight to completely remove the remaining $CHCl_3$. Finally, the solid was dried at 60ºC for 2 h. The solid was redissolved in DMF to analyze it through ^{1}H-NMR, UV-Vis, and mass spectrometry to corroborate that the resulting solid is pure COOH-RhMOP (Figure S14 and S15). UV-Vis analysis of this solution was used to determine its concentration and the efficiency of the extracting protocol (Figure S17).

S.1.2.6 Acidic digestion of RhCu-(Br)btc-HKUST-1, RhCu-(NO_2)btc-HKUST-1 and RhCu-(NH_2)btc-HKUST-1 and RhCu-(COOH)btc-HKUST-1.

Acidic digestion of RhCu-(Br)btc-HKUST-1. 5 mg of RhCu-(Br)btc-HKUST-1 were dispersed in a mixture of 500 µL DMSO-d_6 and 20 µL DCl (20 wt% in D_2O) at room temperature. Complete digestion was achieved after 4 weeks of incubation time. Note that, in this case, high temperatures were not applied to the digestion mixture to avoid the degradation of the (Br)btc linker.

Acidic digestion of RhCu-(NO_2)btc-HKUST-1, RhCu-(NH_2)btc-HKUST-1 and RhCu-(COOH)btc-HKUST.-1. 10 mg of each HKUST-1 analogues were dissolved in a mixture of 500 µL DMSO-d_6 and 20 µL DCl (20 wt% in D_2O). The mixture was heated at 100°C for 2 h to achieve complete digestion of the sample

S1.2.7 Study of the hydrolytic stability of RhCu-btc-HKUST-1 and Cu(II)-HKUST-1.

RhCu-btc-HKUST-1. 40 mg of sample were incubated in 8 ml of water. After the desired incubation period the sample was removed from water, briefly washed with methanol and dryed at 85ºC for 30 min. The obtained solid was analyzed through PXRD, FESEM, and volumetric N_2 adsorption. The sample incubated for 31 days was also fully digested and analyzed through ICP-MS, which confirmed that the Cu:Rh molar ratio was kept constant after the water incubation period. Thus, the Cu:Rh molar ratio of the sample incubated in water for 31 days was found to be 1.07± 0.02.

The water-incubated samples were exchanged with acetone for 3 days and activated at 120ºC under vacuum for 12h before the gas adsorption measurements.

Cu(II)-HKUST-1. 40 mg of sample were incubated in 8 ml of water. After the desired incubation period the sample was removed from water, briefly washed with methanol and dryed at 85ºC for 30 min. The obtained solid was analyzed through XRPD, FESEM, and volumetric N_2 adsorption.

The water-incubated samples were exchanged with acetone for 3 days and activated at 120ºC under vacuum for 12 h before the gas adsorption measurements.

S1.2.8. Study of the methylene blue removal with RhCu-btc-HKUST-1 and Cu(II)-HKUST-1.

The methylene blue (MB) removal experiments were performed using the following protocol. An aqueous solution containing 2 mg (6.25 µmol) of MB (20 ppm concentration) was prepared and the UV-vis absorption spectrum of the initial solution was recorded. 5 mg of activated sample were introduced into the MB aqueous solution, and the MB concentration was monitored every 3 min until 60 min by UV-Vis. The MB concentration at each point was determined using the absorbance at 665 nm. The MB removal was calculated using the following equation:

$$Removal\ of\ MB\ (\%) = \left(\frac{C_0 - C}{C_0}\right) x100$$

In order to test the crystallinity of these samples after the adsorption experiment, their PXRD patterns were measured directly without additional washings after the absorption experiment.

The adsorption of MB over several cycles was performed as follows. After each adsorption experiment, the adsorbent (*i.e.* RhCu-btc-HKUST-1 or Cu(II)-HKUST-1) was recovered by centrifugation. The solid was washed wit mater (x1) and acetone (x2), and finally dried at 85ºC under vacuum. The regenerated adsorbent was added to a fresh 20 ppm MB solution, from which the uptake was measured after 1 hour incubation time. This process was repeated over 5 cycles of adsorption/regeneration.

S1.2.9 Study of the catalytic activity of RhCu-btc-HKUST-1 and RhCu-(COOH)btc-HKUST-1.

The catalytic activity of RhCu-btc-HKUST-1 and RhCu-(COOHbtc-HKUST-1 was studied using the following protocol. The catalytic test was carried out in a screw vial loaded with 13 mg of catalyst. Prior the catalytic test, the catalyst was activated at 85ºC under vacuum for 1 hour. A stock solution of benzaldehyde dimethyl acetal (1 mmol, 1 eq.) and 1,3,5-trimethoxybenzene (0.33 mmol, 0.33 eq.) was prepared in a total amount of 1 mL acetone/D_2O mixture (10% water content). The 1,3,5-trimethoxybenzene acts as an internal standard used to quantify the degree of conversion after each measured time. To start the reaction, the stock solution was added in one shot to the catalyst and stirred constantly at 60 ºC. The reaction was monitored as follows. After each reaction time, 100 µL of the reaction mixture were taken and centrifuged. 50 µL of the supernatant were diluted in d^6-acetone (400 µL). To keep the catalyst loading constant, the catalyst recovered after centrifugation were added back to the reaction mixture.

S1.3 Computational Methods

All simulated structure were generated with Materials Studio 8.0 Program from the crystallographic structure of Cu(II)-HKUST-1.

Computational simulation of the stability of Cu(II) and Rh(II) paddlewheels clusters in water were performed as follows. We have performed DFT geometry optimizations of metal (Cu(II) or Rh(II)) acetate paddlewheel structures in water solvent. These paddlewheel structures are the inorganic MBBs that build up the RhCu-btc-HKUST-1 and the parent HKUST-1 MOFs discussed in the main paper. All calculations are performed in water solvent to model the behavior of Cu(II) and Rh(II) paddlewheels when they are exposed to water. To this end, we have employed Gaussian 16 software[10] and the optimizations were performed at the M06-L/SDD level of theory[11] in presence of implicit water solvent, modeled with the Polarizable Continuum Model (PCM) using the integral equation formalism variant (IEFPCM),[12] which is the default solvent model in Gaussian.

We selected the M06-L hybrid functional of Truhlar and Zhao[10] because of its broad accuracy for metallochemical calculations for all metals. Previous calculations of Cu(II) acetate paddlewheel structures in vacuo comparing different DFT functionals and basis sets with MP2 ab initio quantum chemistry calculations[13] showed that MP06 is the best performing functional irrespective of the basis set employed and energies and geometrical quantities (bond distances, angles...) calculated with this functional show little dependence on the specific basis set employed. For our calculations, we selected the commonly used SDD basis set because it is suitable for both Cu(II) and Rh(II) and acetate. It combines double zeta with the Stuttgart-Dresden ECP, which reduces the cost caused by a large number of electrons, giving close agreement with experimental results.[14]

We have considered four different structure optimizations, corresponding to two different sets of simulations. In the first set of calculations, we have considered the optimization of a metal (Cu(II) or Rh(II)) acetate paddlewheel in implicit water, considering two metal ions and four acetate molecules. In the second set of simulations, we have considered the effect of adding explicit coordinating water molecules interacting with the metals of the paddlewheel at the (free) axial positions. These calculations are also performed in implicit water solvent. All images reported here are made with VMD software.[15]

S2. Characterization of RhCu-btc-HKUST-1.

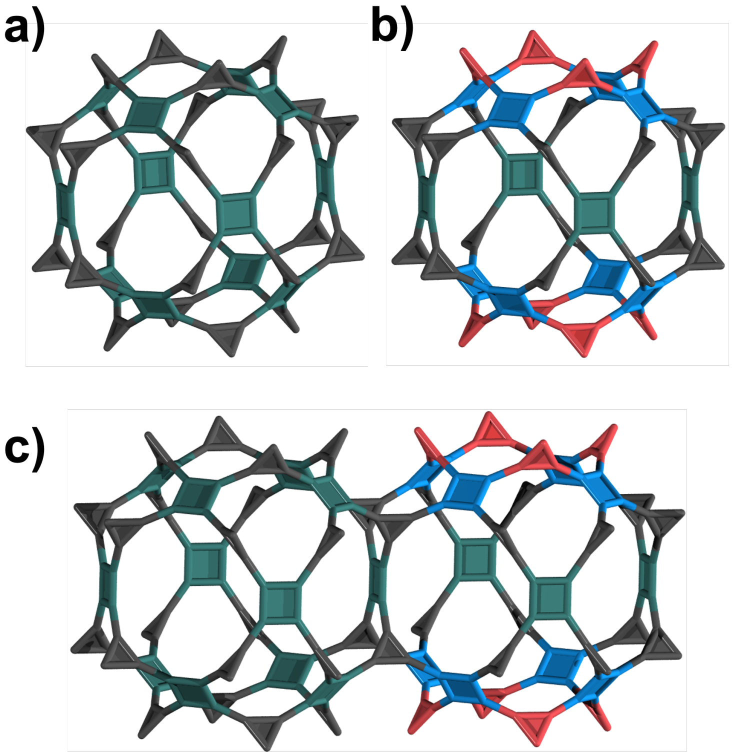

Figure S1 Schematic representation of the two types of cuboctahedral cavities alternated in the structure: (a) Rh(II)-based cavity coming from the prefabricated cavity; and (b) a mixed-metal cavity containing Cu(II) and Rh(II) ions generated upon the self-assembly of the network. (c) Schematic representation of how the two types of cuboctahedral cavities are connected within the RhCu-btc-HKUST-1 structure. Atom color code: Rh(II), green; Cu(II), blue; btc linker from the COOH-RhMOP cavity, grey; and btc linker added as an isolated ligand during the synthesis of RhCu-btc-HKUST-1, red.

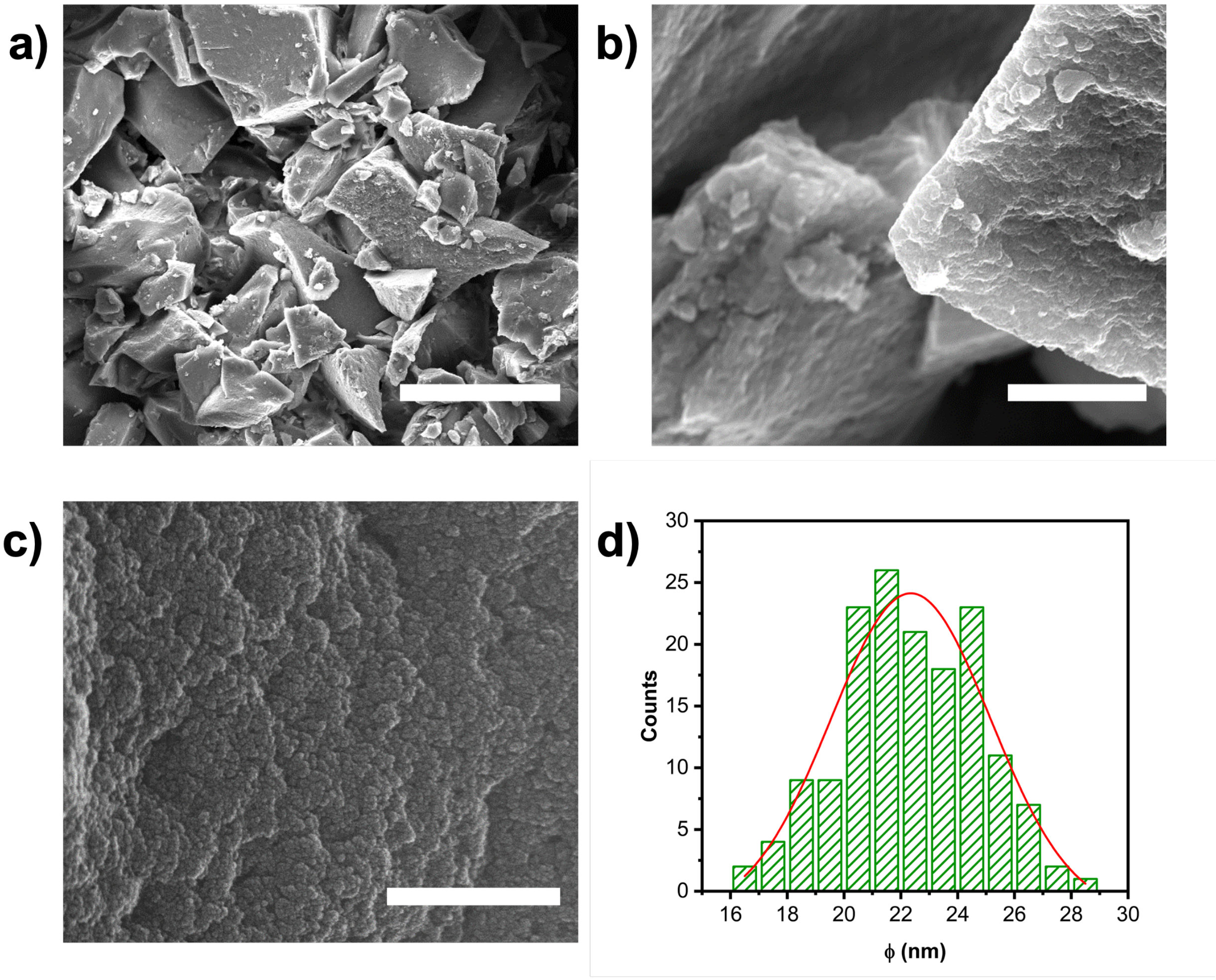

Figure S2 (a,b) FESEM images of the micrometric monolithic aggregates composed of RhCu-btc-HKUST-1 nanoparticles. These monolithic aggregates are formed upon drying the colloidal dispersion of RhCu-btc-HKUST-1 nanoparticles. c) Close view of the aggregated RhCu-btc-HKUST-1 nanoparticles. d) Particle size distribution histogram of RhCu-btc-HKUST-1 with a mean particle size of 22 ± 3 nm. Scale bars: 100 μm (a) 5 μm (b), and 500 nm (c).

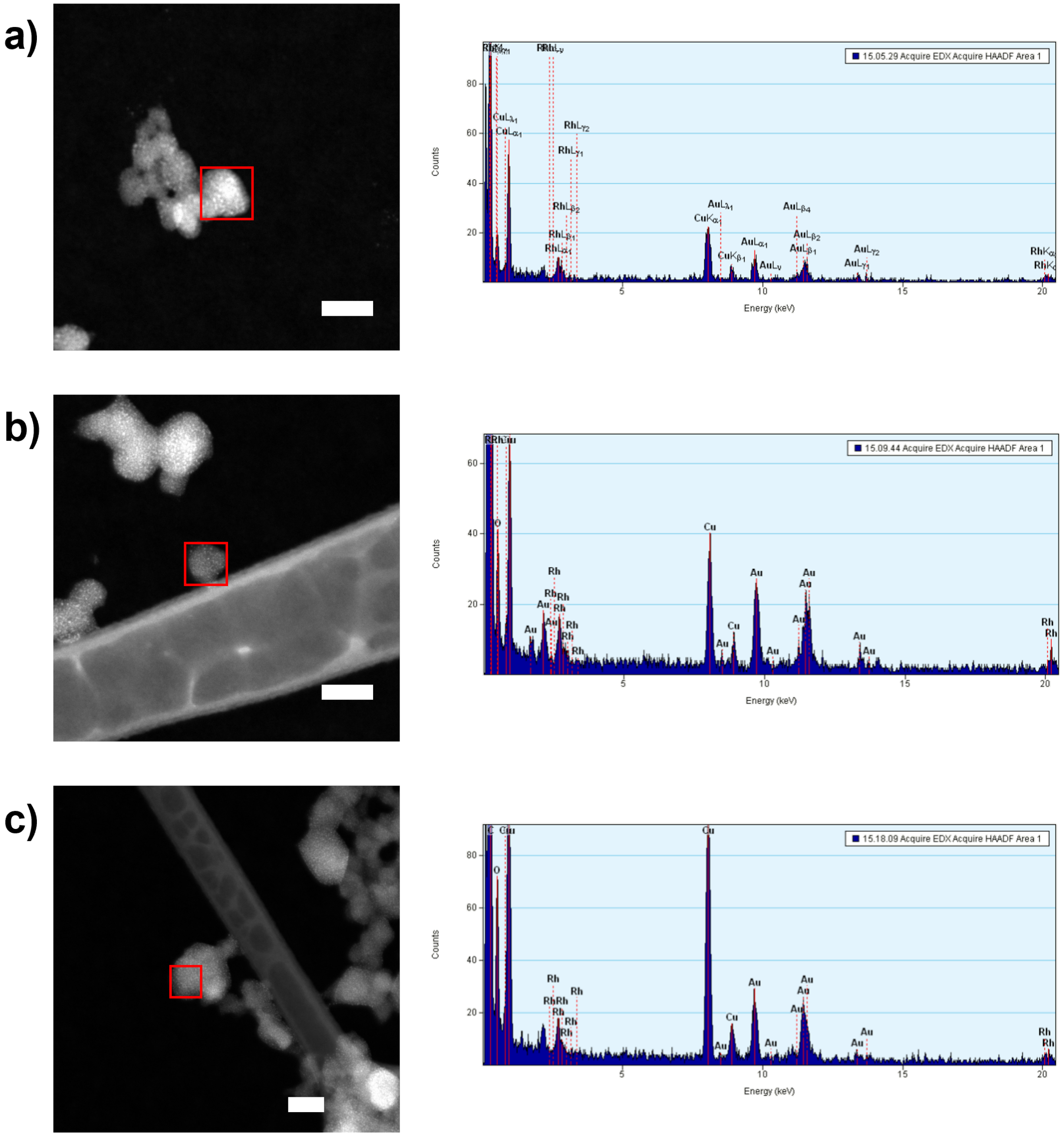

Figure S3 (a-c) Representative HAADF-STEM images (left) and the corresponding EDX spectra (right) obtained from single RhCu-btc-HKUST-1 nanocrystals. Scale bars: 50 nm (a, b, c). Note that Au signals stem from the TEM grid used for these measurements.

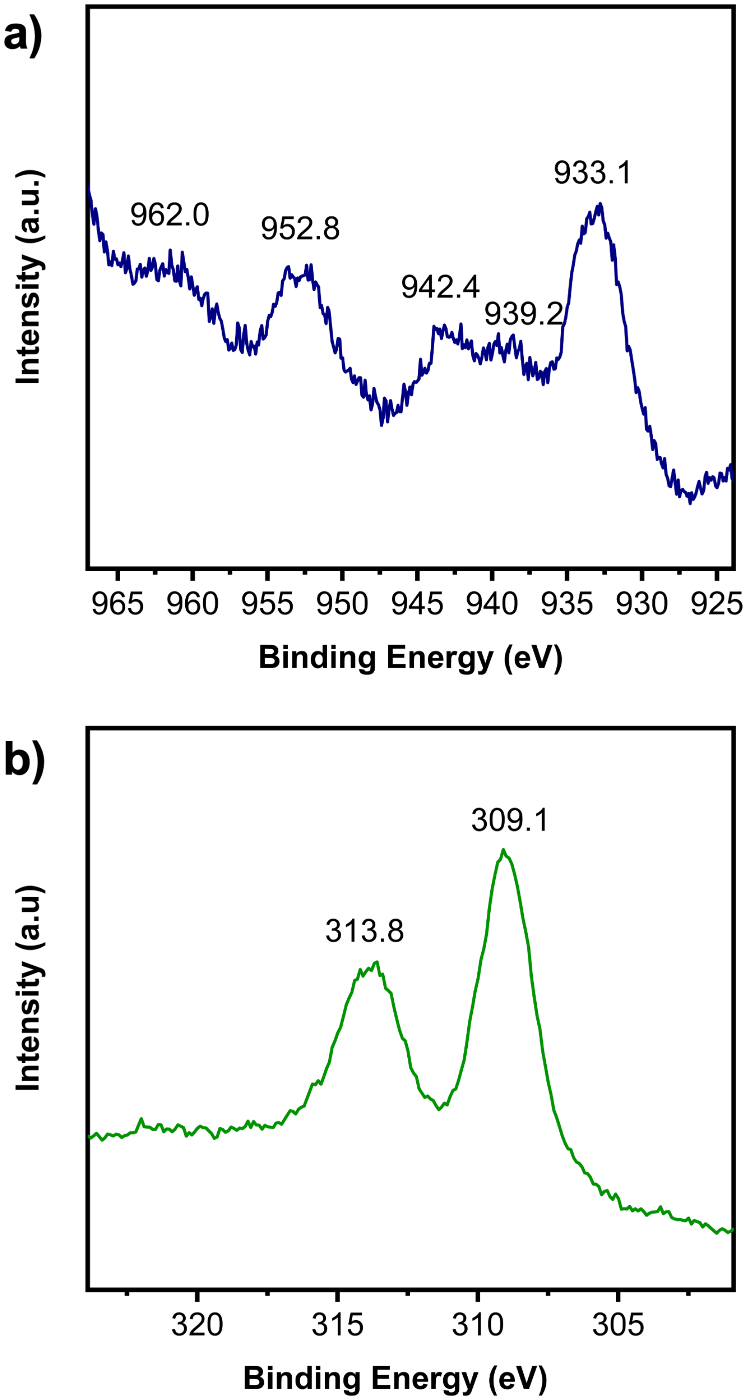

Figure S4 XPS spectra of (a) Cu 2p and (b) Rh 3d of RhCu-btc-HKUST-1. The values of 933.3 and 952.8 in (a) are consistent with those of Cu(II). Herein, the presence of satellites also confirms that copper exists as Cu(II).[16] The values of 309.1 and 313.8 in (b) are consistent with those of Rh(II), confirming that rhodium exists as Rh(II).[17]

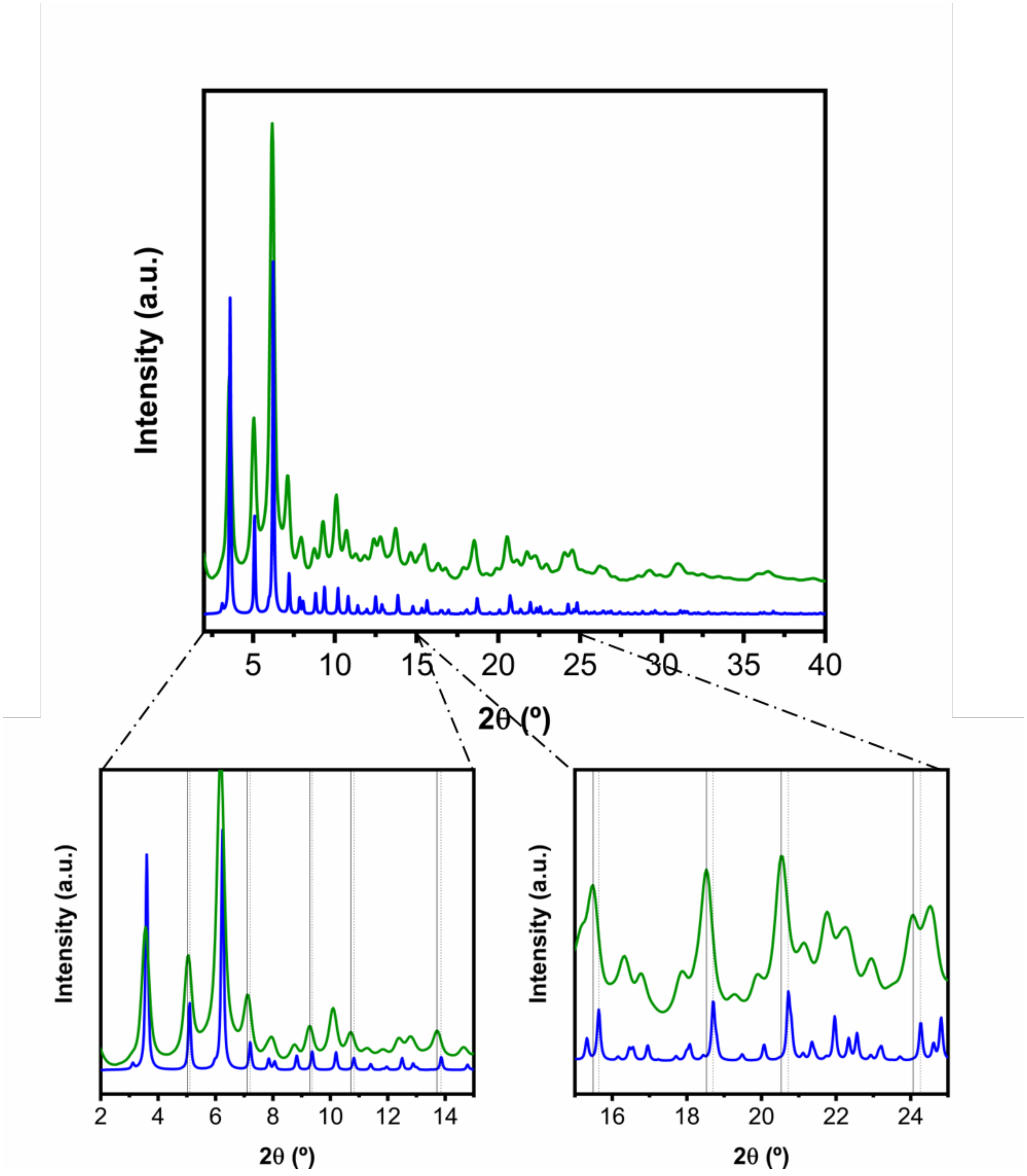

Figure S5 Simulated PXRD of Cu(II)-HKUST-1 (blue) and synchrotron PXRD measured in a capillary of RhCu-btc-HKUST-1 (green). Amplified regions of the diffractogram from 2 to 15 degrees and from 15 to 25 degrees are presented to highlight the shift between the simulated and the experimental pattern, which indicates slight differences in the lattice parameters.

Table S1. Rietveld refinement parameters of RhCu-btc-HKUST-1

Sample:	RhCu-btc-HKUST-1			
Wavelength	0.82653 Å			
Crystal System	Cubic			
Space group	*Fm-3m*			
Lattice parameter	a = 26.5772 ±0.0053 Å			
Crystallite size	239.774 ±3.25078 Å			
Profile parameters (Pseudo-Voigt)	U = 2.00174; V = -0.25998; W= 0.01836; NA = 0.03972			
Atom sites	x	y	z	Occupancy
O2	0.18336	0.25687	0.94776	1.0
C1	0.17800	0.67800	0.88700	1.0
C2	0.20320	0.29680	0.93130	1.0
H1	0.11980	0.38020	0.77200	1.0
C3	0.19940	0.63450	0.86550	1.0
Cu1	0.21471	0.50000	0.71471	0.5
O1	0.34340	0.50000	0.84340	1.0
Rh	0.21471	0.50000	0.71471	0.5
Ow	0.08543	0.50000	0.41457	1.0
Final Rwp	4.68%			
Final Rp	3.32%			
Final CMACS	2.28%			
Final Rwp (w/o bckg)	22.17%			

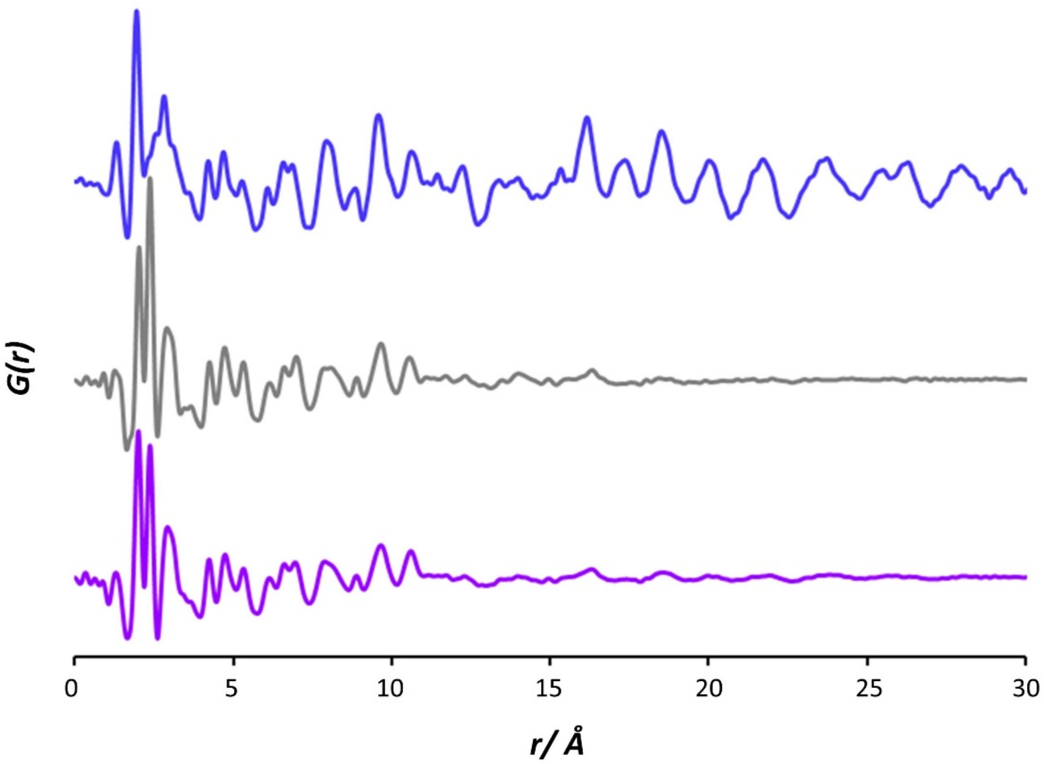

Figure S6 Total PDF data of Cu-HKUST-1 (blue), COOH-RhMOP (grey) and RhCu-HKUST-1 (purple). The total PDF of the COOH-RhMOP shows characteristic signals at the local scale (< 6 Å) corresponding to the Rh(II) centers at ~2.04 Å (Rh-O) and ~2.38 Å (Rh···Rh), while for Cu-HKUST-1 analogue signals are observed at ~1.96 Å (Cu-O) and ~2.5 Å (Cu···Cu).

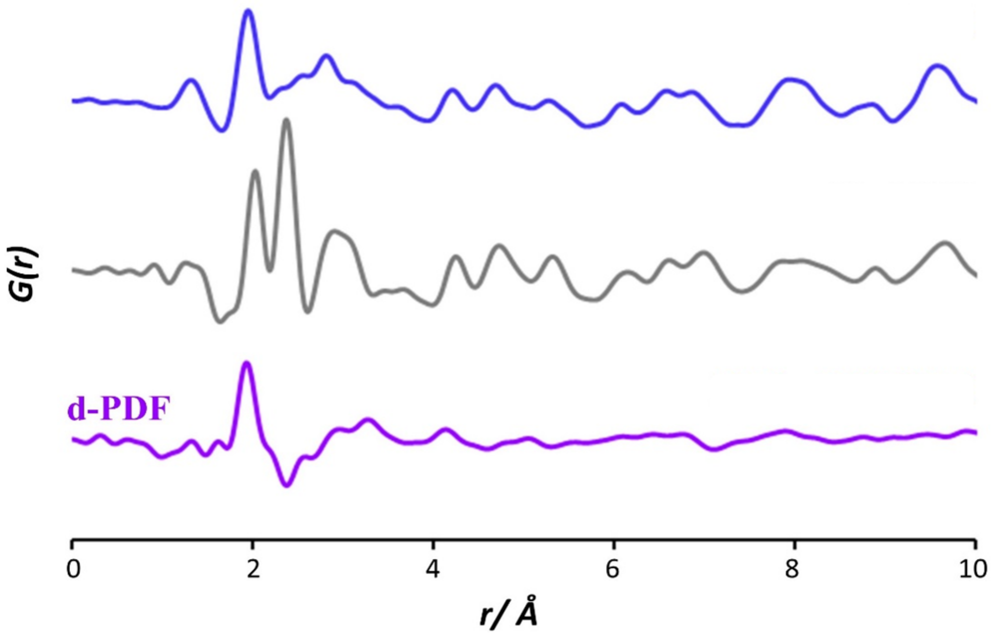

Figure S7. Detail of the total PDF data of Cu-HKUST-1 (blue) and COOH-RhMOP (grey), together with the d-PDF data obtained for RhCu-HKUST-1 (purple). The differential PDF analysis (d-PDF) was performed by subtracting the total PDF data of the COOH-RhMOP to that of the RhCu-HKUST-1. The obtained d-PDF spectrum showed signals at ~1.9 Å and ~2.6 Å, attributed to Cu−O and Cu···Cu distances, respectively. Therefore, the absence of contributions that could be associated with Cu(II)···Rh(II) distances demonstrated the lack of heterobimetallic Rh(II)/Cu(II) paddlewheel clusters within the RhCu-btc-HKUST-1 network.

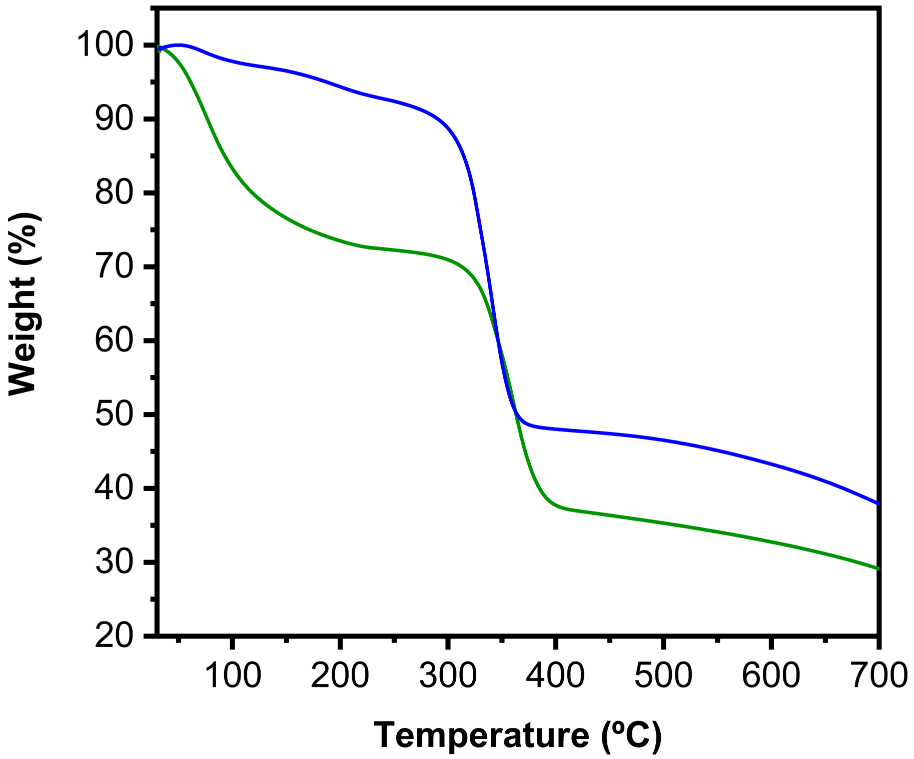

Figure S8 Thermogravimetric analysis of as-made (green) and activated (blue) RhCu-btc-HKUST-1. Note that the difference between the two analysis is associated to the remaining solvent molecules coordinated to the metal sites. The decomposition temperature of RhCu-btc-HKUST-1 (325 ºC) is in good agreement to reported values for the parent Cu(II)-HKUST-1.[18]

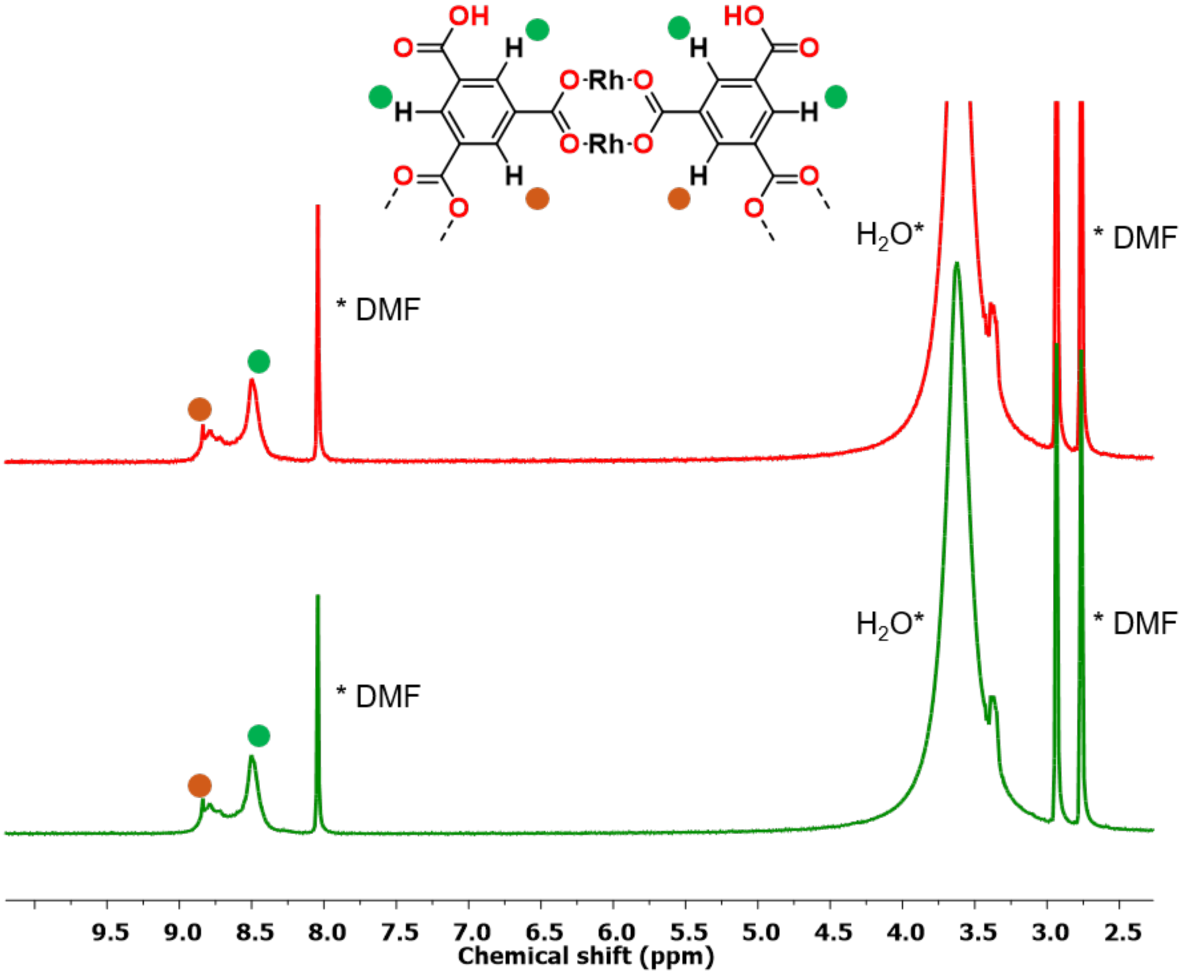

Figure S9 ^{1}H NMR spectra (400 MHz, DMF-d_7) of COOH-RhMOP before (green) and after (red) incubating it at 85ºC for 12 h. Note that, under the synthetic conditions used for the synthesis of RhCu-btc-HKUST-1, COOH-RhMOP maintains its integrity.

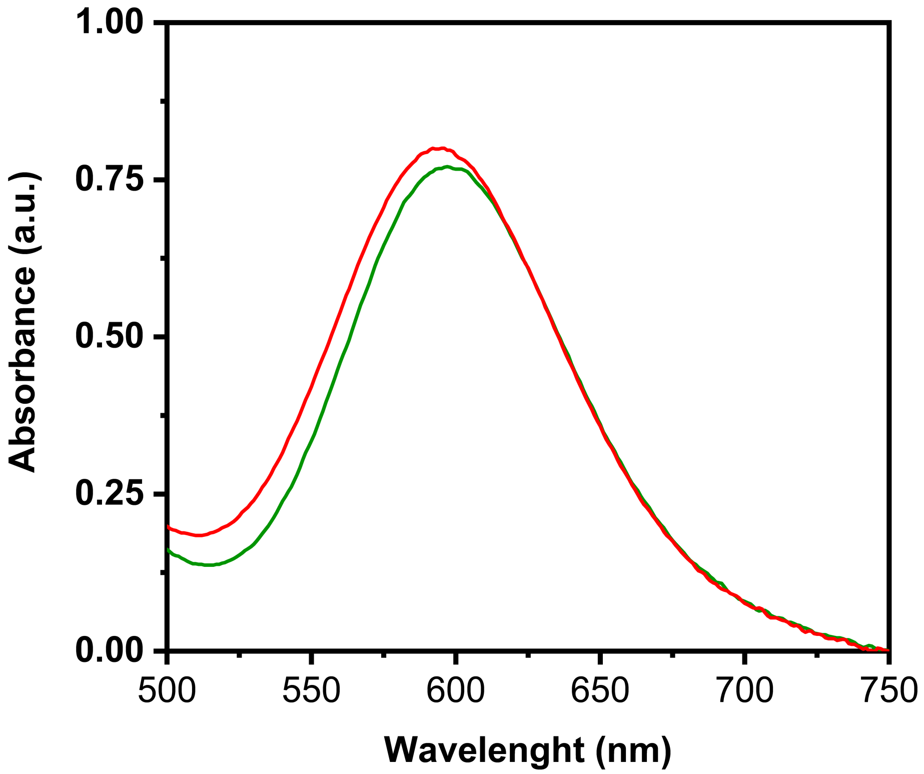

Figure S10 UV-Vis absorption data of a DMF solution of COOH-RhMOP before (green) and after (red) incubating it at 85ºC for 12 h. The unchanged position of λ_{max} at 595 nm demonstrates the integrity of the Rh-Rh paddlewheel. The slightly higher absorbance observed for the post-incubated sample is ascribed to eventual DMF evaporation during the incubation time.

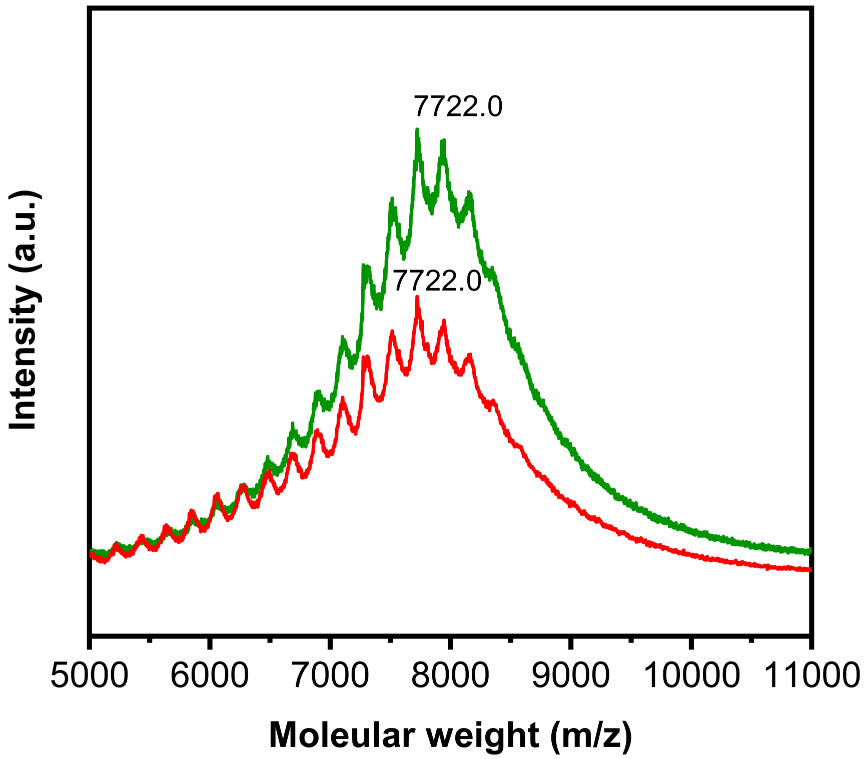

Figure S11 MALDI-TOF spectra of COOH-RhMOP before (green) and after (red) exposing the MOP to the conditions used for the synthesis of RhCu-btc-HKUST-1. The weight corresponding to the formula [COOH-RhMOP + 3 DMF + 2 H_2O + H^+] has been highlighted: expected = 7721.162; found = 7722.0.

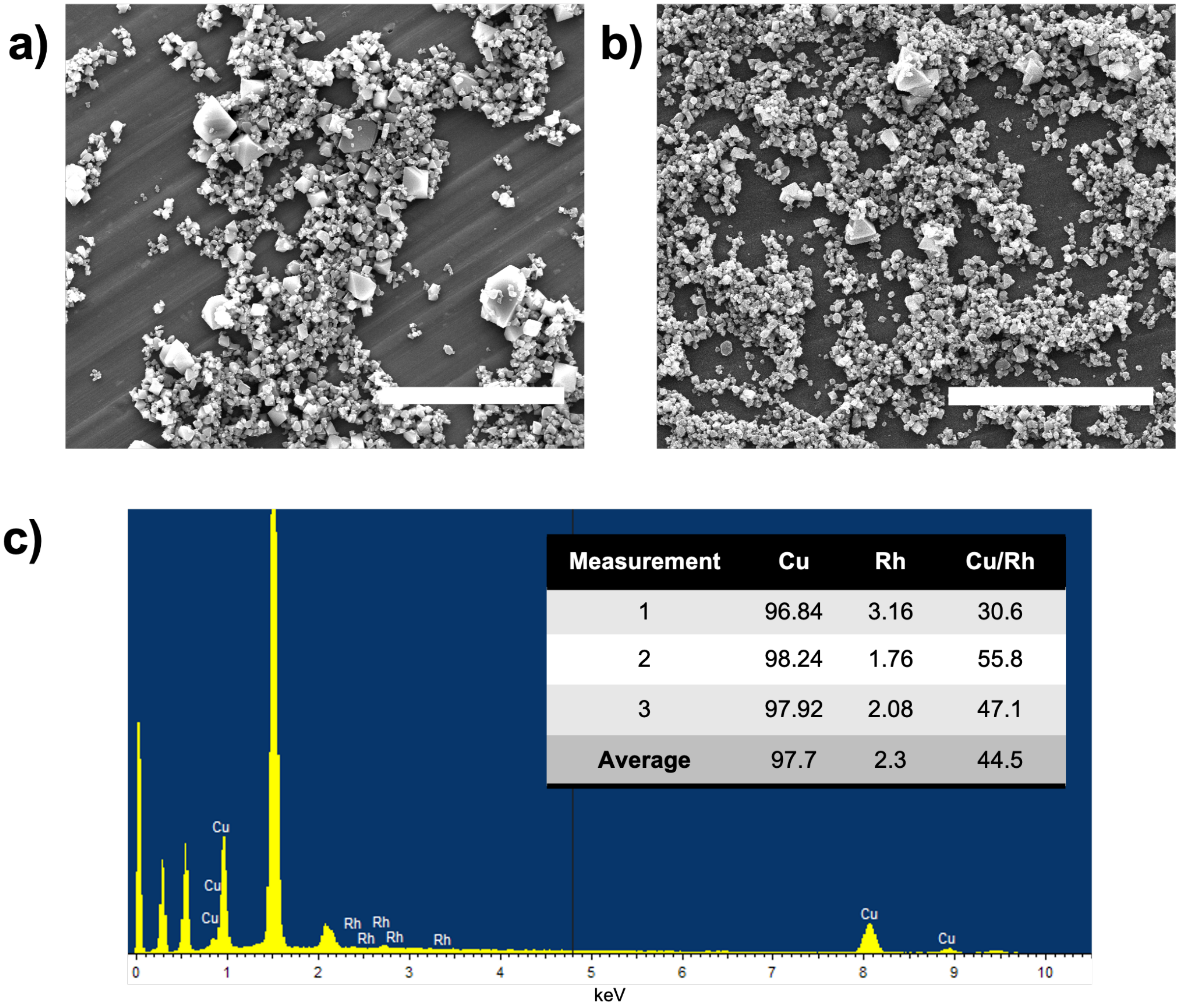

Measurement	Cu	Rh	Cu/Rh
1	96.84	3.16	30.6
2	98.24	1.76	55.8
3	97.92	2.08	47.1
**Average**	97.7	2.3	44.5

Figure S12 a) FESEM image of initial Cu(II)-HKUST-1 crystals before exposing them to a solution containing COOH-RhMOP. (b) FESEM image of Cu(II)-HKUST-1 crystals after exposing them to a solution containing COOH-RhMOP. Scale bar: 50 μm (a, b). (c) EDX spectrum obtained from Cu(II)-HKUST-1 crystals after exposing them to a solution containing COOH-RhMOP. The inset shows the atomic percentage of Rh(II) and Cu(II) ions in the sample, which show that the post-incubated Cu(II)-HKUST-1 contain a residual percentage of Rh(II) ions.

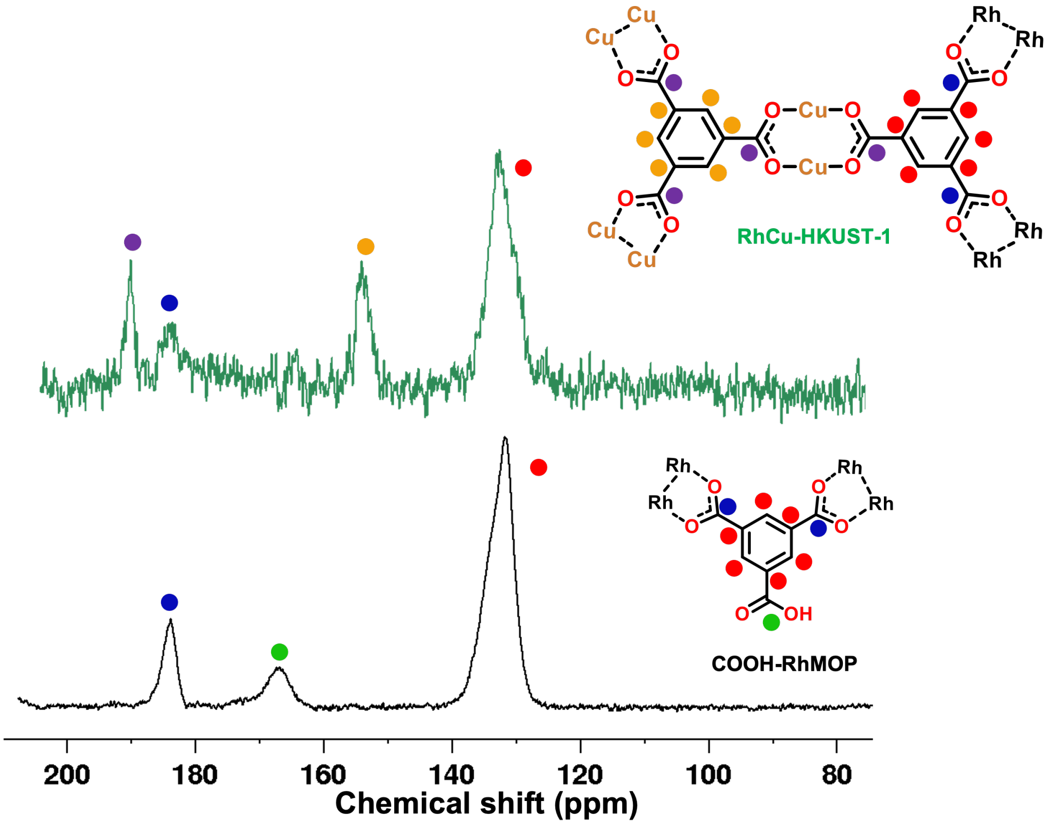

Figure S13 Solid-state (CP/MAS) ^{13}C NMR spectra (400 MHz) of COOH-RhMOP (black) and RhCu-btc-HKUST-1 (green). Note that RhCu-btc-HKUST-1 is constructed out from COOH-RhMOP (blue and red dots), and additional btc linkers (purple and orange dots). No peak attributed to the free carboxylic group of COOH-RhMOP (green dot) is observed, further suggesting that all COOH groups are coordinated to Cu(II) ions.

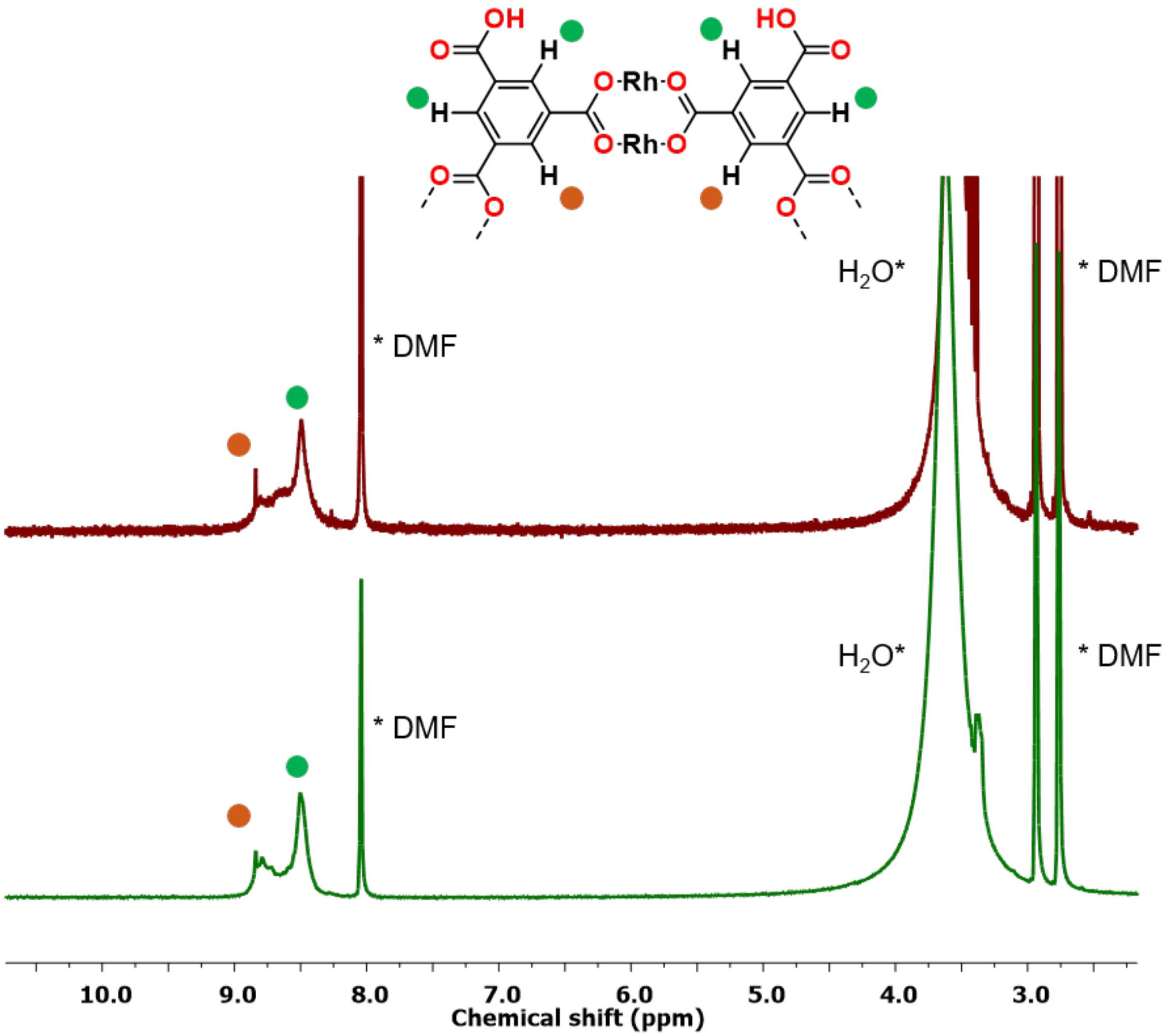

Figure S14 ^{1}H NMR spectra (400 MHz, DMF-d_7) of the initial COOH-RhMOP (green) and the COOH-RhMOP isolated from RhCu-btc-HKUST-1 through acidic disassembly (wine). Note that COOH-RhMOP maintains its integrity after the disassembly of RhCu-btc-HKUST-1 under slightly acid conditions.

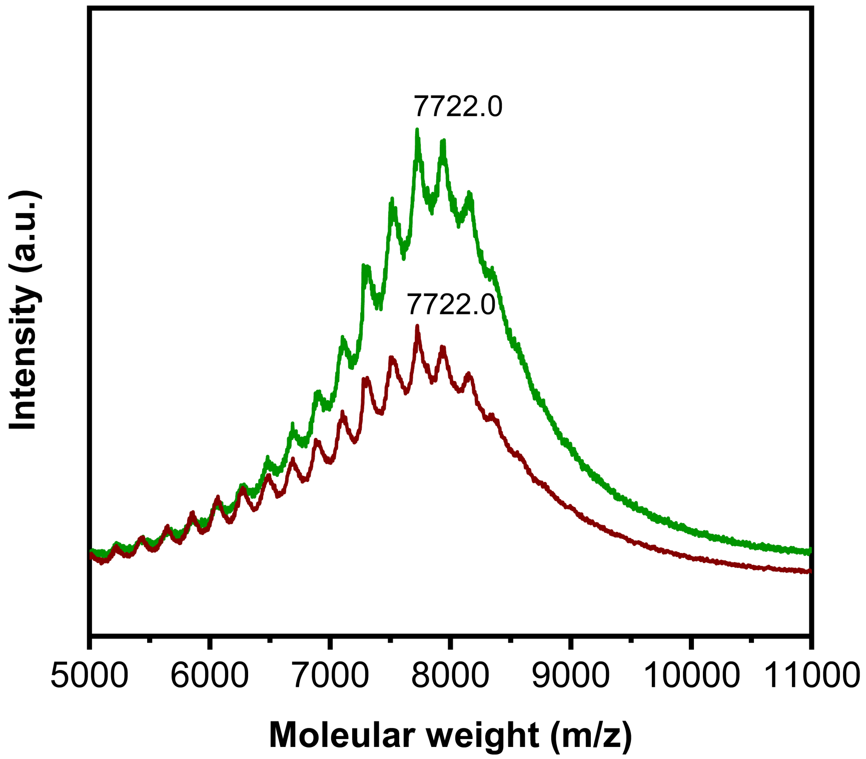

Figure S15 MALDI-TOF spectra of COOH-RhMOP (green) and the product (wine) resulting from the disassembly of RhCu-btc-HKUST-1. The weight corresponding to the formula [COOH-RhMOP + 3 DMF + 2 H_2O + H^+] has been highlighted: expected = 7721.2; found = 7722.0.

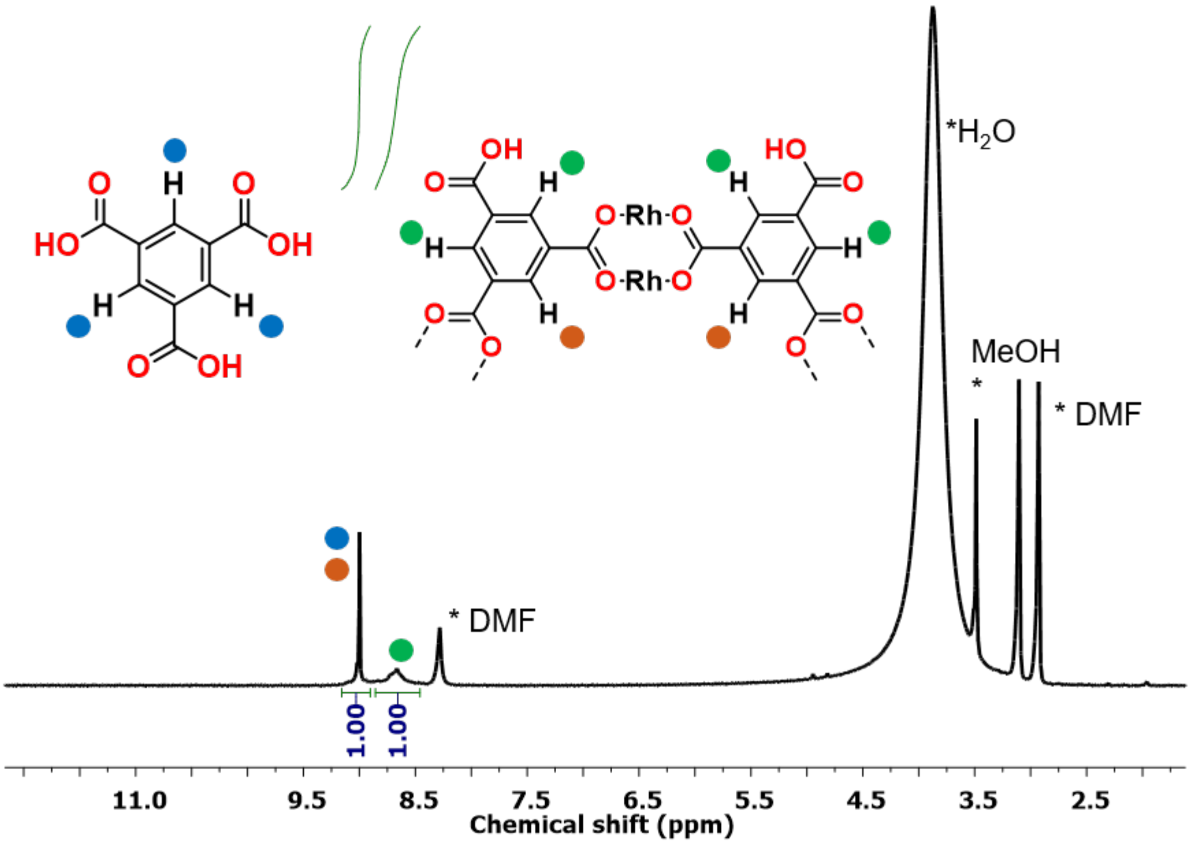

Figure S16 ^{1}H NMR spectra (360 MHz, DMF-d_7) of the solution obtained after the acidic disassembly of RhCu-btc-HKUST-1 into COOH-RhMOP and btc. Note that the connectivity of RhCu-btc-HKUST-1 defines a ratio COOH-RhMOP:btc of 1:8. Accordingly, the sum of the three btc protons (blue dot, 8X3 = 24H) plus the inner proton of COOH-RhMOP (orange dot, 24 ligands per MOP with 1 inner proton each 1H each = 24H) should integrate the same as all the external protons of the COOH-RhMOP (green dots, 24 ligands per MOP with 2 outer protons each = 24*2 =48H). Therefore, the 1:1 ratio between the two set of signals confirms that the stoichiometry between COOH-RhMOP and btc is 1:8.

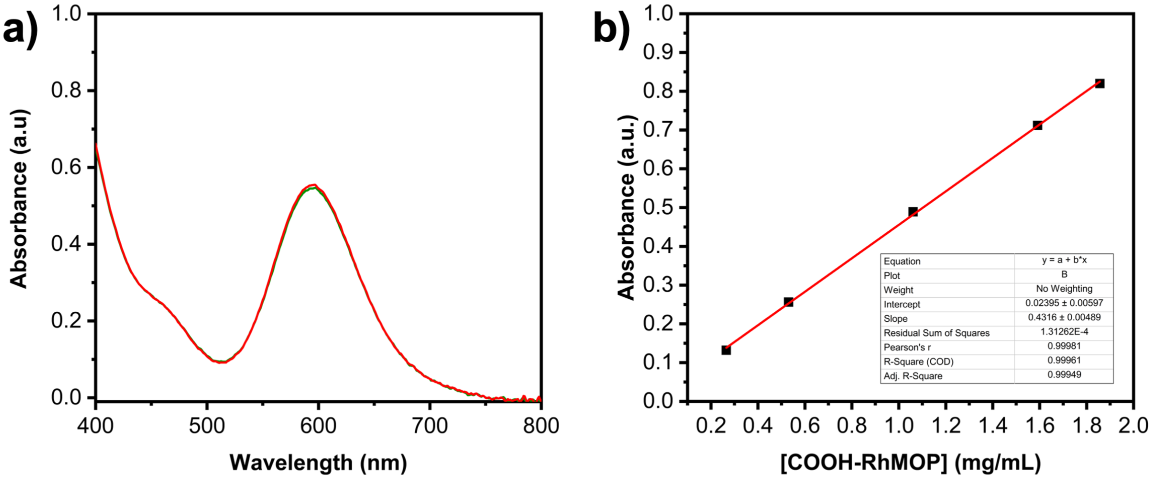

Figure S17 COOH-RhMOP quantification after disassembly of RhCu-btc-HKUST-1. (a) UV-Vis spectra of COOH-RhMOP recovered after disassembly of RhCu-btc-HKUST-1. Green and red spectra correspond to two replicates. (b) Calibration curve displaying the linear correlation between absorbance and MOP concentration. The fitting equation and the correlation factor are also shown (inset).

Table S2 UV-Vis adsorption data used to determine the amount of extracted COOH-RhMOP from RhCu-btc-HKUST-1.

RhCu-btc-HKUST-1 (mg)	Abs. Of extracted COOH-RhMOP	Dilution factor	Concentration of COOH-RhMOP (mg/mL)	Amount of extracted COOH-RhMOP (mmol/mg)	Theoretical amount of COOH-RhMOP in RhCu-btc-HKUST-1 (mmol/mg)	Recovery efficiency (%)
**17.4**	0.545	1/10	1.21	$9.25 \cdot 10^{-5}$	$9.42 \cdot 10^{-5}$	98.2
**17.4**	0.555	1/10	1.23	$9.40 \cdot 10^{-5}$		99.9

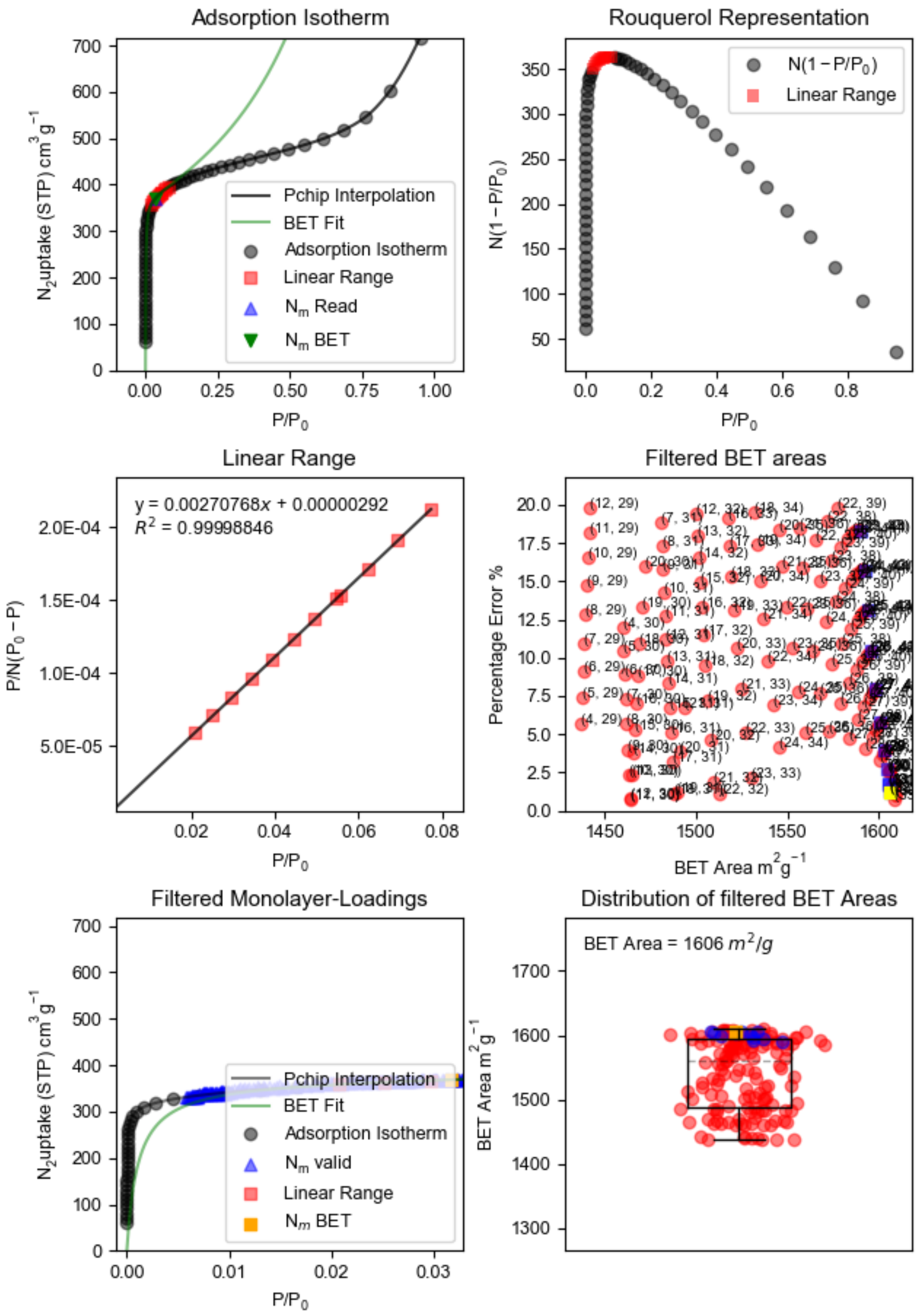

Figure S18 BETSI analysis of RhCu-btc-HKUST-1.

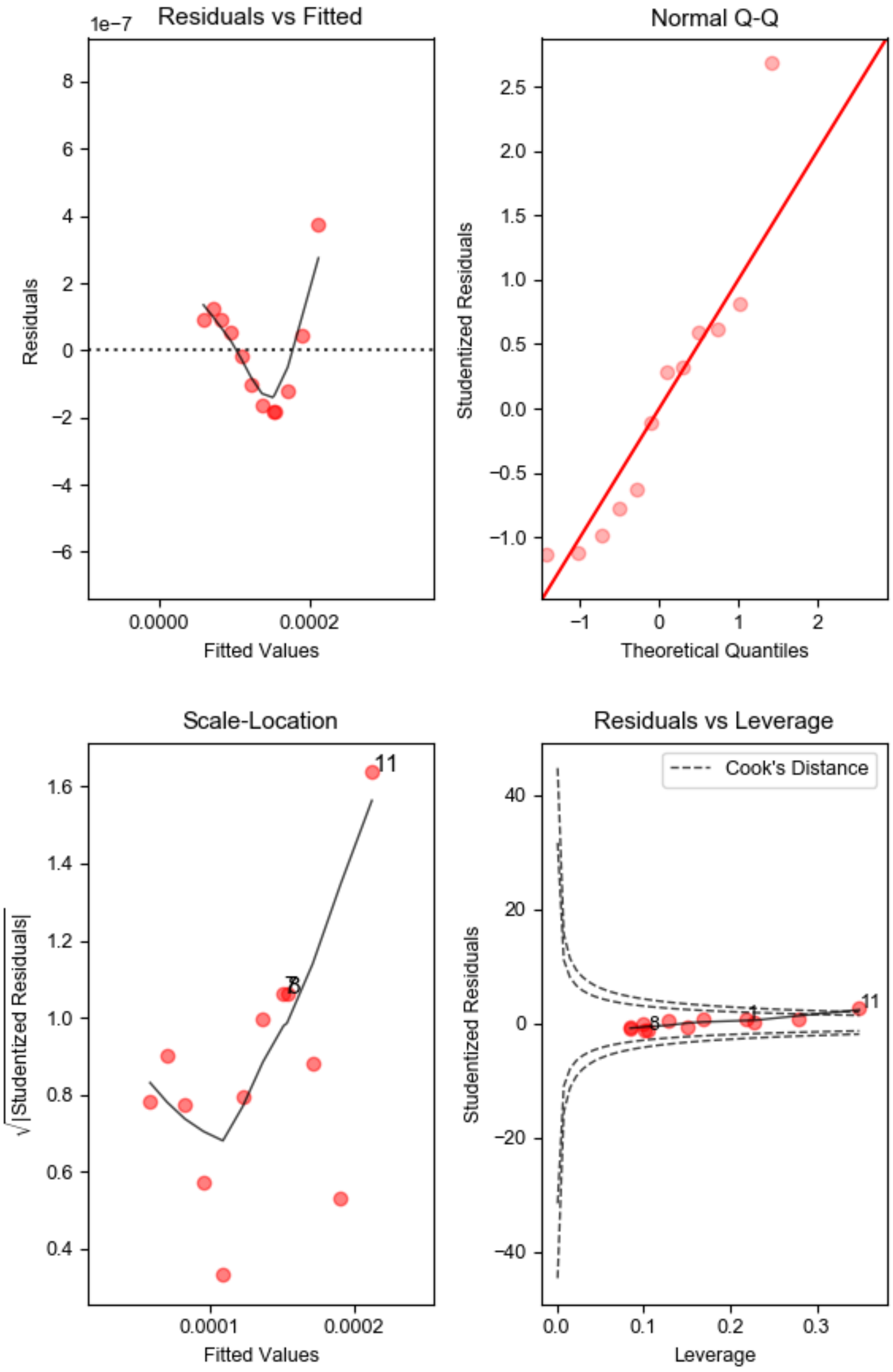

Figure S19 BETSI regression diagnostics for RhCu-btc-HKUST-1.

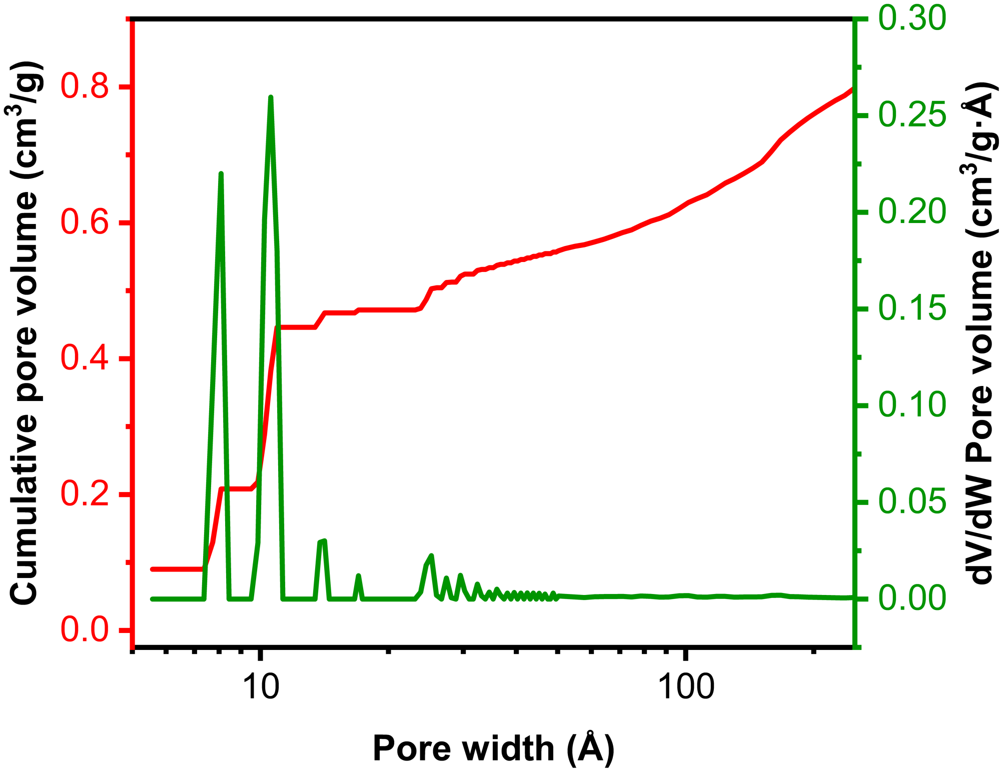

Figure S20 Pore size distribution of RhCu-btc-HKUST-1. Note that the first three peaks correspond to the three characteristic cavities of the Cu-HKUST-1 structure: 5 Å, 11 Å and 15 Å.[19] In addition, mesoporosity ascribed to the inter-particle voids was also observed.

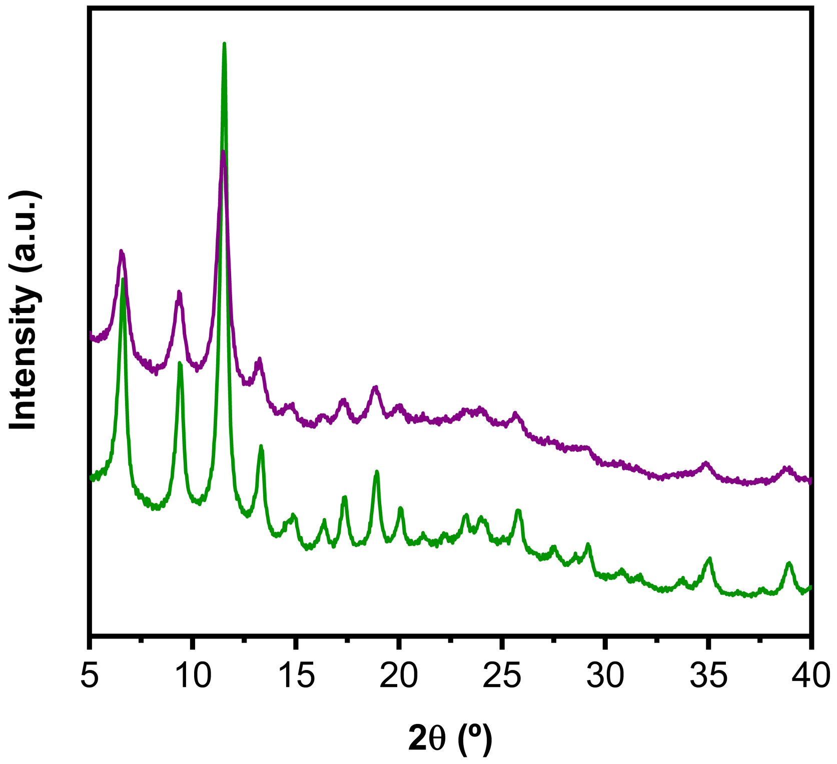

Figure S21 PXRD of the as-made RhCu-btc-HKUST-1 (green) and activated RhCu-btc-HKUST-1 (purple).

S3. Characterization of Cu(II)-HKUST-1.

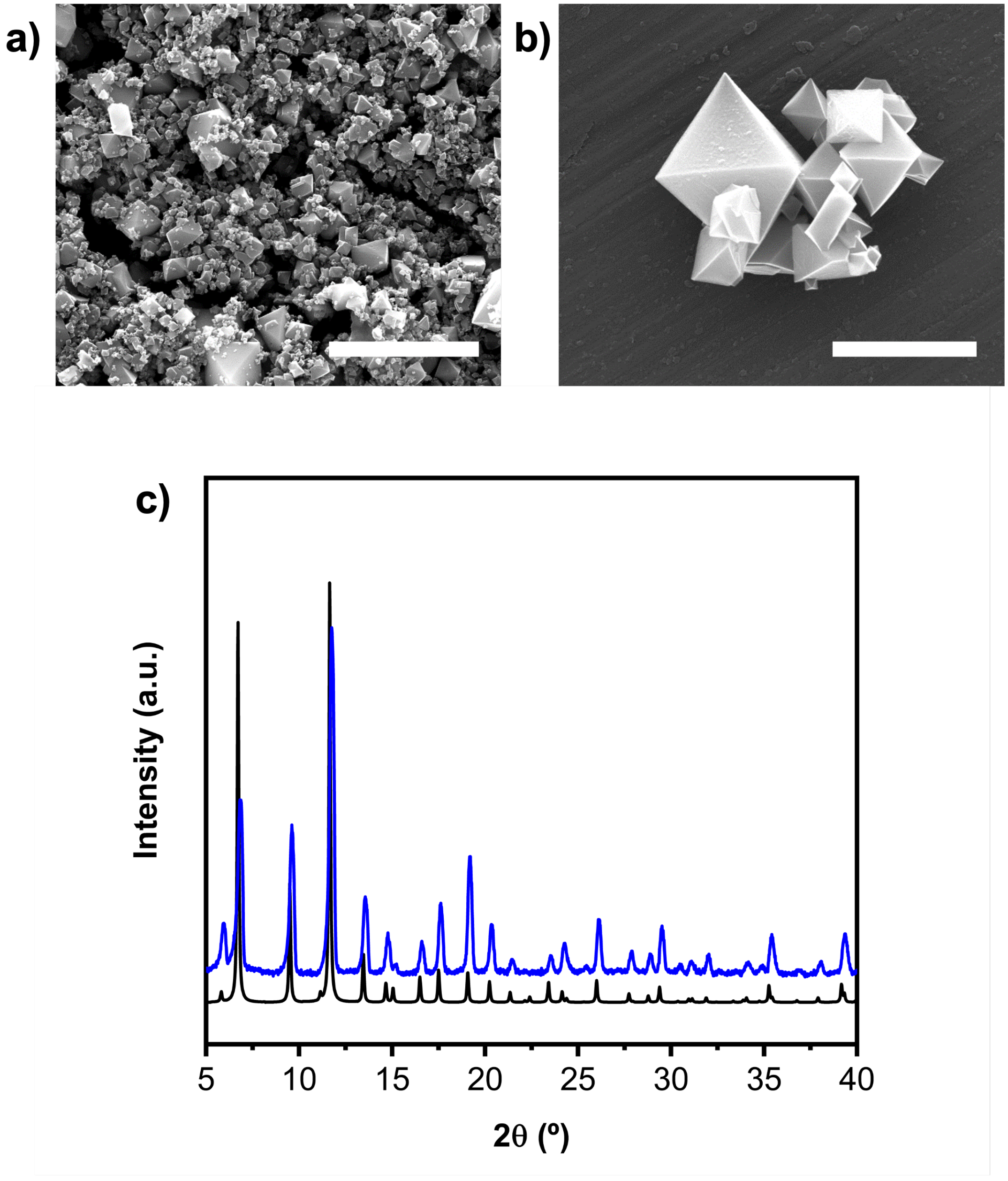

Figure S22 (a,b) FESEM images of Cu(II)-HKUST-1 obtained under the same experimental conditions used for RhCu-btc-HKUST-1 except for the absence of COOH-RhMOP. Note that the octahedral crystals are significantly larger than the RhCu-btc-HKUST-1, which highlights the strong nucleation effect of COOH-RhMOP in the reaction. Furthermore, the absence of such large crystals in the synthesis of RhCu-btc-HKUST-1 further evidence that the formation of pure Cu(II)-HKUST-1 is suppressed in the presence of COOH-RhMOP. Scale bars: 10 µm (a), and 2 µm (b). (c) Simulated PXRD of Cu(II)-HKUST-1 (black) and experimental PXRD of Cu(II)-HKUST-1 (blue).

S4. Hydrolytic stability study of RhCu-btc-HKUST-1 and Cu(II)-HKUST-1.

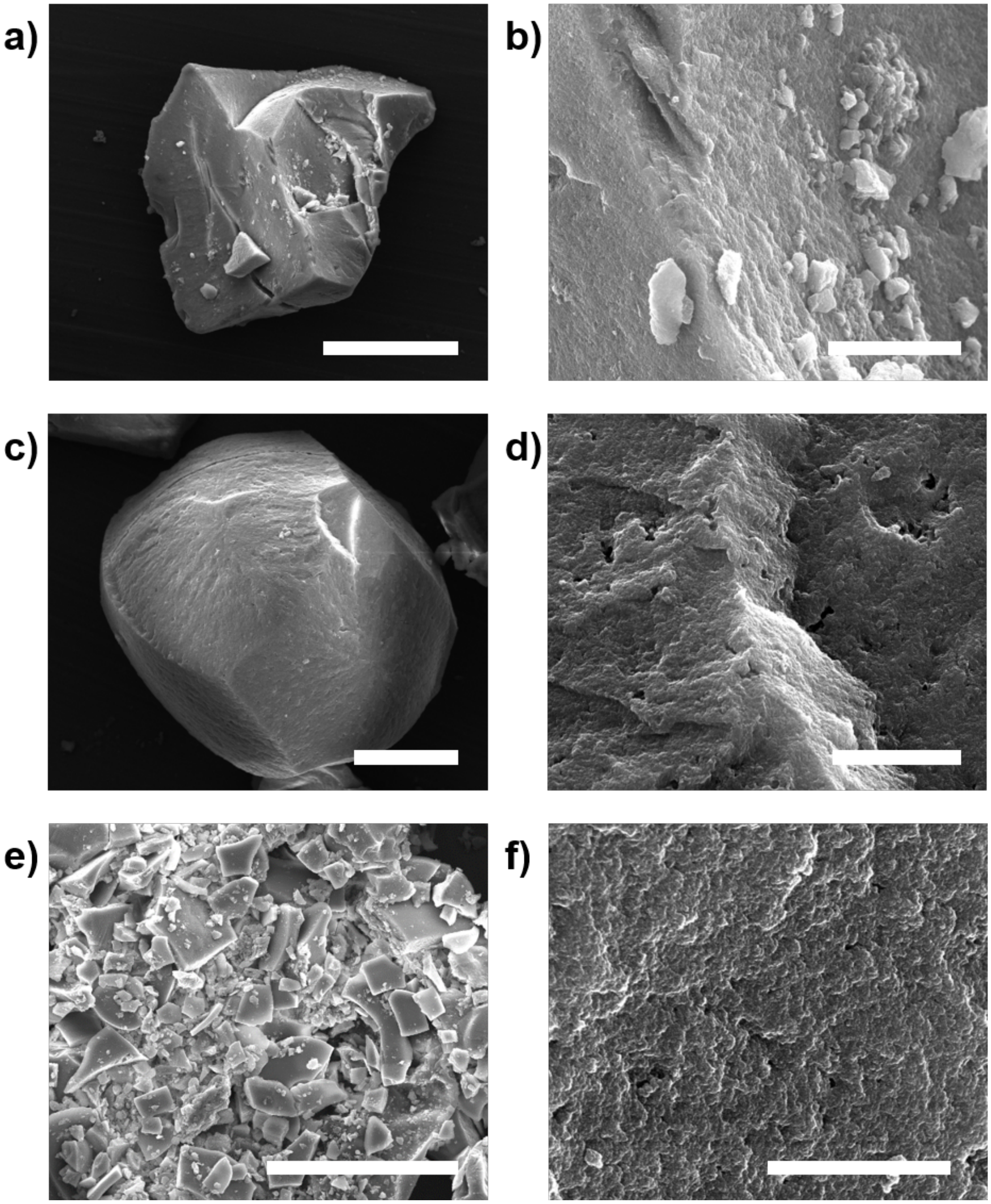

Figure S23 FESEM images (a, b) of as-made RhCu-btc-HKUST-1, (c, d) after incubation it in water for 24 h, and (e, f) after 31 days of incubation in water, showing the characteristic monolithic aggregates of RhCu-btc-HKUST-1 nanoparticles. Note that RhCu-btc-HKUST-1 does not undergo any morphological change even after 31 days in water, and that the characteristic rod-like crystals (see Figure S24) observed in the hydrolytic degradation of the parent Cu(II)-HKUST-1 are not observed. These observations further confirm the absence of pure Cu(II)-HKUST-1 dominions in the RhCu-btc-HKUST-1 sample. Scale bars: 50 μm (a, c), 5 μm (b, d),100 μm (e) and 2 μm (f).

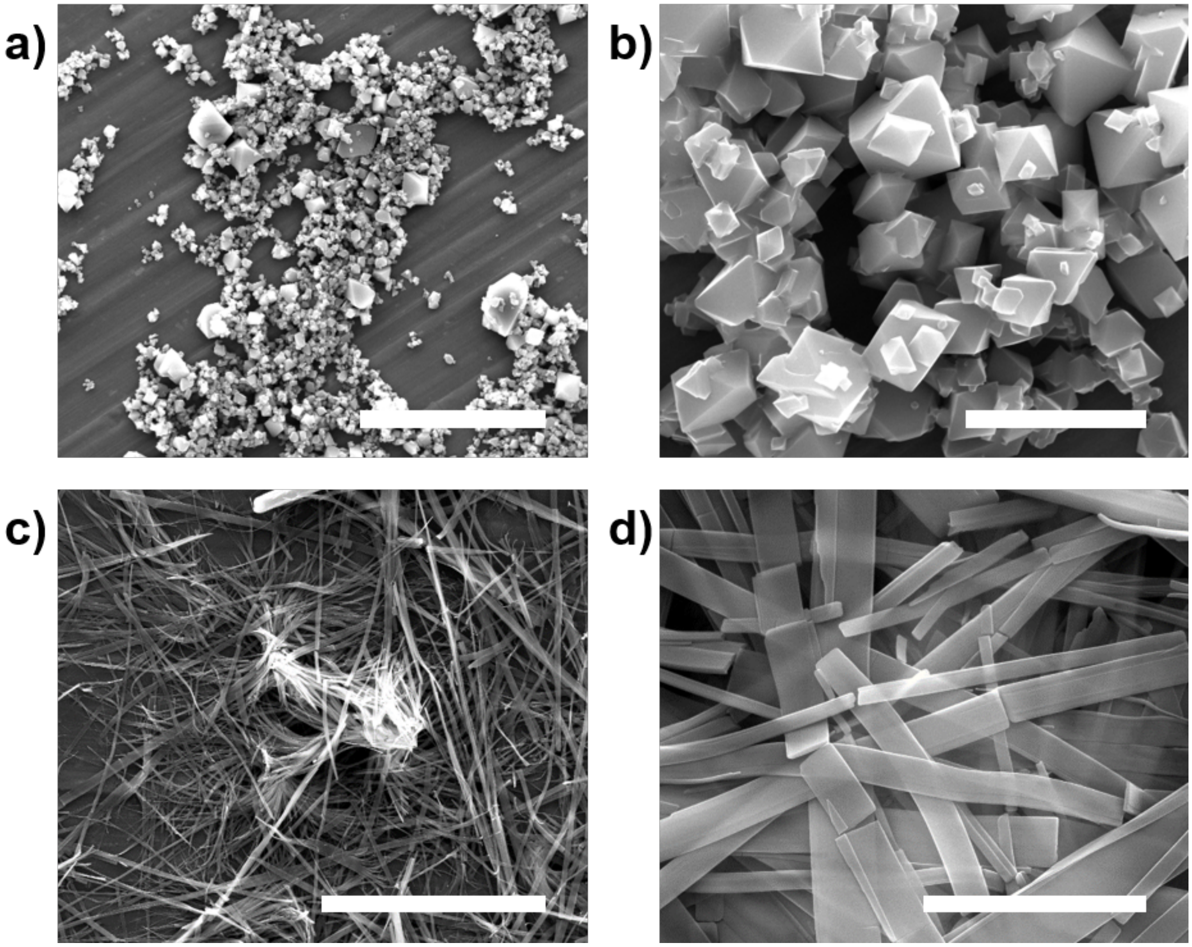

Figure S24 (a, b) FESEM images of as-made Cu(II)-HKUST-1 and (c, d) after incubation in water for 24 h, which show the reported conversion of the octahedral Cu(II)-HKUST-1 crystals into rod-like crystals upon water incubation.[20] Scale bars: (a, c) 50 μm and (b, d) 5 μm.

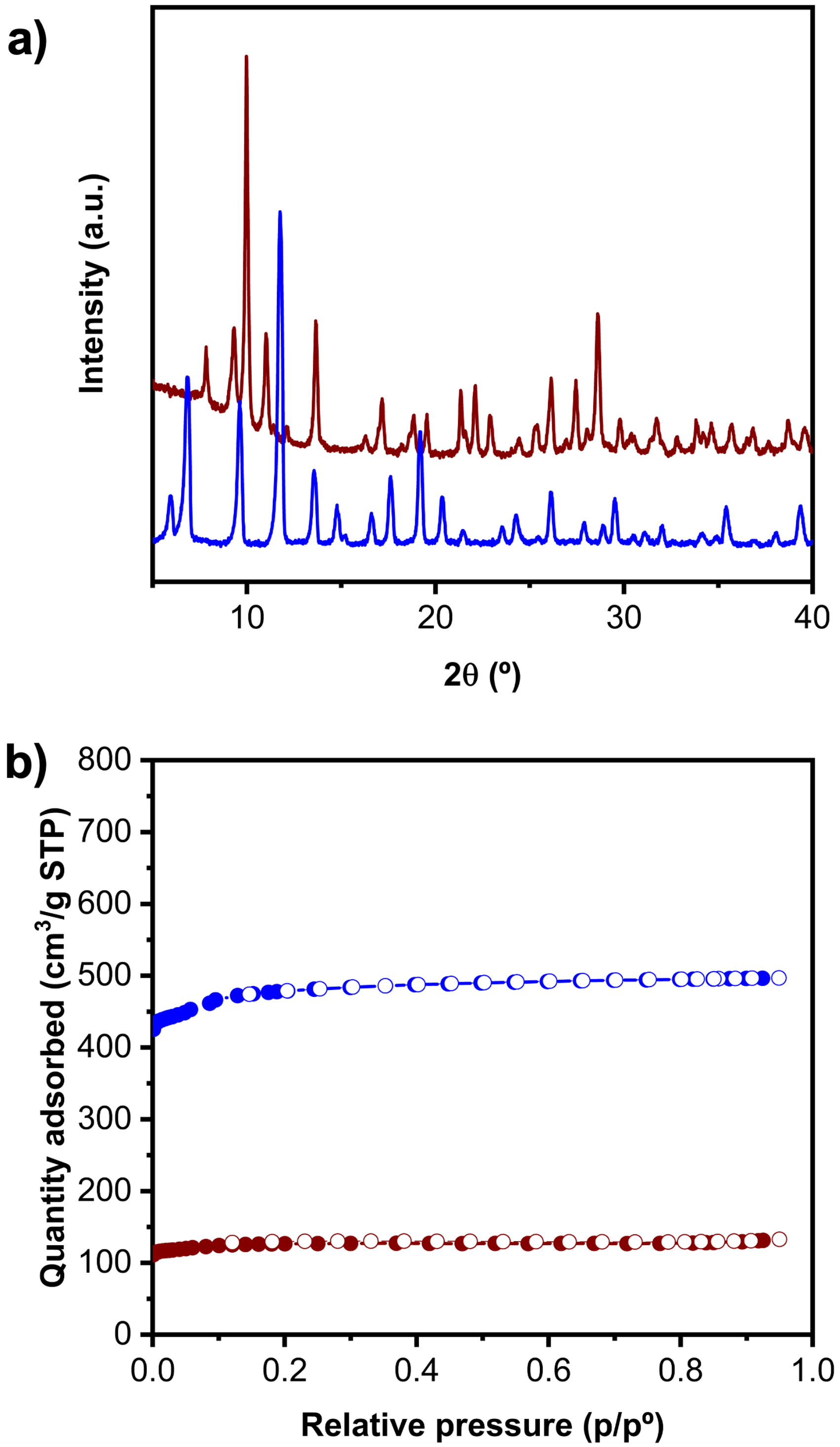

Figure S25 (a) PXRD of the as-made Cu(II)-HKUST-1 (blue) and the one obtained after incubating it for 24 h in water (wine). The reported water-induced structural transformation of Cu(II)-HKUST-1 was observed.[20] (b) N_2 adsorption isotherms of the as-made Cu(II)-HKUST-1 (blue) and the Cu(II)-HKUST-1 incubated in water for 24 h (wine). Note that the as-made Cu(II)-HKUST-1 showed to be microporous to N_2 with a S_{BET} of 1888 m^2/g. However, after incubation in water for 24 h, the S_{BET} was reduced to 502 m^2/g, in agreement with previously reported results.[20,21]

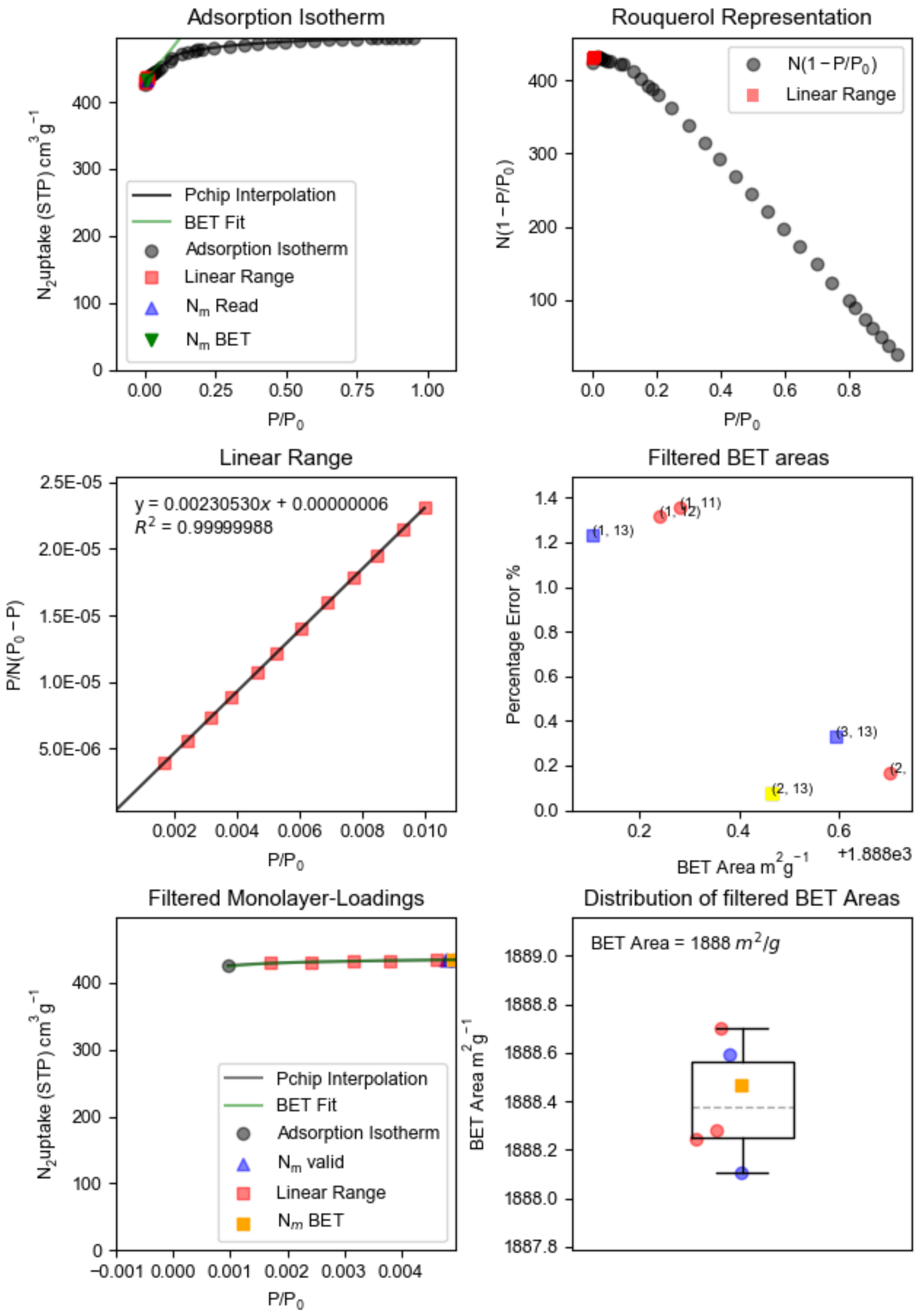

Figure S26 BETSI analysis of Cu(II)-HKUST-1.

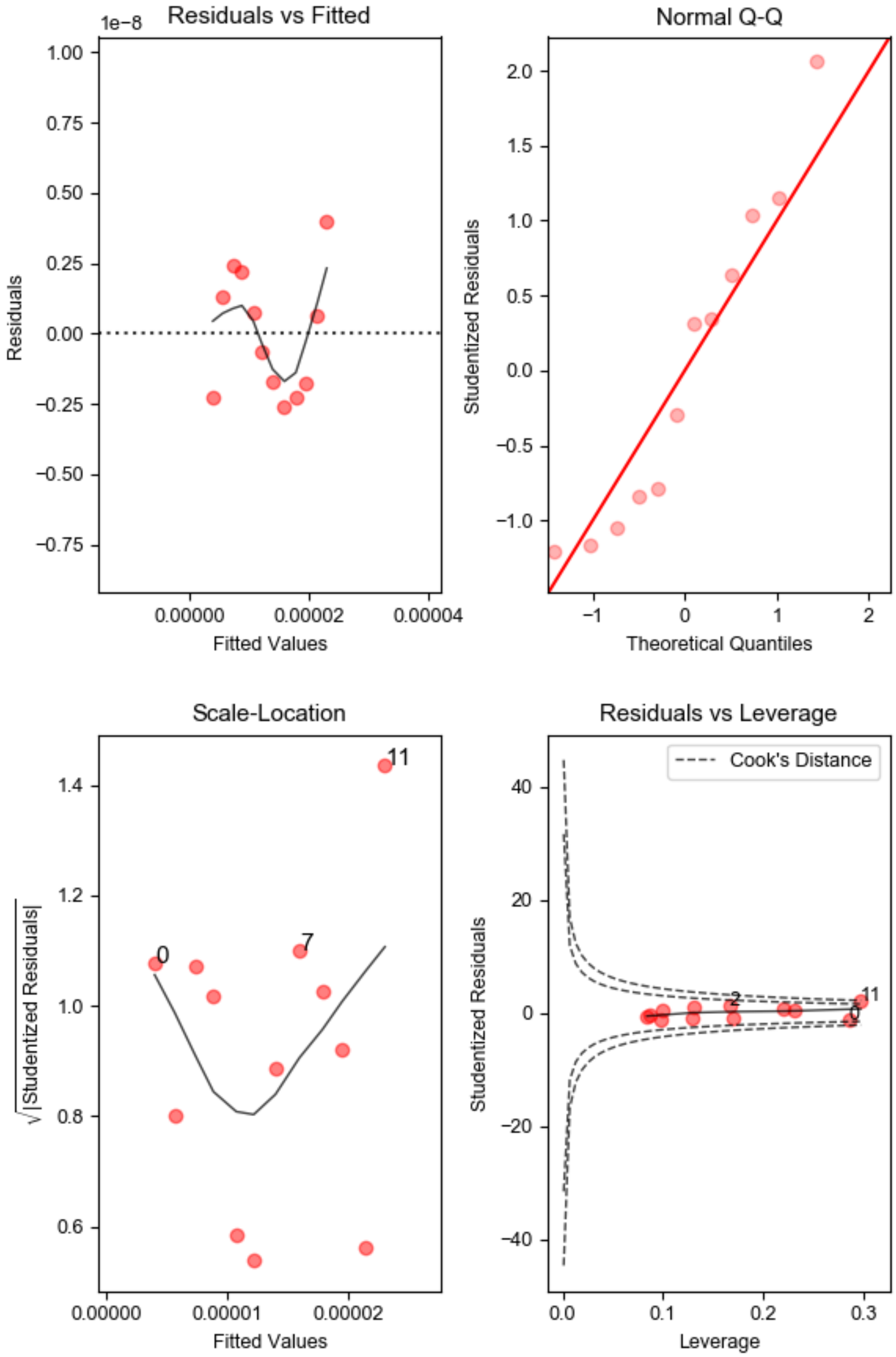

Figure S27 BETSI regression diagnostics for Cu(II)-HKUST-1.

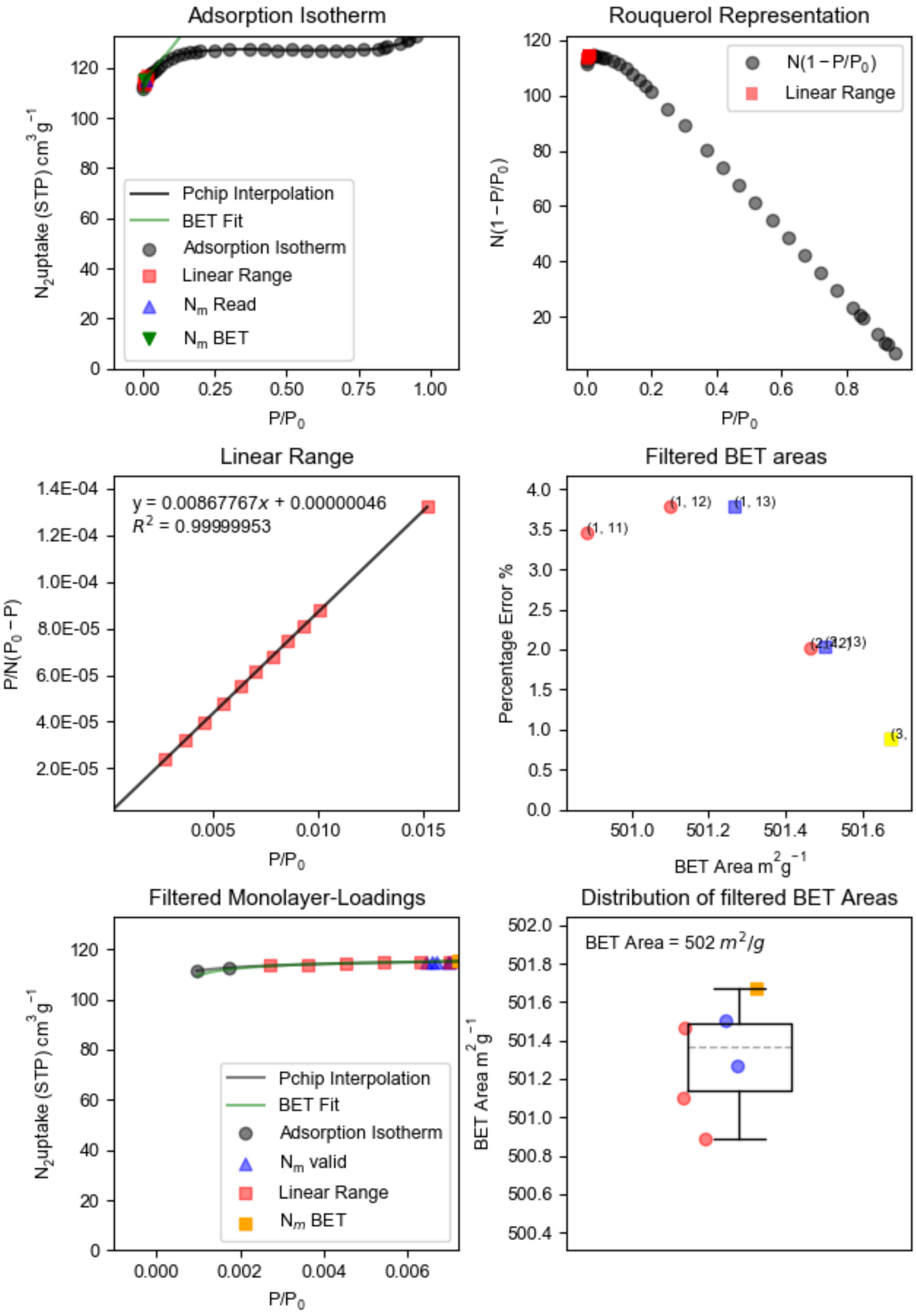

Figure S28 BETSI analysis for Cu(II)-HKUST-1 after incubation in water for 24 h.

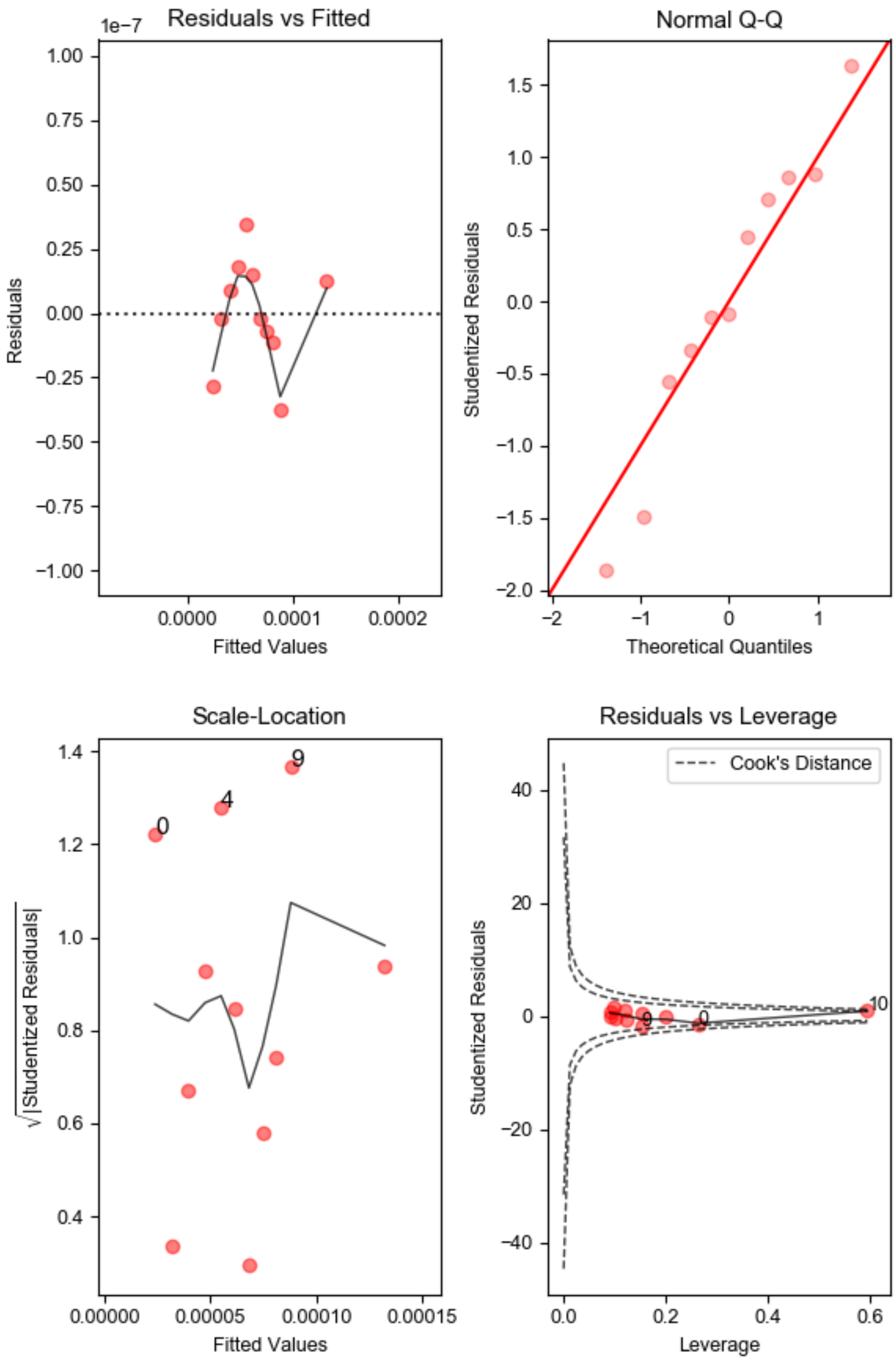

FigureS29 BETSI regression diagnostics for Cu(II)-HKUST-1 after incubation in water for 24 h.

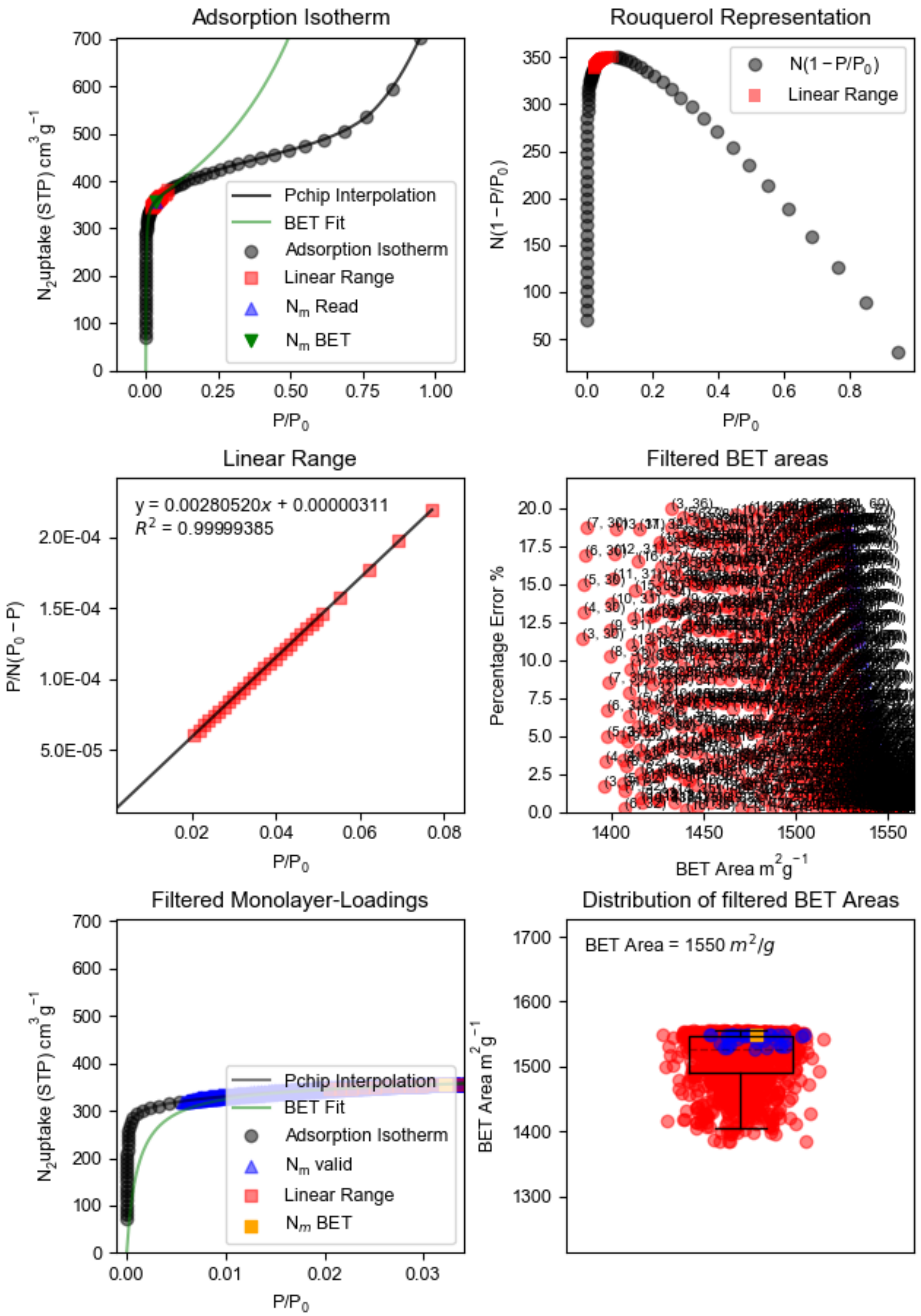

FigureS30 BETSI analysis for RhCu-btc-HKUST-1 after incubation in water for 3 days.

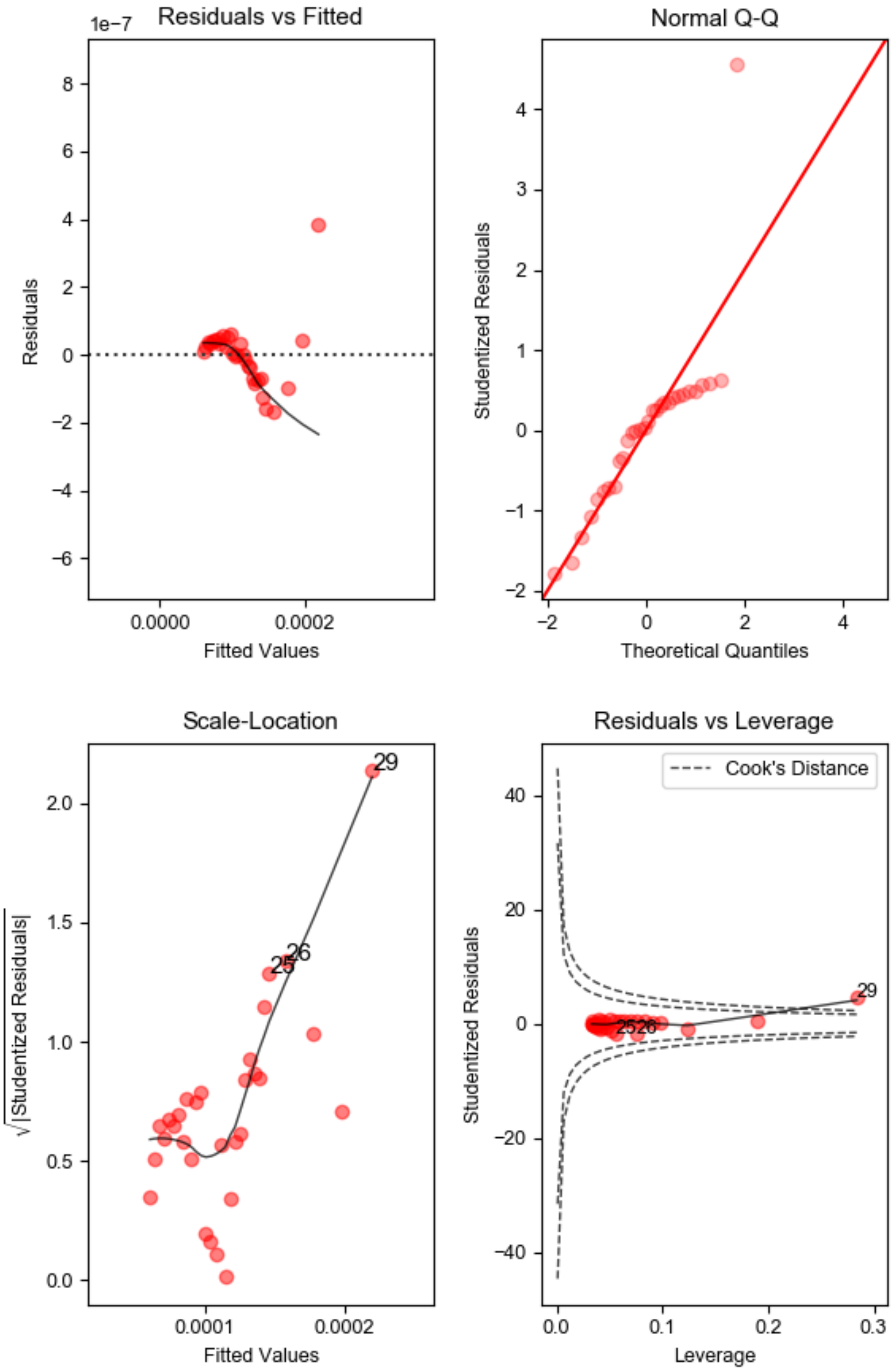

Figure S31 BETSI regression diagnostics of RhCu-btc-HKUST-1 after incubation in water for 3 days.

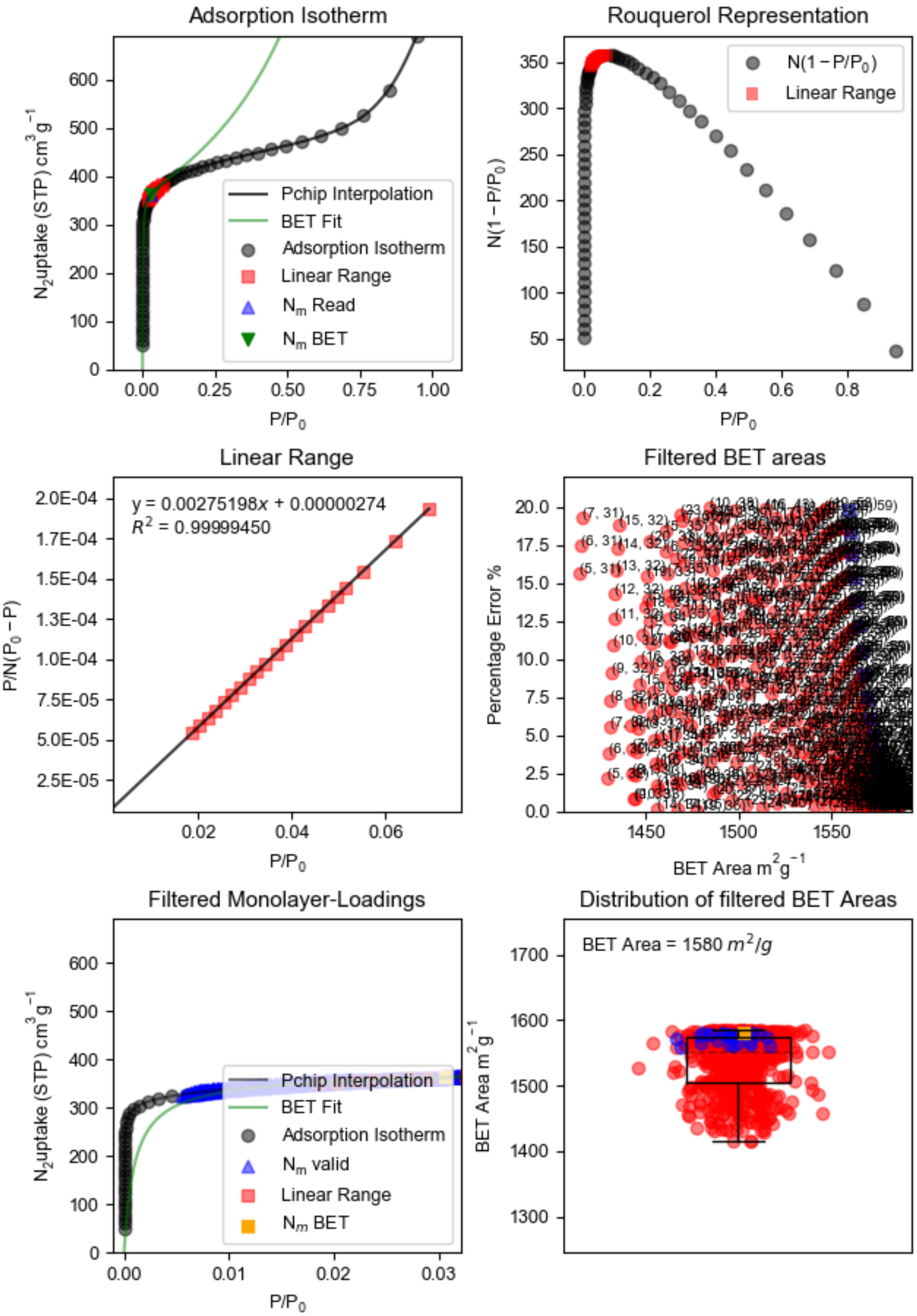

Figure S32 BETSI analysis of RhCu-btc-HKUST-1 after incubation in water for 14 days.

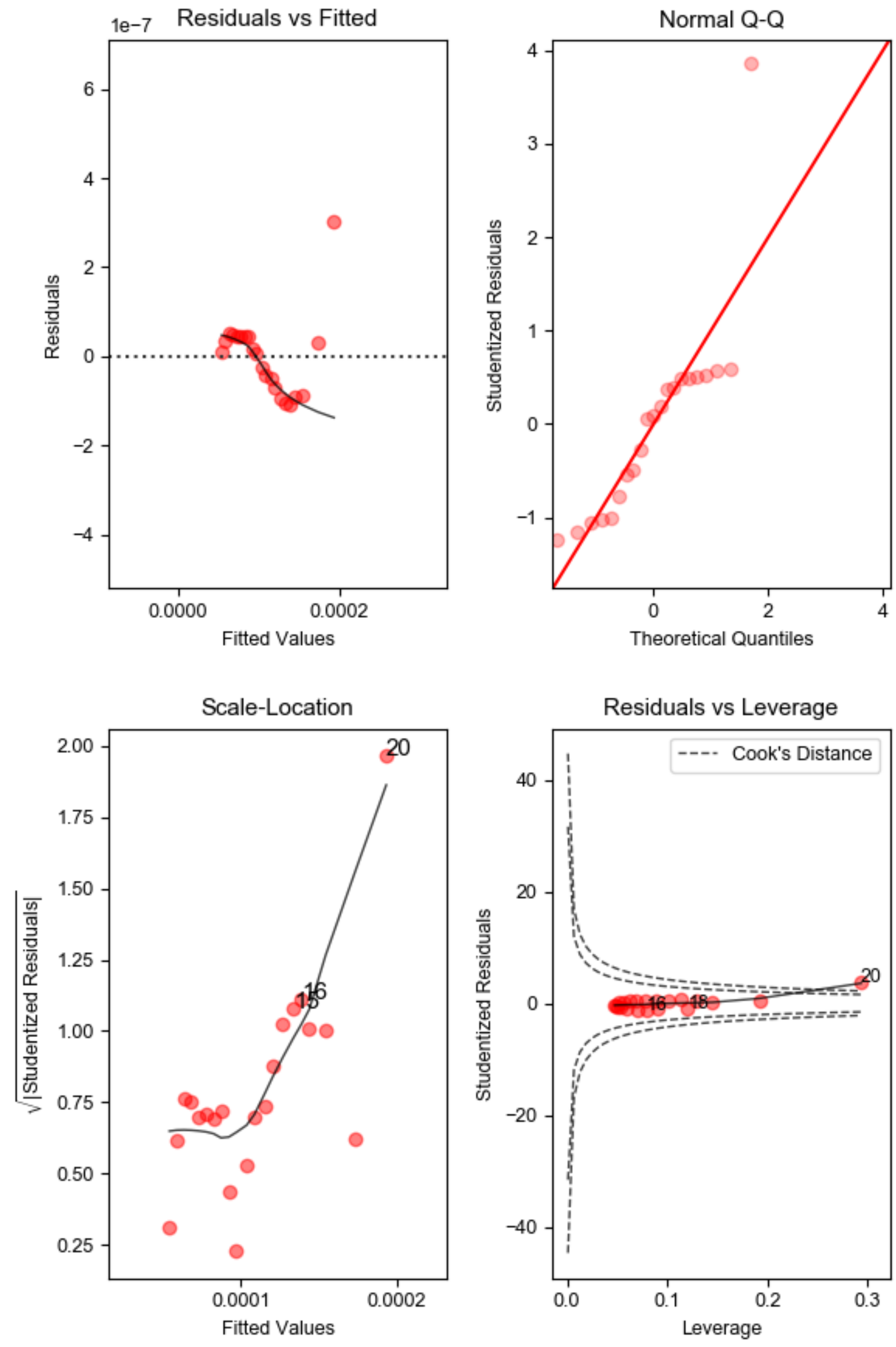

Figure S33 BETSI regression diagnostics of RhCu-btc-HKUST-1 after incubation in water for 14 days.

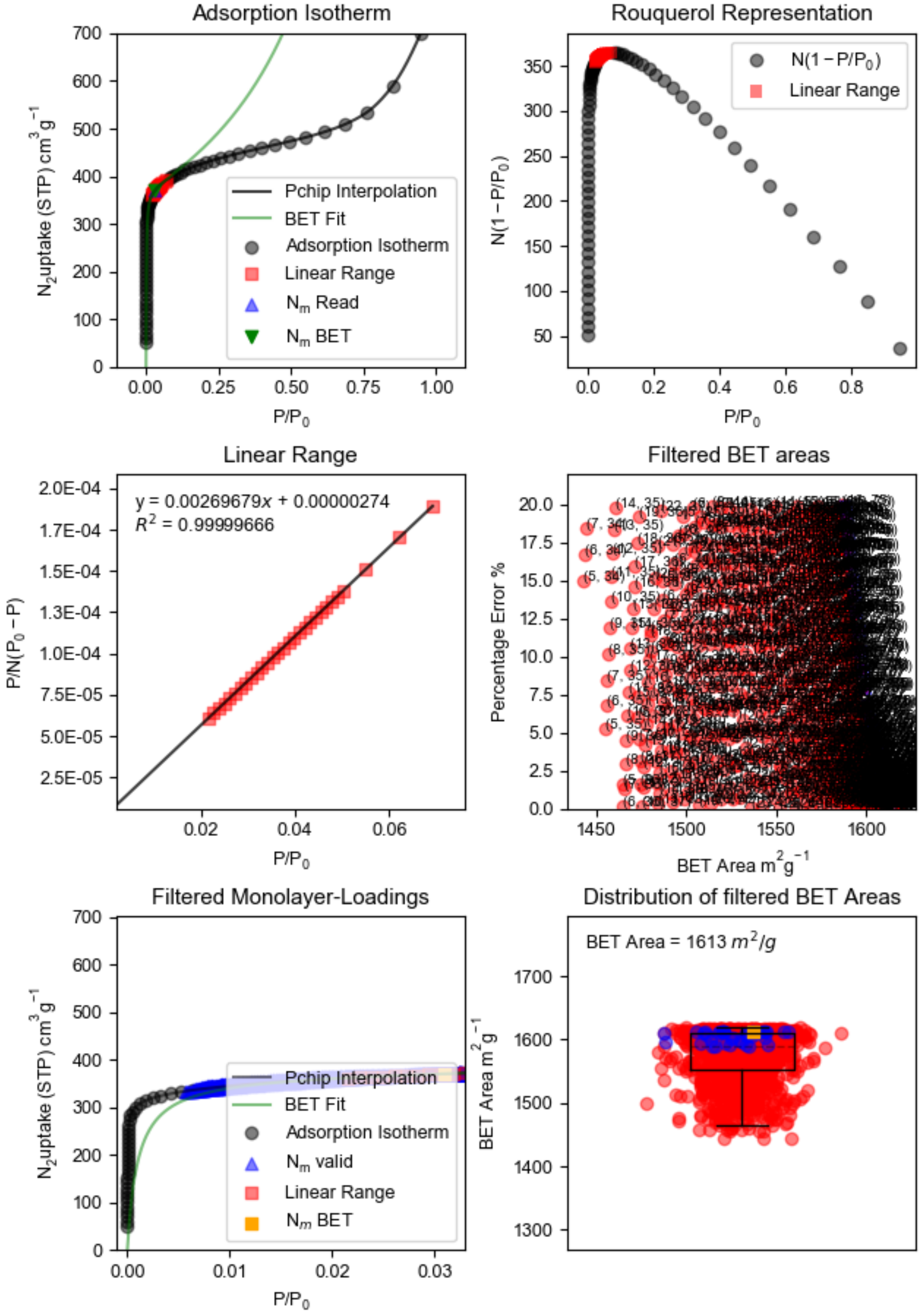

Figure S34 BETSI analysis of RhCu-btc-HKUST-1 after incubation in water for 31 days.

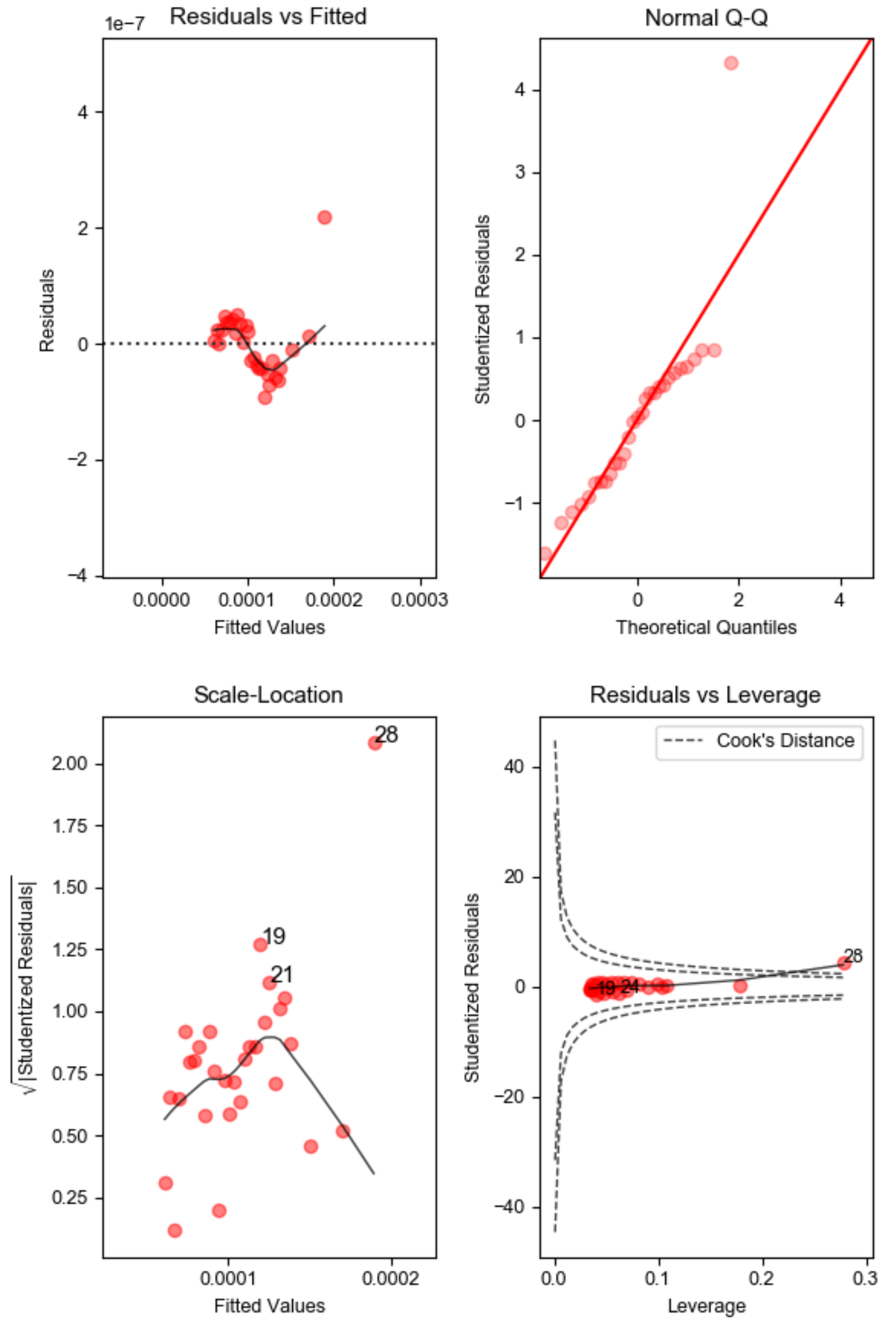

Figure S35 BETSI regression diagnostics for RhCu-btc-HKUST-1 after incubation in water for 31 days.

S4.1 DFT calculations of Rh(II) and Cu(II) paddlewheels in water

The results obtained from the first set of calculations (metal paddlewheel in implicit water) are shown in Figures S36–S38. The results obtained in the second set of calculations (effect of explicit water molecules coordinating at the axial metal positions) are shown in Figures S39–S41.

In Figures S36–S38, we show the geometrical features of the DFT optimized structures of metal acetate paddlewheels in implicit water. In the case of the Rh(II) paddlewheel, all Rh-O(acetate) distances are identical at 2.07 Å forming a symmetrical paddlewheel (Figure S36a). In the case of the Cu(II) paddlewheel, all Cu-O(acetate) distances are identical (~1.97 Å) but the structure shows torsion, leading to a distorted paddlewheel (Figure S36b).

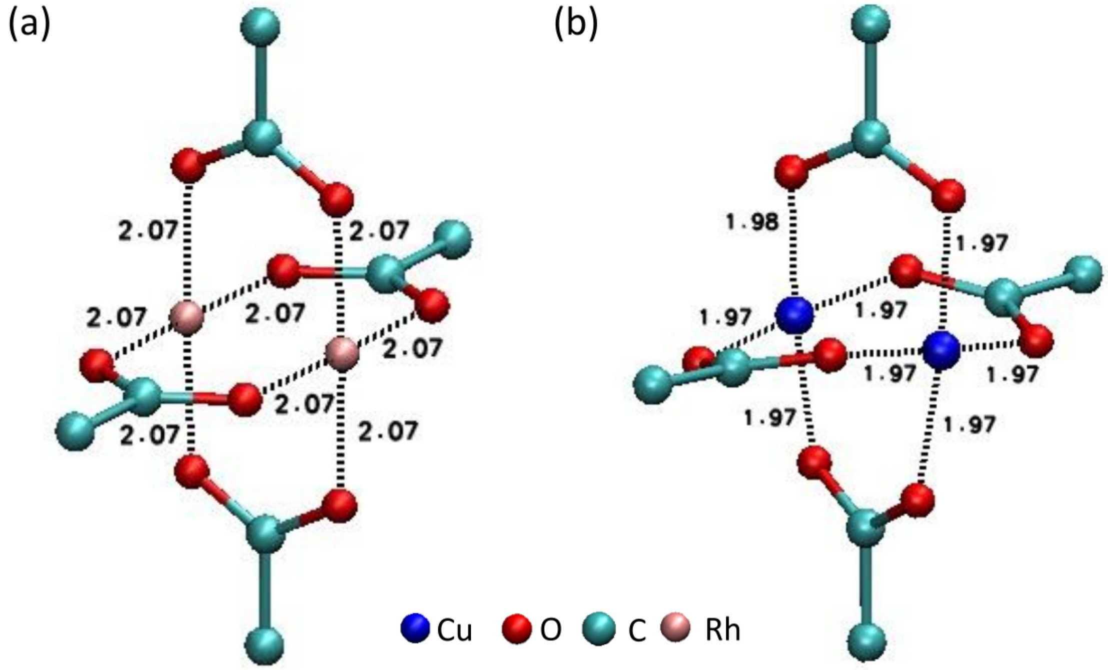

Figure S36. Results of DFT optimization of metal acetate paddlewheel structures (first simulation set). DFT Optimized metal acetate paddlewheel structures in implicit water with indication of the bond distances (in Å) between the metal and oxygen atoms. (a) Rhodium acetate paddlewheel and (b) Copper acetate paddlewheel.

The structural differences between the Cu(II) and Rh(II) paddlewheel structures simulated in implicit water can be better appreciated by comparing their respective angles and dihedral angles as depicted in Figures S37 and S38. In the case of the Rh(II) cluster, all Rh-O-O angles shown in the figure S37 are ~91.5º and the dihedrals defined by oxygen atoms of opposite acetates are negligible, confirming that the paddlewheel is symmetrical. In the case of the Cu(II) cluster, the Cu-O-O angle is ~92.5º and dihedral angle defined by oxygen atoms of opposite acetates is ~25º.

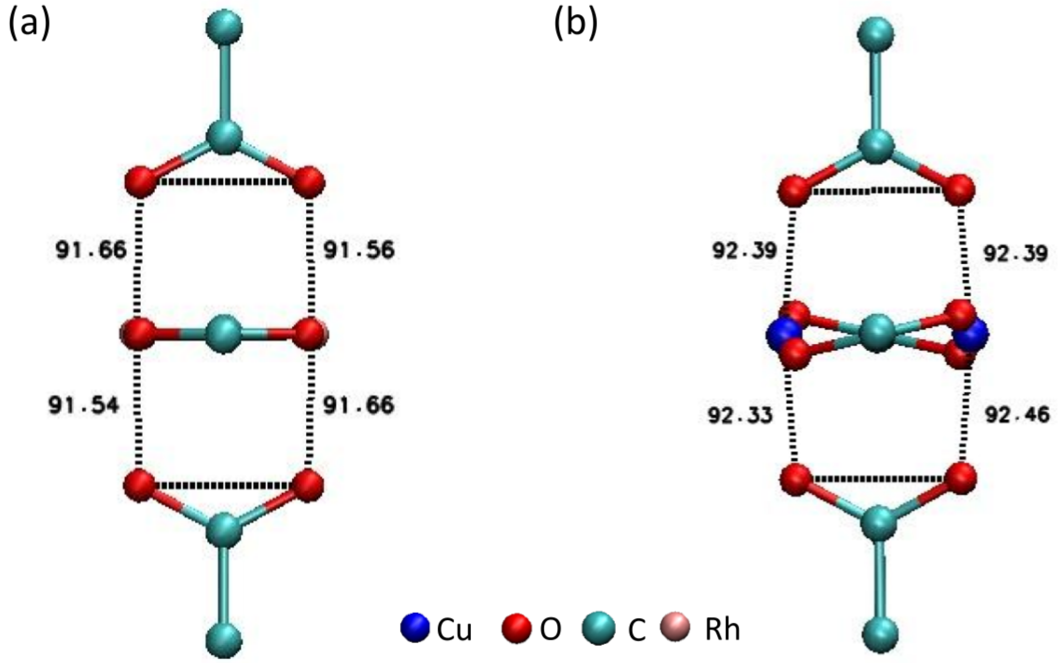

Figure S37. Selected angles (in degrees) for the structures reported in Figure S36. Selected metal-O-O angles for the DFT optimized paddlewheel structure in implicit water shown in Figure S36. (a) Rhodium acetate paddlewheel and (b) Copper acetate paddlewheel.

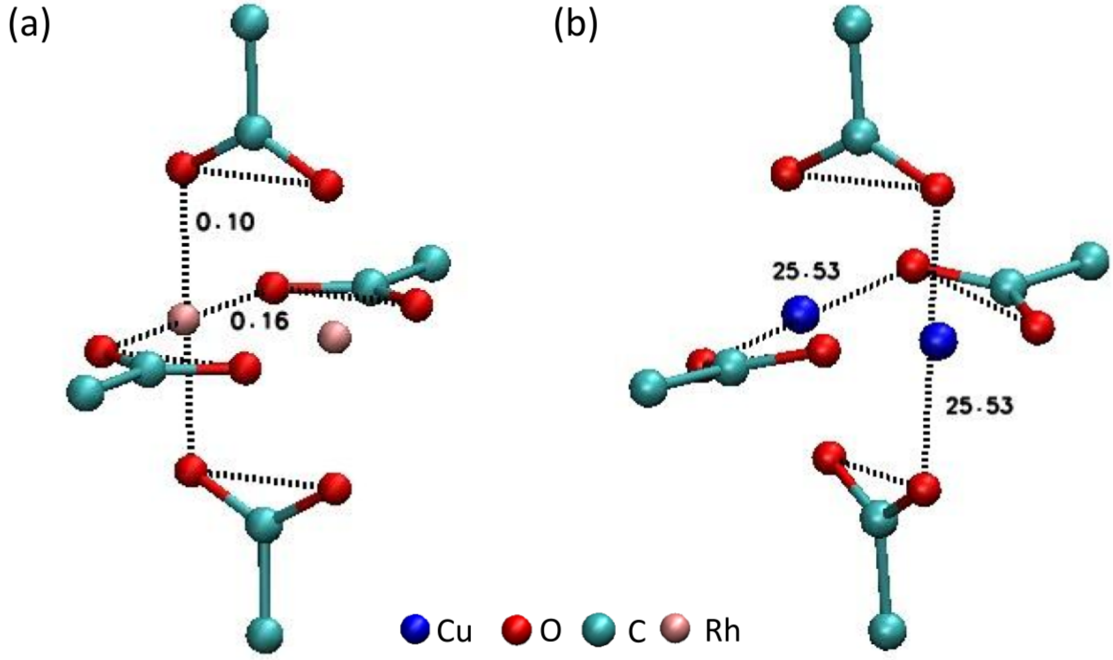

Figure S38. Selected dihedral angles (in degrees) for the structures reported in Figure S36. Selected O-O-O-O dihedral angles between oxygen atoms of opposite acetates in the paddlewheel for the DFT optimized structure in implicit water shown in Figure S36 (a) Rhodium acetate paddlewheel and (b) Copper acetate paddlewheel.

The difference between dihedral angles in both type of paddlewheels shown in Figure S38 suggest a possible mechanical origin of the different hydrolytic stability observed for Rh(II) and Cu(II) paddlewheels. While Rh(II) acetate forms highly symmetric paddlewheels, Cu(II) acetate suffers from a mechanical distortion creating a 25º distorted paddlewheel in which Cu-O distance remains constant at ~1.97 Å.

In order to further explore the effect of explicit interaction of water molecules to Cu(II) and Rh(II) paddlewheels, we studied the same systems in implicit water but adding two water molecules close to the axial positions of the paddlewheel cluster. Through this approach, we studied the effect of the physical added water in solution (implicit water environment) in the structure of Rh(II) and Cu(II) paddlewheels. In Figures S39–S41, we highlight the geometrical features of the resulting optimized structures. Note that these are the same structures that are shown in Figure 4 in the main paper.

The results show again striking differences between Rh(II) and Cu(II) paddlewheels. The comparison of the results obtained in the second set of calculations (including coordinating water molecules at axial positions, Figures S39–41) with those discussed before (without explicitly coordinating water molecules, Figures S36–S38) reveal substantial structural changes in the case of Cu(II) paddlewheel. On the other hand, the Rh(II) paddlewheel remains unaltered upon addition of the physically added water molecules.

In the case of the Rh(II) paddlewheel, all Rh-O(acetate) distances are identical to the ones observed in the first set of simulations (the case of only implicit water), being the length of all the Rh-O 2.08 Å (Figure S39). The water molecule oxygen is at 2.31 Å from the Rh(II) atom (Figure S39). In the case of the Cu(II) cluster, different Cu-O distances can be observed, being 1.98 Å the lower and 2.01–2.02 Å the higher Cu-O distances of the paddlewheel (Figure S39). These differences in the Cu-O distances within the paddlewheel induces torsion to the structure, which translates into a higher degree of deformation. Therefore, the Cu(II) paddlewheel is more distorted when the simulations are done in the presence of physical water.

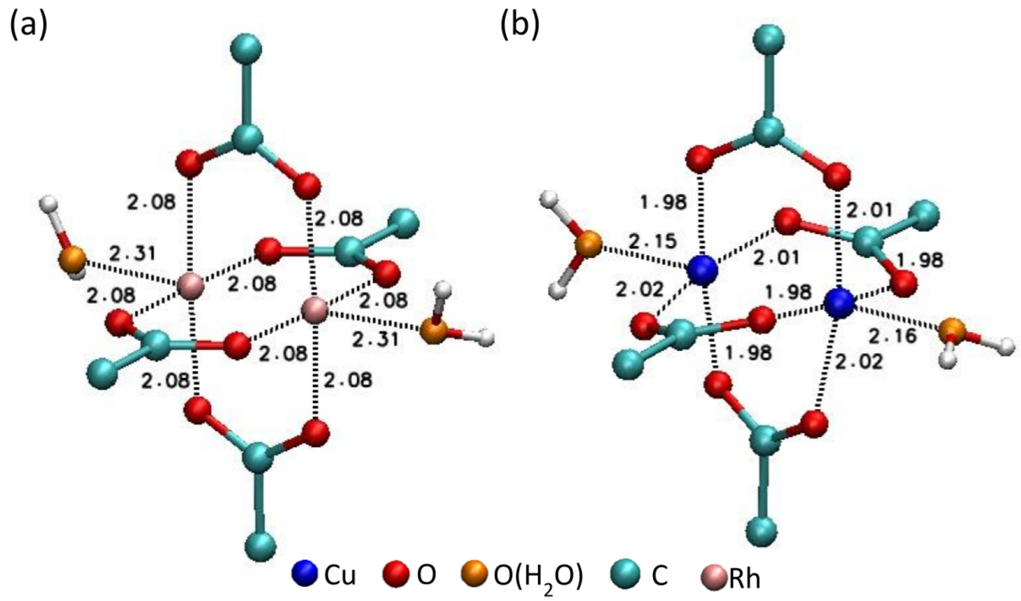

Figure S39. Results of DFT optimization of metal acetate paddlewheel structures with axially coordinating water molecules (second simulation set). DFT Optimized metal acetate paddlewheel structures in implicit water including two explicitly coordinating water molecules. Bond distances (in Å) are indicated between the metal and oxygen atoms. (a) Rhodium acetate paddlewheel and (b) Copper acetate paddlewheel, both of them with two axial water molecules.

The water induced torsion of the Cu(II) paddlewheel cluster translates into higher dihedral angles as shown in Figure S40 and S41 (see also Table S3). Thus, the Cu-O-O angles in the Cu(II) paddlewheel cluster are ~92º-95º and the dihedral angle defined by oxygen atoms of opposite acetates is 28º (Figures S40 and S41). In the case of the Rh(II) cluster, all Rh-O-O angles shown in Figure S40 are ~92º and the dihedrals angles defined by oxygen atoms of opposite acetates are negligible (Figure S41).

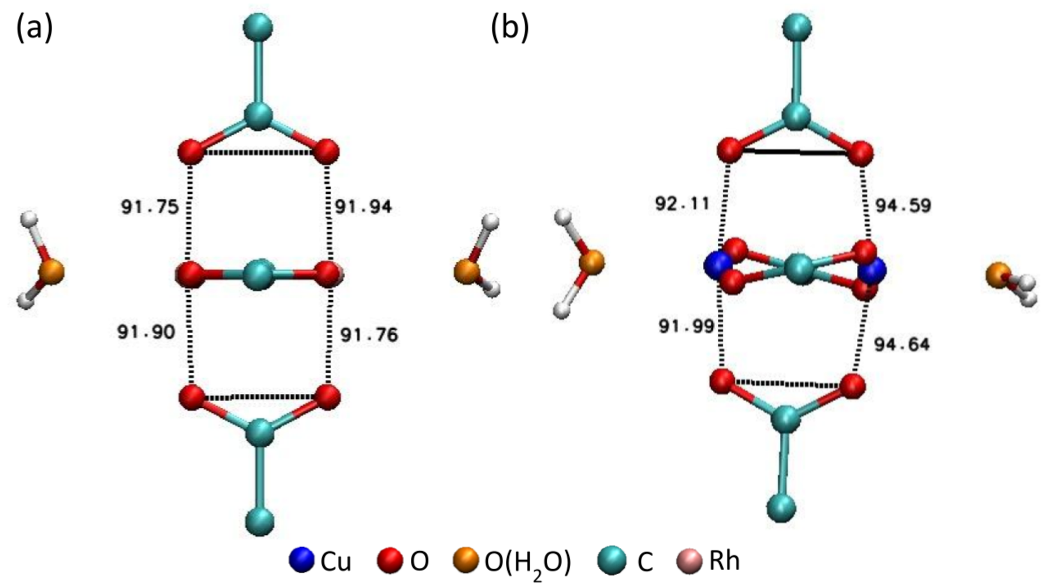

Figure S40. Selected angles (in degrees) for the structures reported in Figure S39. Selected metal-O-O angles for the DFT optimized paddlewheel structure in implicit solvation water shown in Figure S39, which includes two explicit water molecules. (a) Rhodium acetate paddlewheel and (b) Copper acetate paddlewheel, both of them with two axial water molecules.

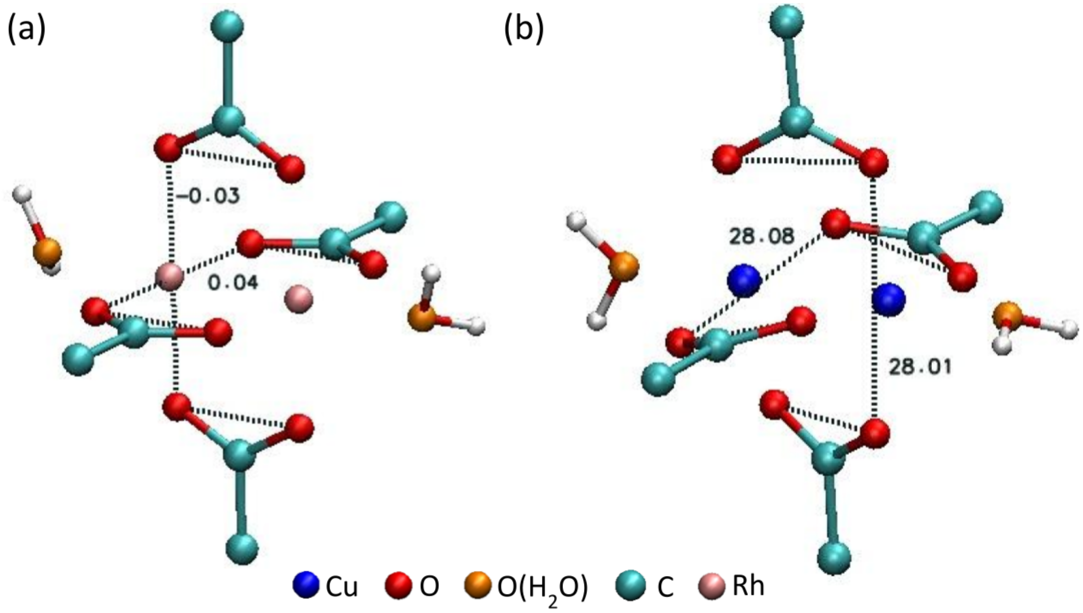

Figure S41. Selected dihedral angles (in degrees) for the structures reported in Figure S39. Selected O-O-O-O dihedral angles between oxygen atoms of opposite acetates in the paddlewheel for the same structures shown in Figure S39. (a) Rhodium acetate paddlewheel and (b) Copper acetate paddlewheel, both of them with two axial water molecules.

Therefore, our calculations suggest that the mechanical distortion of the Cu(II) paddlewheel when it is exposed to water is cardinal for understanding the hydrolysis of such clusters, which is related to the overall hydrolytic instability of Cu(II)-based HKUST-1. It should be noted that torsion obtained in the present work, considering always calculations of optimization of structures in solution, was not obtained when the calculations were performed in vacuo.[13,22] Interestingly, some key geometrical quantities obtained in our optimized Cu(II) structures are identical to those obtained in previous DFT and MP2 calculations in vacuo.[13,22] These include the Cu-Cu distance obtained in Figure S36, and the Cu-O(water) distance obtained in Figure S39. However, the Cu-O(acetate) distance and the angles and torsion angles obtained in our calculations in solution (Table S3) differ from those obtained for calculations in vacuo. Therefore, the torsions identified in our calculations of the Cu(II) based paddlewheel in water solution provide a plausible explanation for the experimentally observed instability and hydrolysis of Cu(II)-based HKUST-1.

The mechanism that we postulate is the following. We have seen that the coordination of water molecules to the axial positions of the Cu(II) paddlewheels increases the Cu-Cu distance from 2.53 Å to 2.61 Å (Table S3). This effect is in line with the electrostatic repulsion between Cu(II) ions to avoid the formation of paddlewheel clusters in water and with the experimentally observed tendency of Cu(II) paddlewheel-based MOFs to hydrolyze into structures with monometallic nodes rather than paddlewheel clusters.[20,23] Furthermore, the Cu(II)-water interaction induces the splitting of the Cu-O distance (1.97 Å, Figure S36) to two different set of distances as shown in Figure S39 (1.98 Å for two Cu-O distances and 2.01–2.02 Å for the other two). This Cu-O distance splitting has a simple mechanistic interpretation. In Figure S42 we have highlighted the set of Cu-O bonds with shorter distances from the structure in Figure S39. From this figure, it seems clear that this particular subset of distances corresponds to the formation of monodentate hydrated copper acetates, further supporting the water-induced formation of monodentate hydrated Cu(II) acetate species. We have also highlighted the O-H distance between acetate and water at ~3 Å (being the other H-O distance ~4 Å), which may be susceptible to an acid-base reaction that will finally promote the total hydrolysis of the copper paddlewheel.

Table S3. Summary of geometrical quantities in the DFT optimized structures. Summary of metal-metal distances and metal-oxygen distances, and selected dihedrals for the structures reported in Figures S36–41.

**Parameter**	**Implicit water**	**Implicit water + 2 water molecules**
	Distances (Å)	
Cu-Cu	2.53	2.61
Cu-O (Acetate)	1.97	1.98
	1.97	2.01
	1.97	1.98
	1.97	2.02
Cu-O (Water)	-	2.15
	-	2.16
	Dihedral Angles (°)	
O-O-O-O (Oxygen atoms from opposite acetates)	25.53	28.01
	25.53	28.08

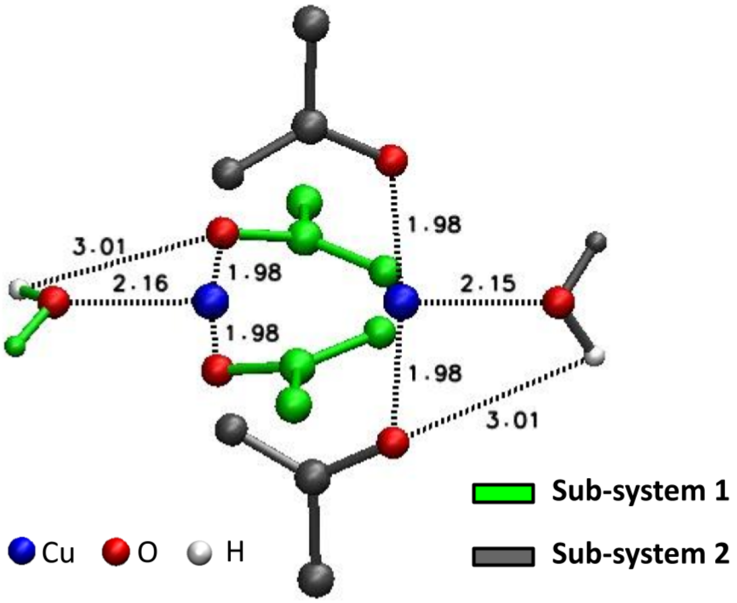

Figure S42. Optimized paddlewheel structures of the Cu system from Figure S41 (Cu acetate paddlewheel simulated in implicit water including two explicit water molecules) highlighting the key distances (in Å) for the starting point formation of two sub-systems through copper splitting and the torsion to promote the acid-base reaction.

Therefore, all the results derived from our simulation studies are in agreement with the hypothesis that the mechanical distortion of the Cu(II) paddlewheel is the pivotal step for the hydrolysis of these clusters. This mechanical distortion is completely absent in Rh(II) paddlewheels, which will explain the experimentally observed water stability of Rh(II) paddlewheel-based structures.[24] In the case of the RhCu-btc-HKUST-1, its stability can be explained by the presence of highly stable (and highly symmetric) rhodium paddlewheels through all the supramolecular structure, which block the mechanical distortion of the adjacent Cu(II) paddlewheels making the overall structure stable against hydrolysis

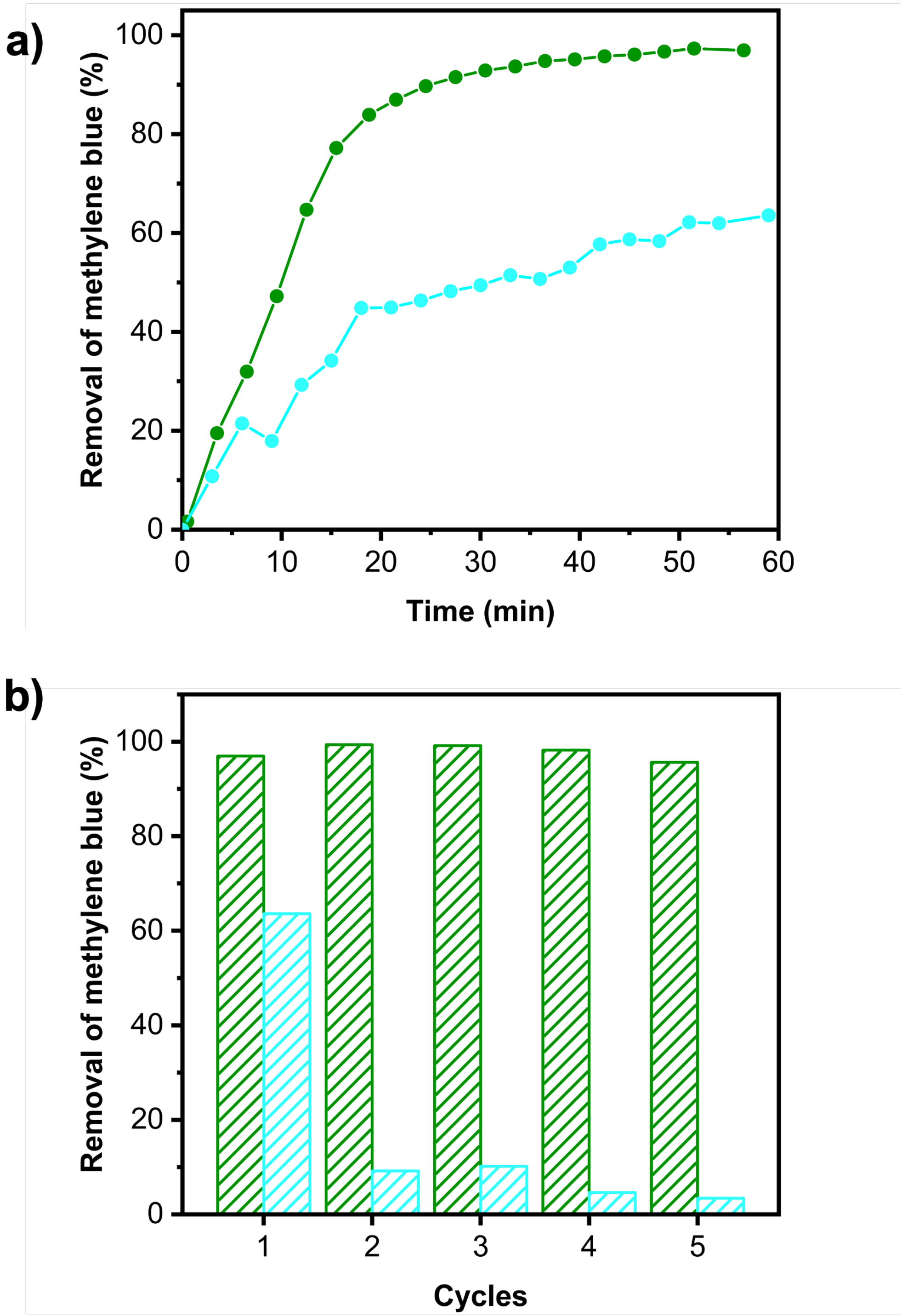

Figure S43 a) Removal of MB in water by RhCu-btc-HKUST-1 (green) and Cu(II)-HKUST-1 (blue) as a function of time. b) Five consecutive MB removal/regeneration cycles comparing the performance of RhCu-btc-HKUST-1 (green) and Cu(II)-HKUST-1 (blue). Conditions of each removal experiment: Volume of the solution= 20 ml; pH= 7; concentration of MB= 20 ppm; amount of RhCu-btc-HKUST-1 and Cu(II)-HKUST-1 used: 5 mg.

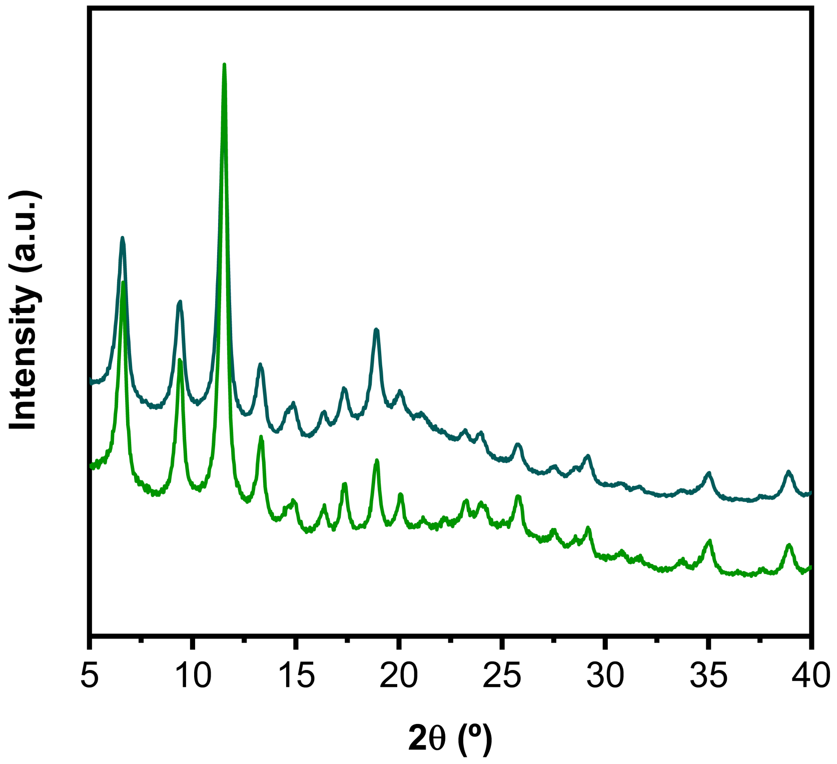

Figure S44 PXRD of as-made RhCu-btc-HKUST-1 (green) and after the methylene blue removal study (blue). Note that the crystallinity of RhCu-btc-HKUST-1 is maintained after the methylene blue removal conditions.

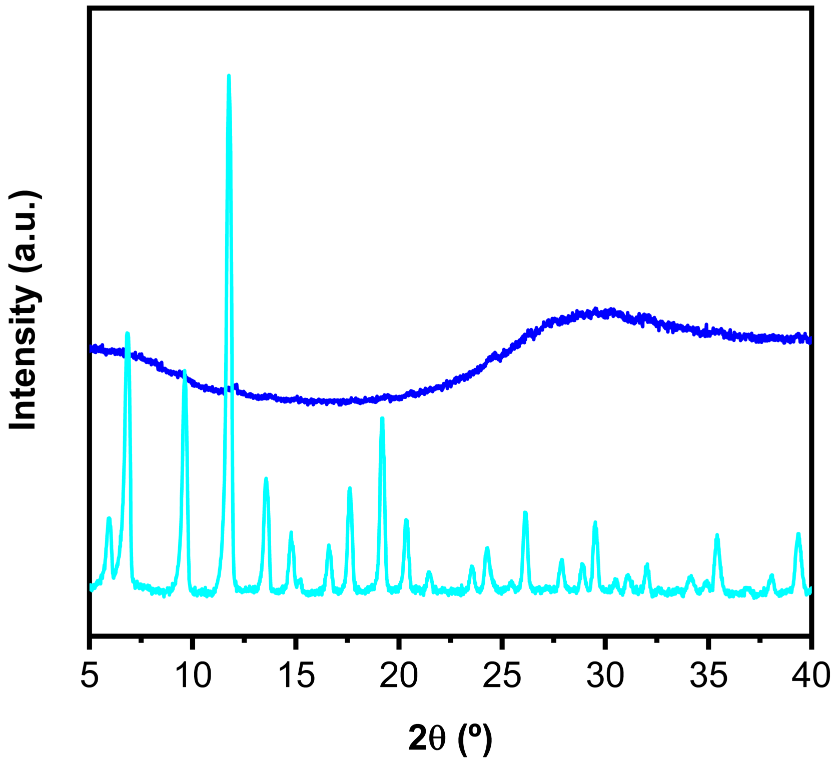

Figure S45 PXRD of as-made Cu(II)-HKUST-1 (light blue) and after the methylene blue adsorption study (blue). Note that Cu(II)-HKUST-1 degrades and becomes amorphous under the aqueous methylene blue removal conditions.

S5. Characterization of RhCu-(Br)btc-HKUST-1.

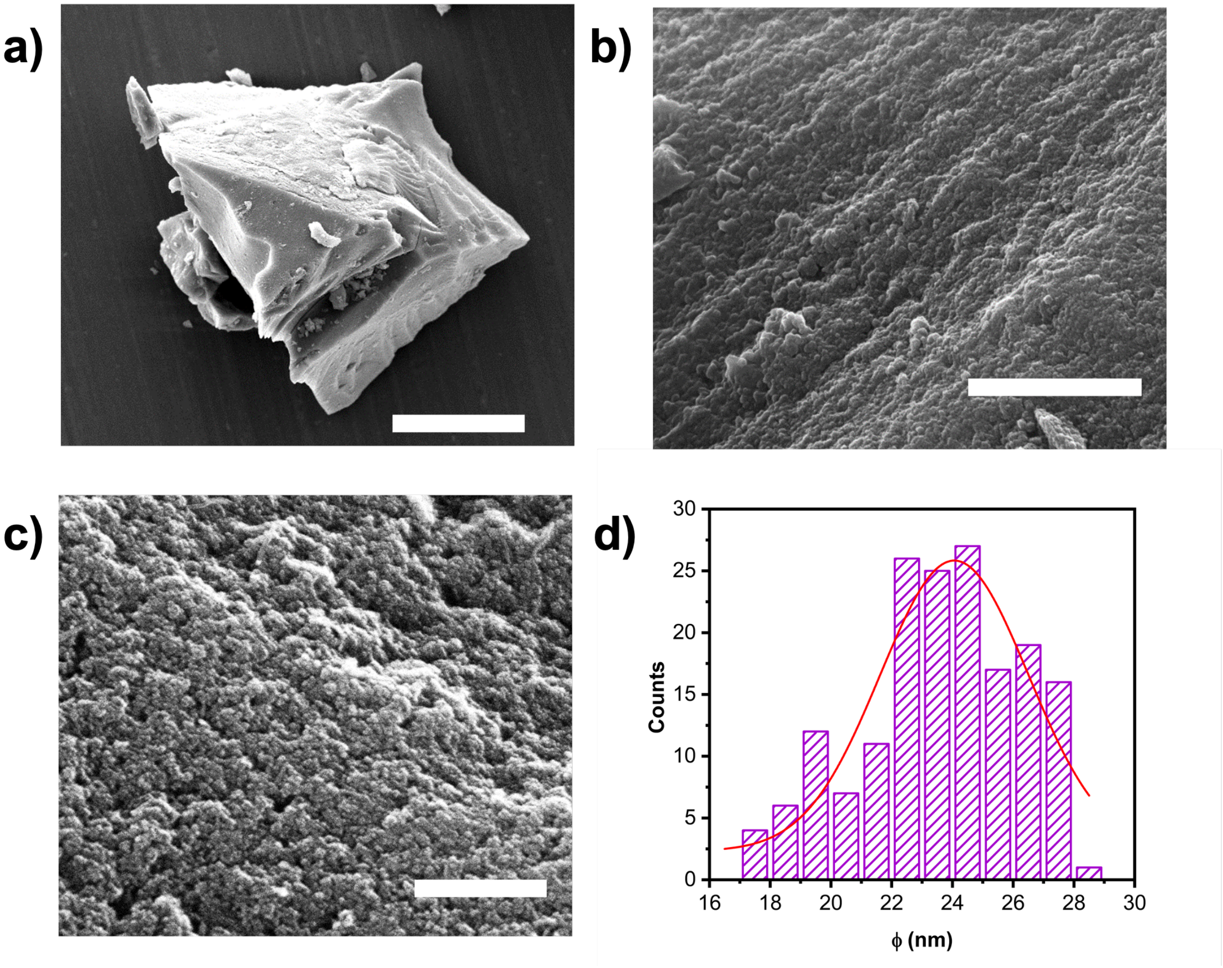

Figure S46 a) Representative FESEM image of a monolithic aggregate of RhCu-(Br)btc-HKUST-1 particles. (b, c) High magnification FESEM images of the surface of the monolithic aggregate showing that it is composed of the close packing of nanocrystals. d) Particle size distribution histogram of RhCu-(Br)btc-HKUST-1 with a mean particle size of 24 ± 2 nm. Scale bars: 50 μm (a) 2 μm (b), and 500 nm (c).

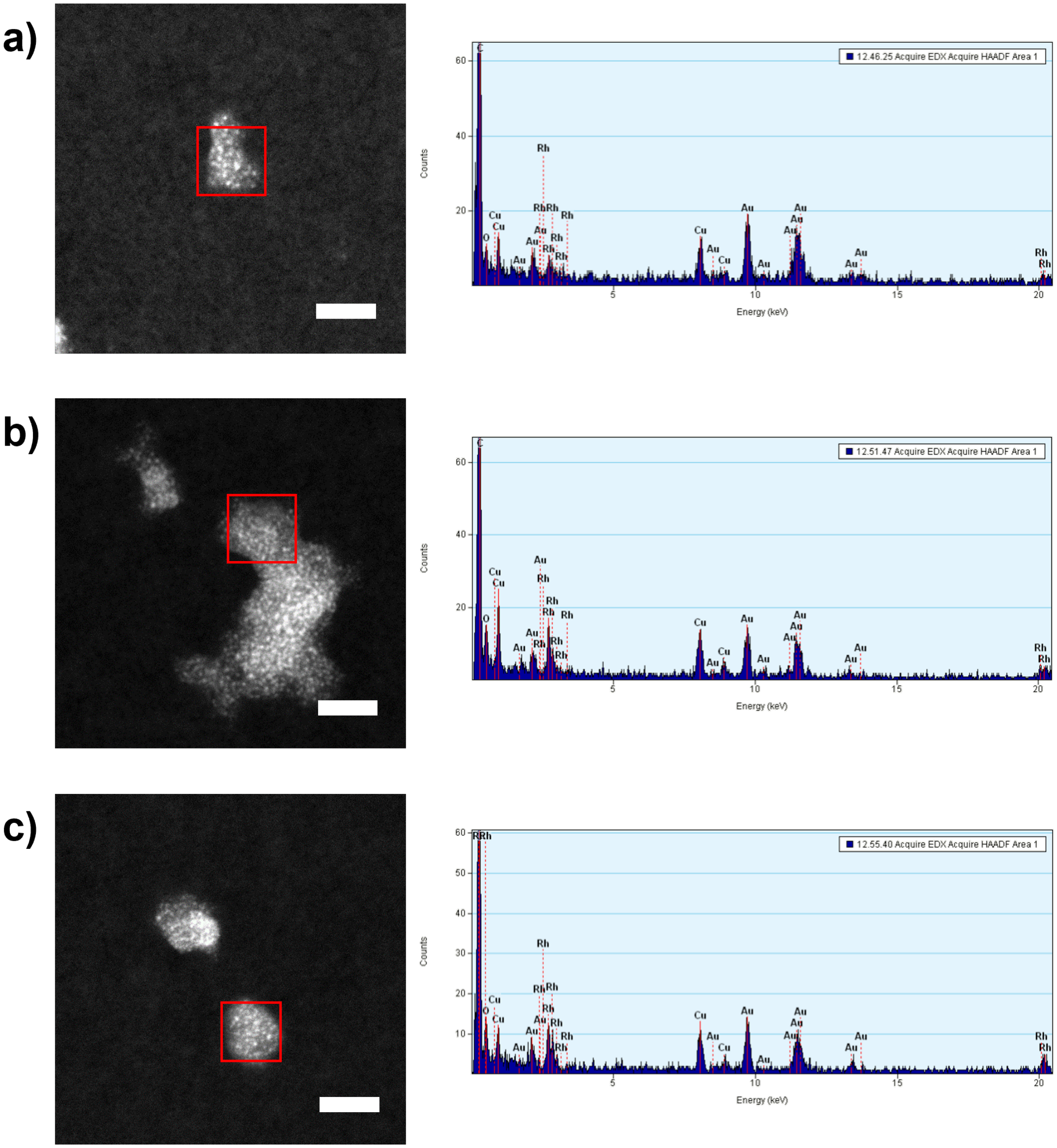

Figure S47 (a-c) Representative HAADF-STEM images (left) and the corresponding EDX spectra (right) obtained from single RhCu-(Br)btc-HKUST-1 crystals. Scale bars: 20 nm (a, b, c). Note that Au signals stem from the TEM grid used for these measurements.

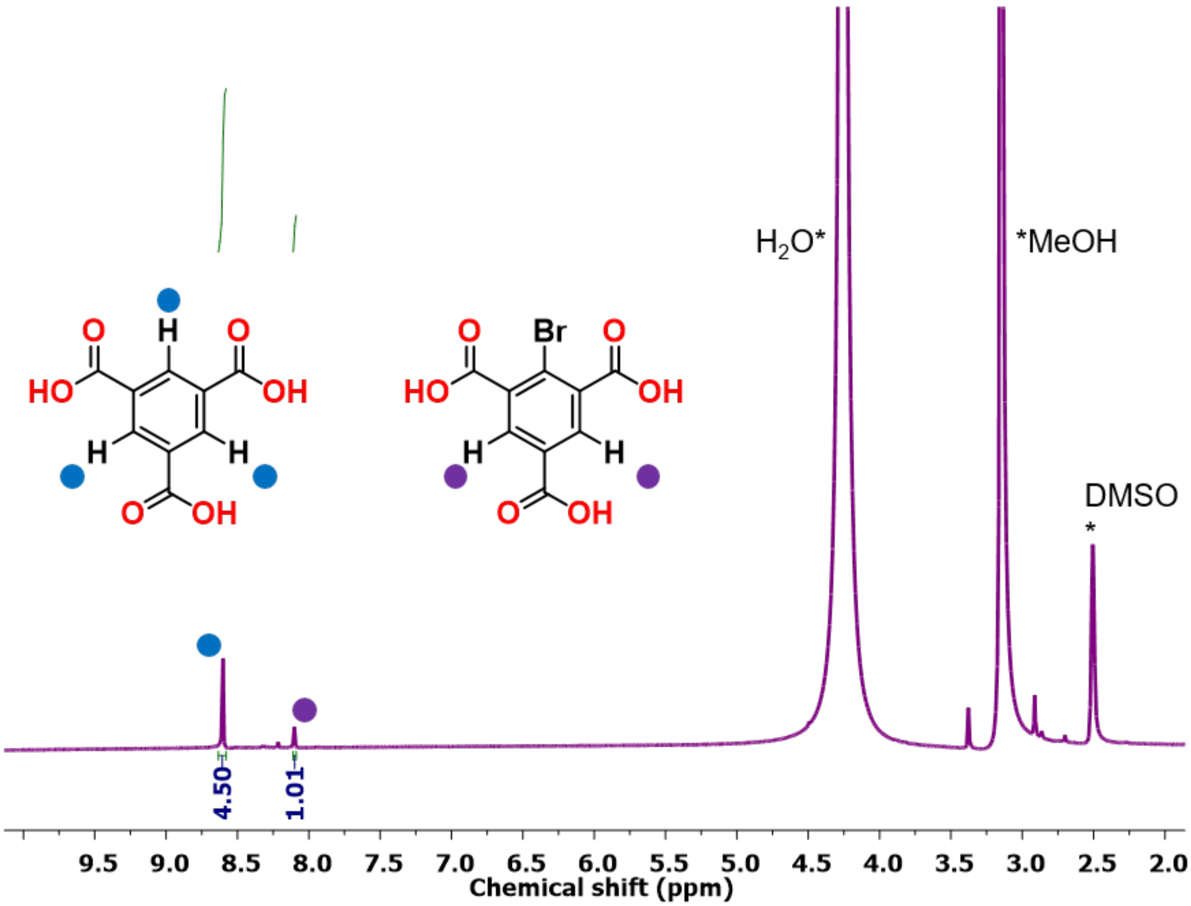

Figure S48 ^{1}H NMR spectra (300 MHz, DMSO-d_6) of fully digested RhCu-(Br)btc-HKUST-1. Note that the connectivity of RhCu-(Br)btc-HKUST-1 defines a COOH-RhMOP:(Br)btc ratio of 1:8. As the complete acid digestion of COOH-RhMOP should release 24 btc linkers, the expected btc:(Br)btc molar ratio should be 24:8 or 3:1. Moreover, taking into account that each signal of btc integrates three protons (blue dot) and that each signal of (Br)btc integrates two protons (purple dot), the ratio of the signals ascribed to btc and (Br)btc should be 4.5:1. Therefore, the ^{1}H-NMR of the fully digested RhCu-(Br)btc-HKUST-1 confirms that the btc:(Br)btc molar ratio is indeed 3:1.

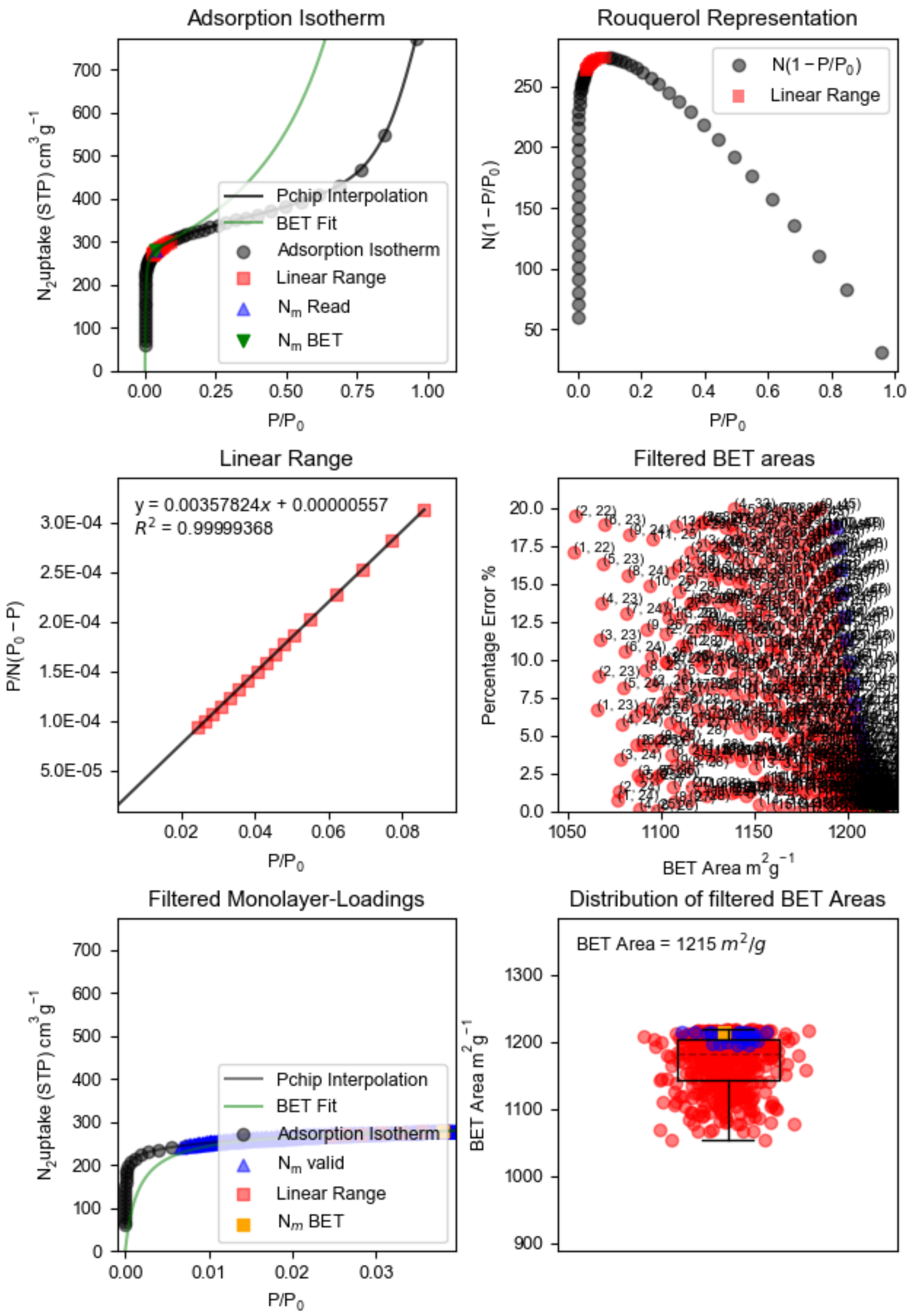

Figure S49 BETSI analysis of RhCu-(Br)btc-HKUST-1.

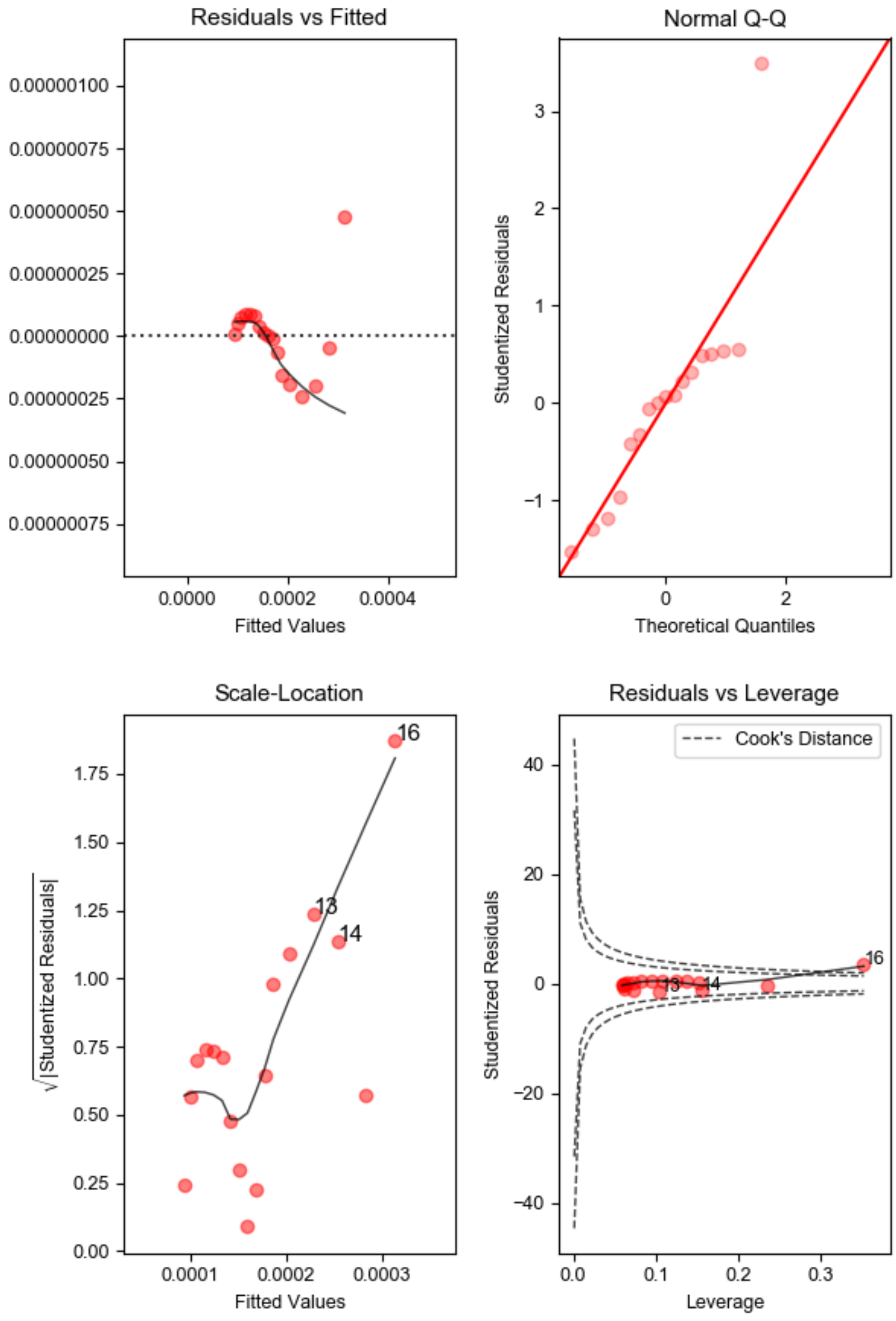

Figure S50 BETSI regression diagnostics of RhCu-(Br)btc-HKUST-1.

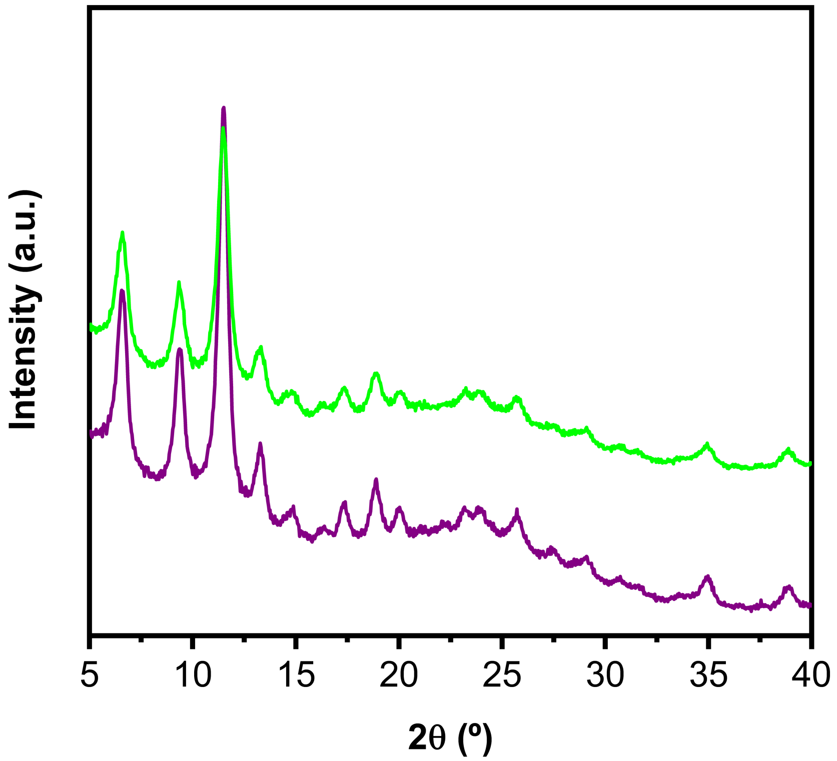

Figure S51 PXRD of as-made RhCu-(Br)btc-HKUST-1 (purple) and activated RhCu-(Br)btc-HKUST-1 (green).

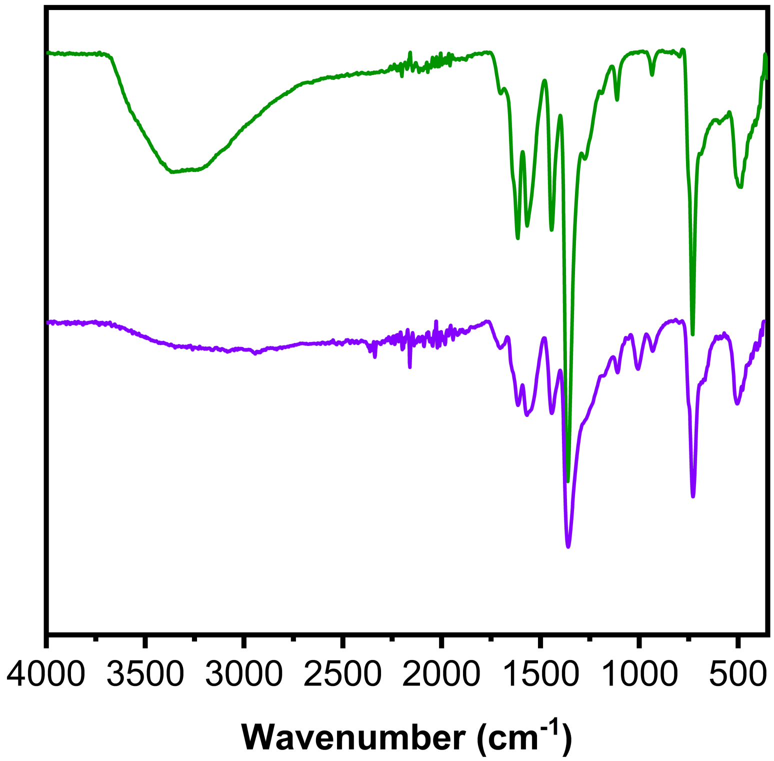

Figure S52 Full FT-IR spectra of RhCu-btc-HKUST-1 (green) and RhCu-(Br)btc-HKUST-1 (purple).

S5. Characterization of RhCu-(NO_2)btc-HKUST-1.

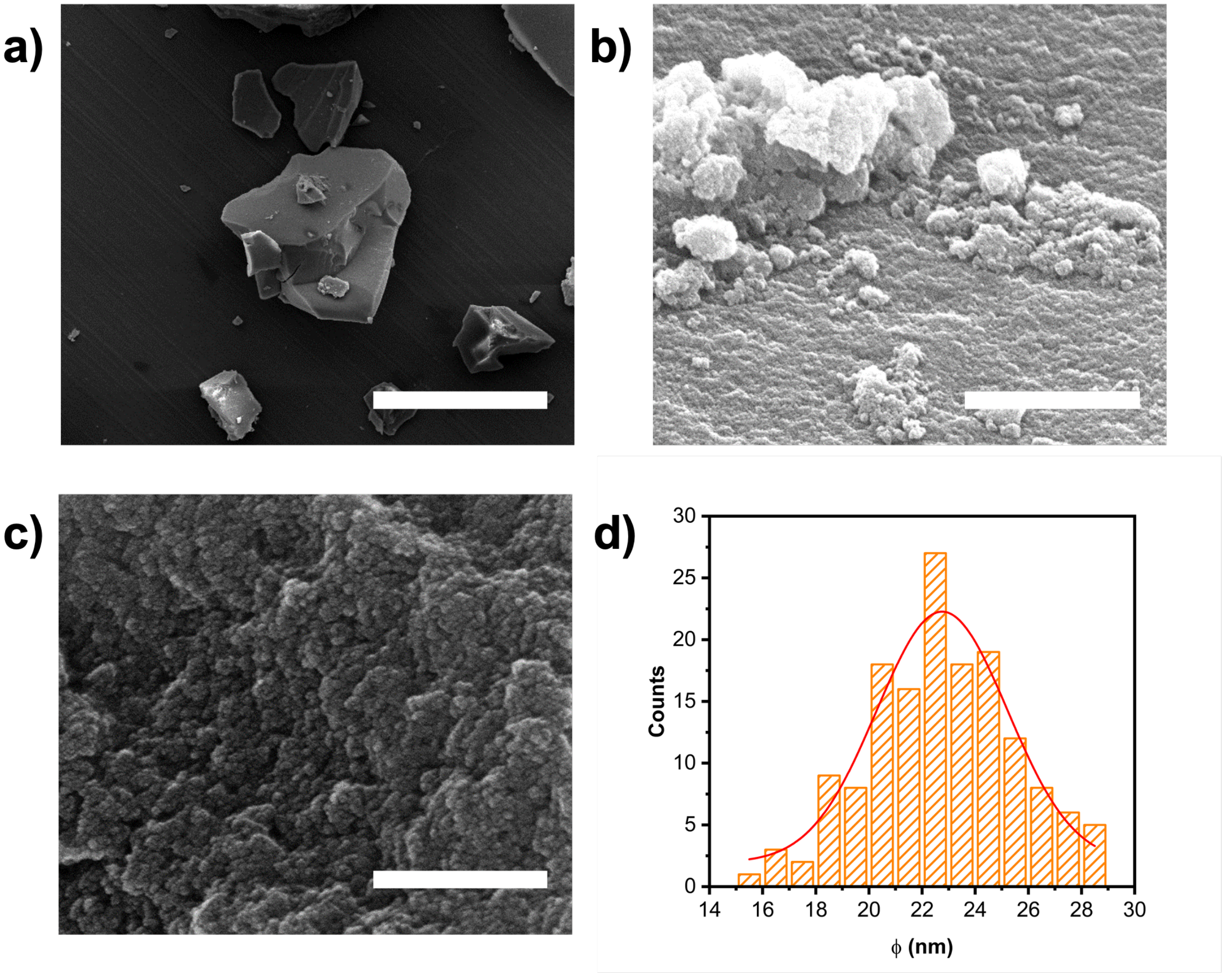

Figure S53 a) FESEM image of monolithic aggregate of RhCu-(NO_2)btc-HKUST-1 nanoparticles. (b, c) High magnification images of the surface of the monolithic aggregate showing that it is composed of the close packing of nanoparticles. d) Particle size distribution histogram of RhCu-(NO_2)btc-HKUST-1 with a mean particle size of 23 ± 2 nm. Scale bars: 200 µm (a), 2 µm (b), and 400 nm (c).

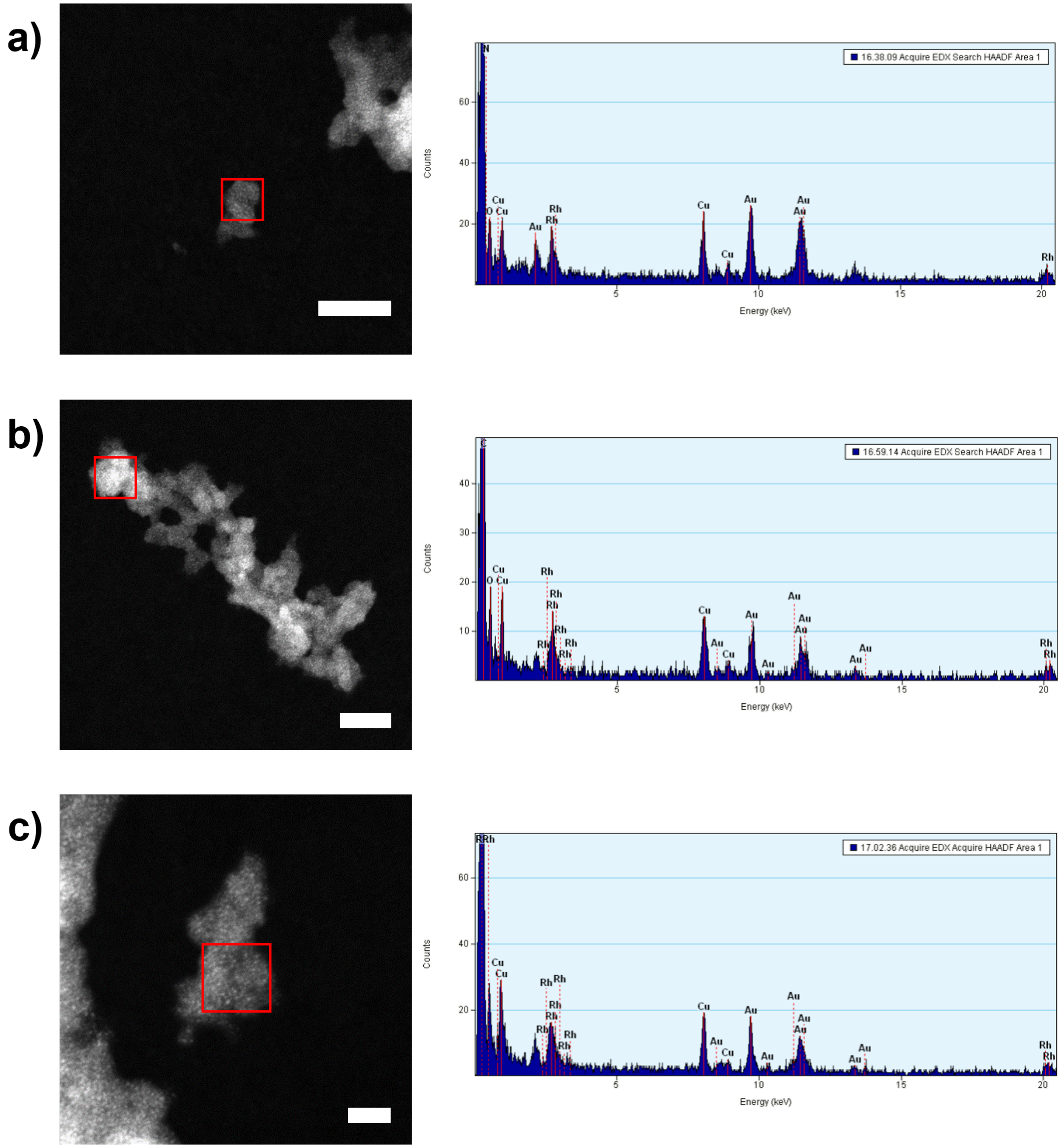

Figure S54 (a-c) Representative HAADF-STEM images (left) and the corresponding EDX spectra (right) collected in single RhCu-(NO_2)btc-HKUST-1 nanoparticles. Scale bars: 50 nm (a, b) and 20 nm (c). Note that Au signals stem from the TEM grid used for these measurements.

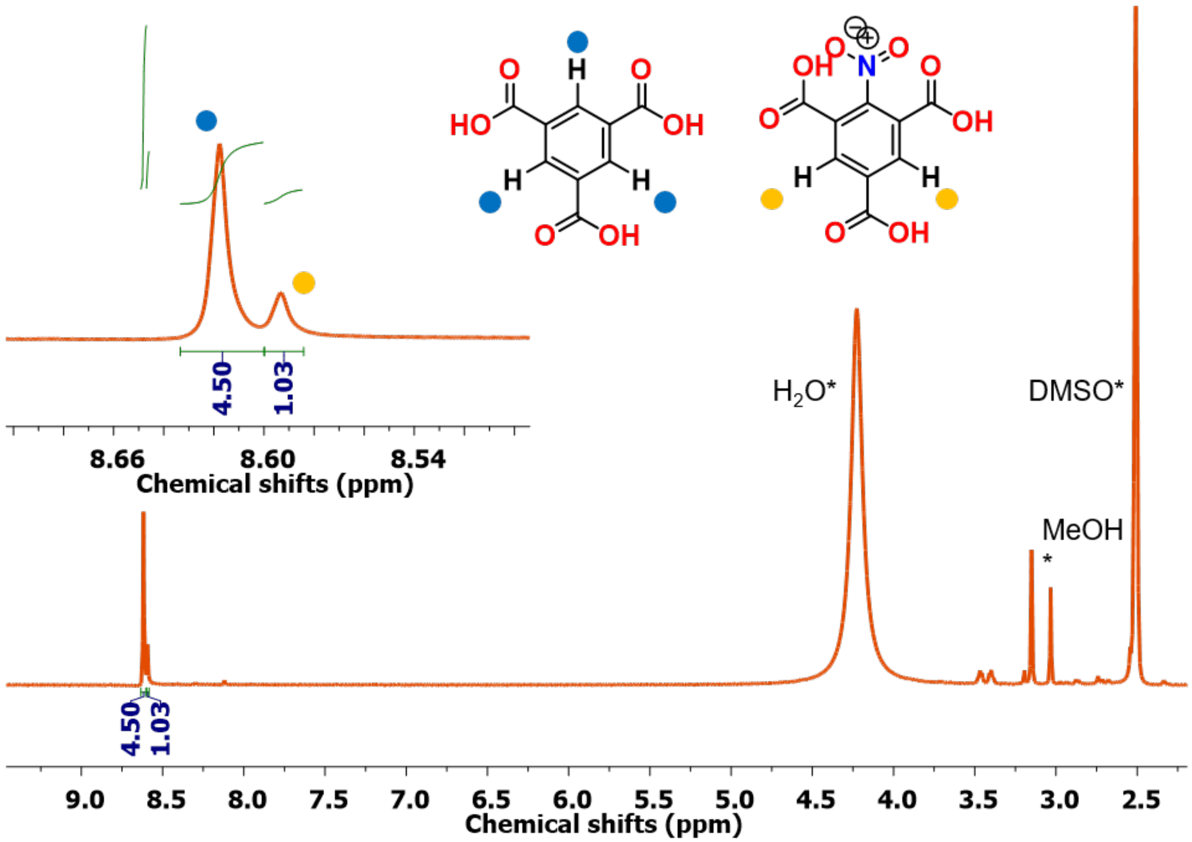

Figure S55 ^{1}H NMR spectra (300 MHz, DMSO-d_6) of fully digested RhCu-(NO_2)btc-HKUST-1. Note that the connectivity of RhCu-(NO_2)btc-HKUST-1 defines a COOH-RhMOP:(NO_2)btc ratio of 1:8. As the complete acid digestion of COOH-RhMOP should release 24 btc linkers, the expected btc:(NO_2)btc molar ratio should be 24:8 or 3:1. Moreover, taking into account that each signal of btc integrates three protons (blue dot) and that each signal of (NO_2)btc integrates two protons (yellow dot), the ratio of the signals ascribed to btc and (NO_2)btc should be 4.5:1. Therefore, the ^{1}H-NMR spectra of the fully digested RhCu-(NO_2)btc-HKUST-1 confirms that the btc:(NO_2)btc molar ratio is 3:1.

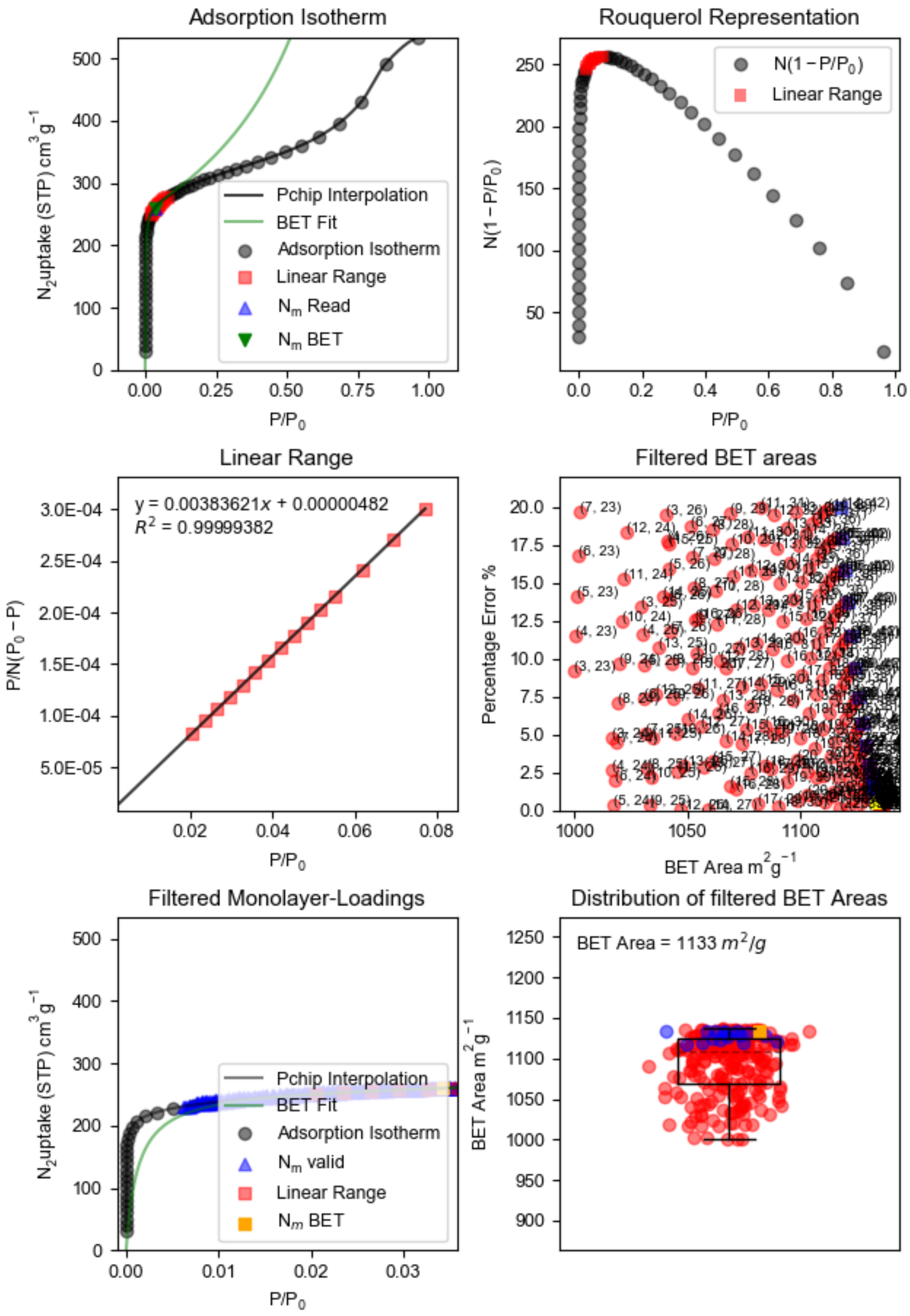

Figure 56 BETSI analysis of RhCu-(NO_2)btc-HKUST-1.

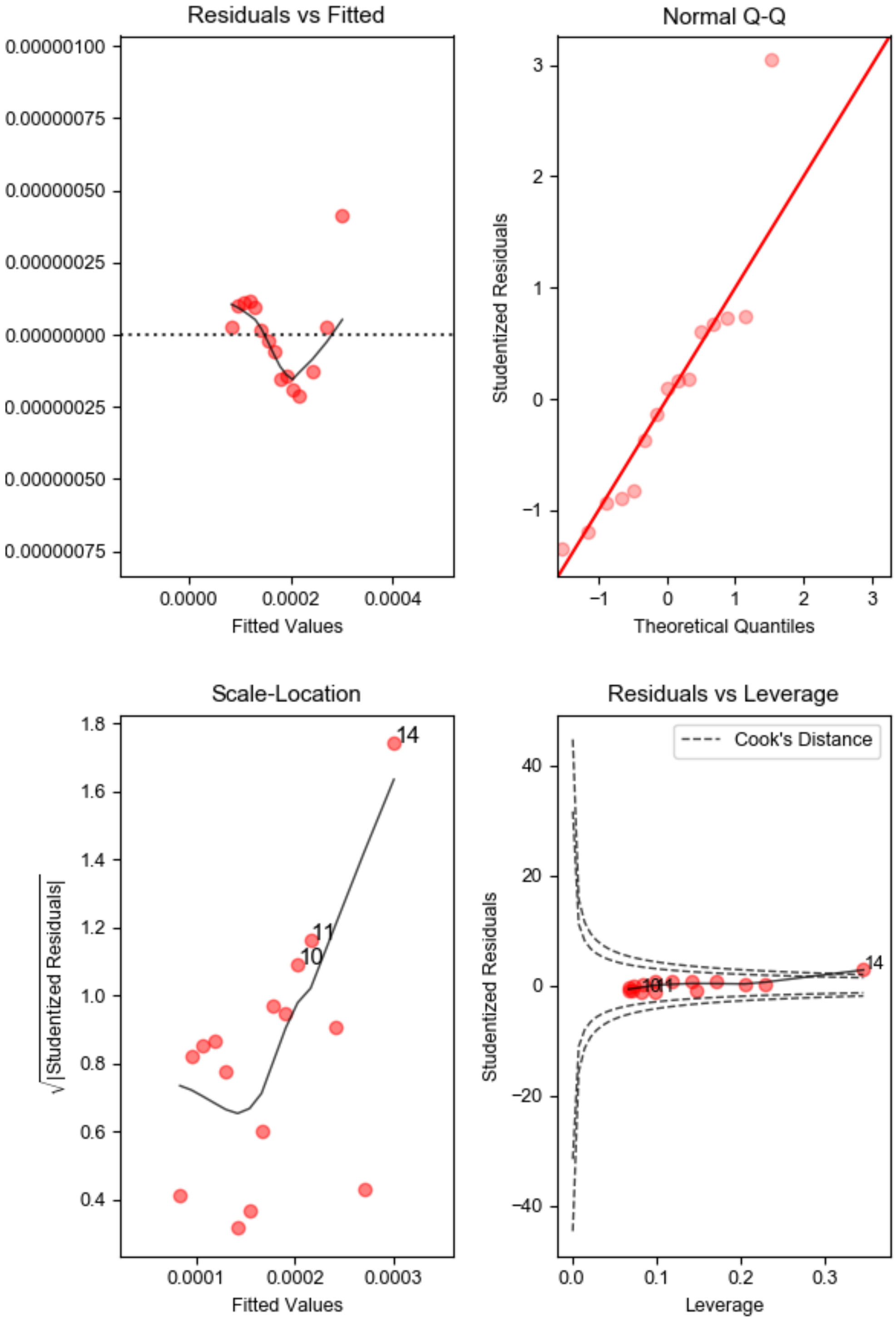

Figure S57 BETSI regression diagnostics of RhCu-(NO_2)btc-HKUST-1.

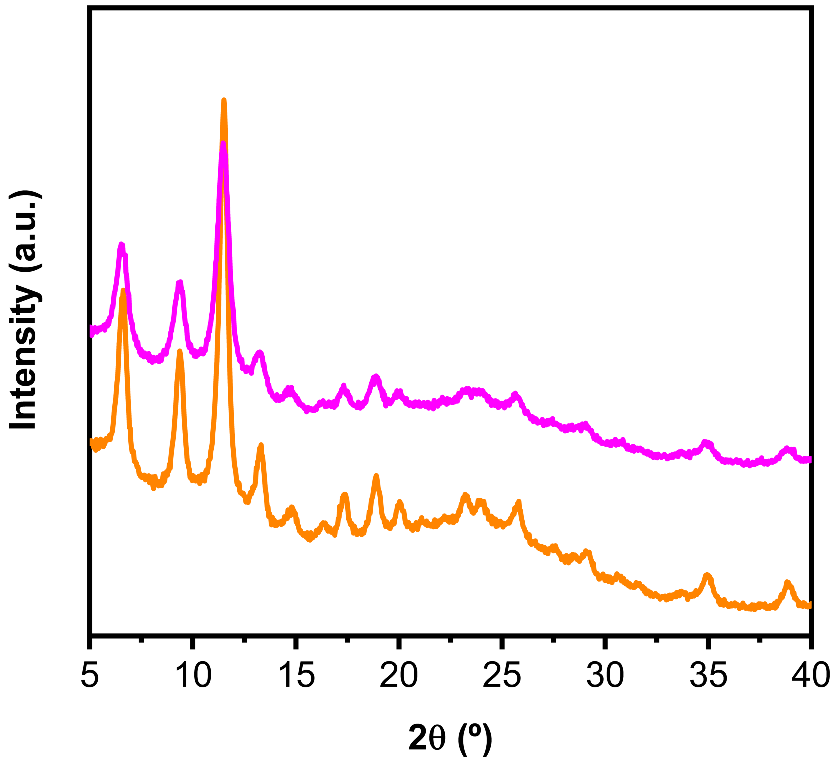

Figure S58 PXRD of as-made RhCu-(NO_2)btc-HKUST-1 (orange) and activated RhCu-(NO_2)btc-HKUST-1 (pink).

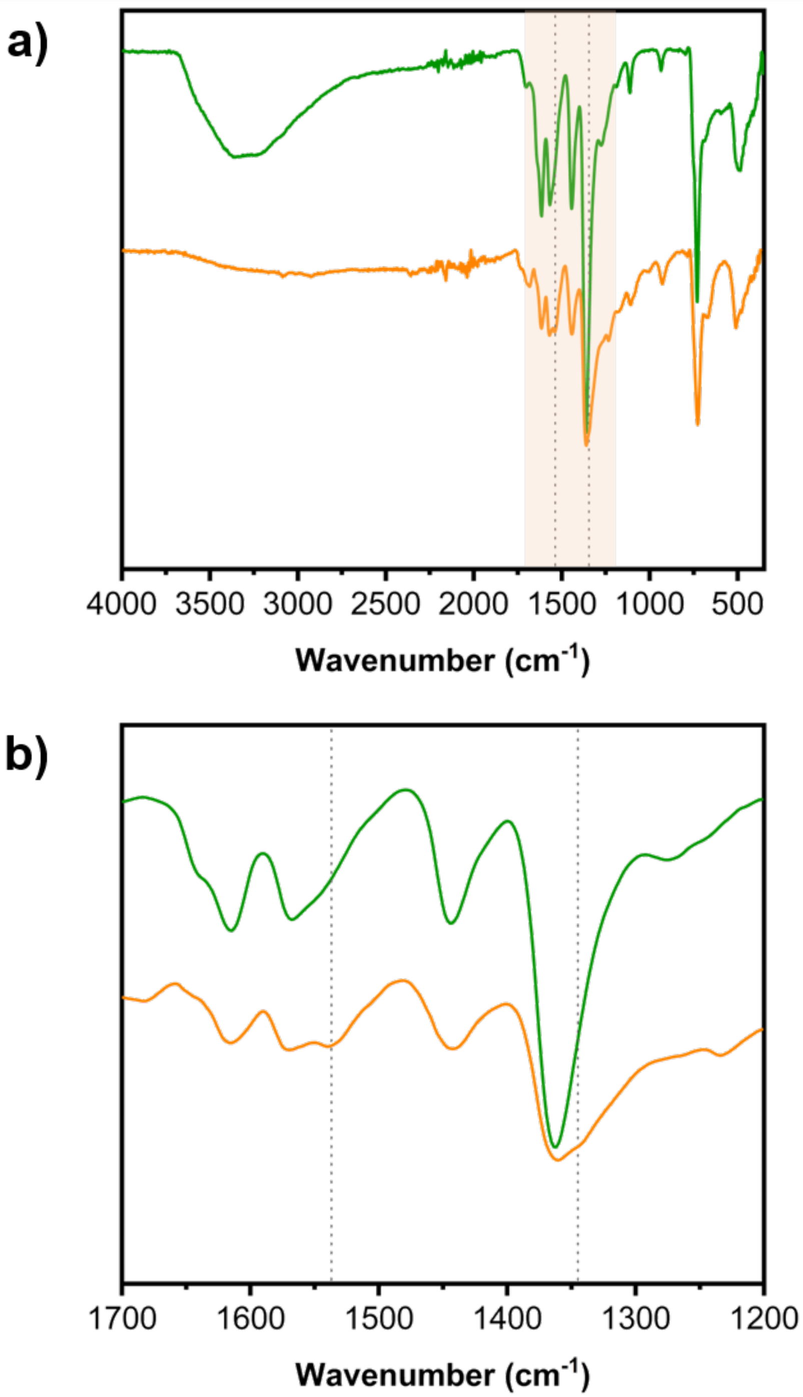

Figure S59 (a) Full FT-IR spectra of RhCu-btc-HKUST-1 (green) and RhCu-(NO_2)btc-HKUST-1 (orange), and (b) magnified area of the spectra. Note that RhCu-(NO_2)btc-HKUST-1 presents two additional peaks at 1345 and 1537 cm^{-1} compared to RhCu-btc-HKUST-1. This could be ascribed to the symmetric and asymmetric N-O stretching bands of nitro groups of the functionalized (NO_2)btc linker.

S6. Characterization of RhCu-(NH_2)btc-HKUST-1.

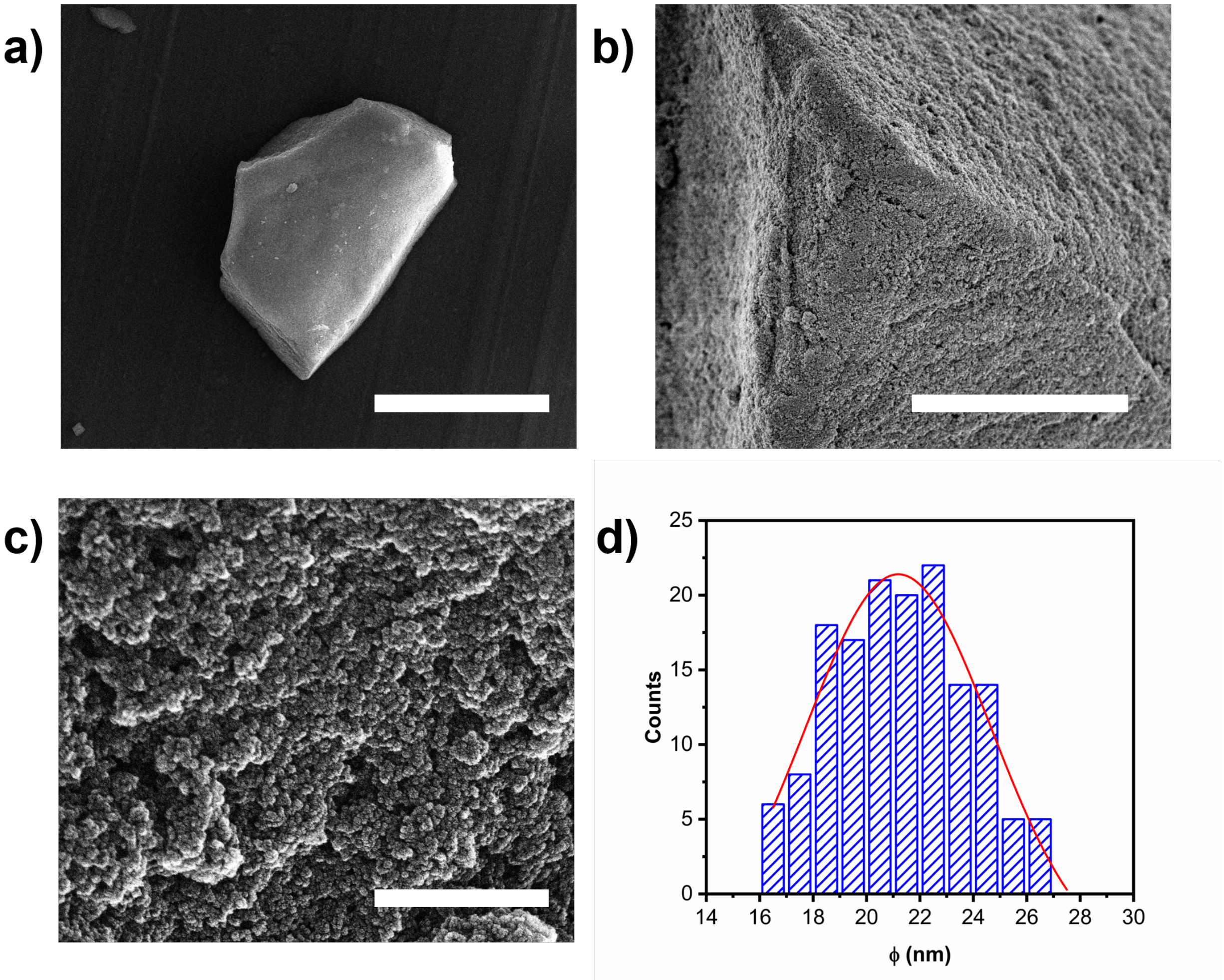

Figure S60 a) FESEM image of monolithic aggregate of RhCu-(NH_2)btc-HKUST-1 nanoparticles. (b, c) High magnification images of the surface of the monolithic aggregate showing that it is composed of the close packing of nanoparticles. d) Particle size distribution histogram of RhCu-(NH_2)btc-HKUST-1 with a mean particle size of 21 ± 3 nm. Scale bars: 50 μm (a), 5 μm (b), and 500 nm (c).

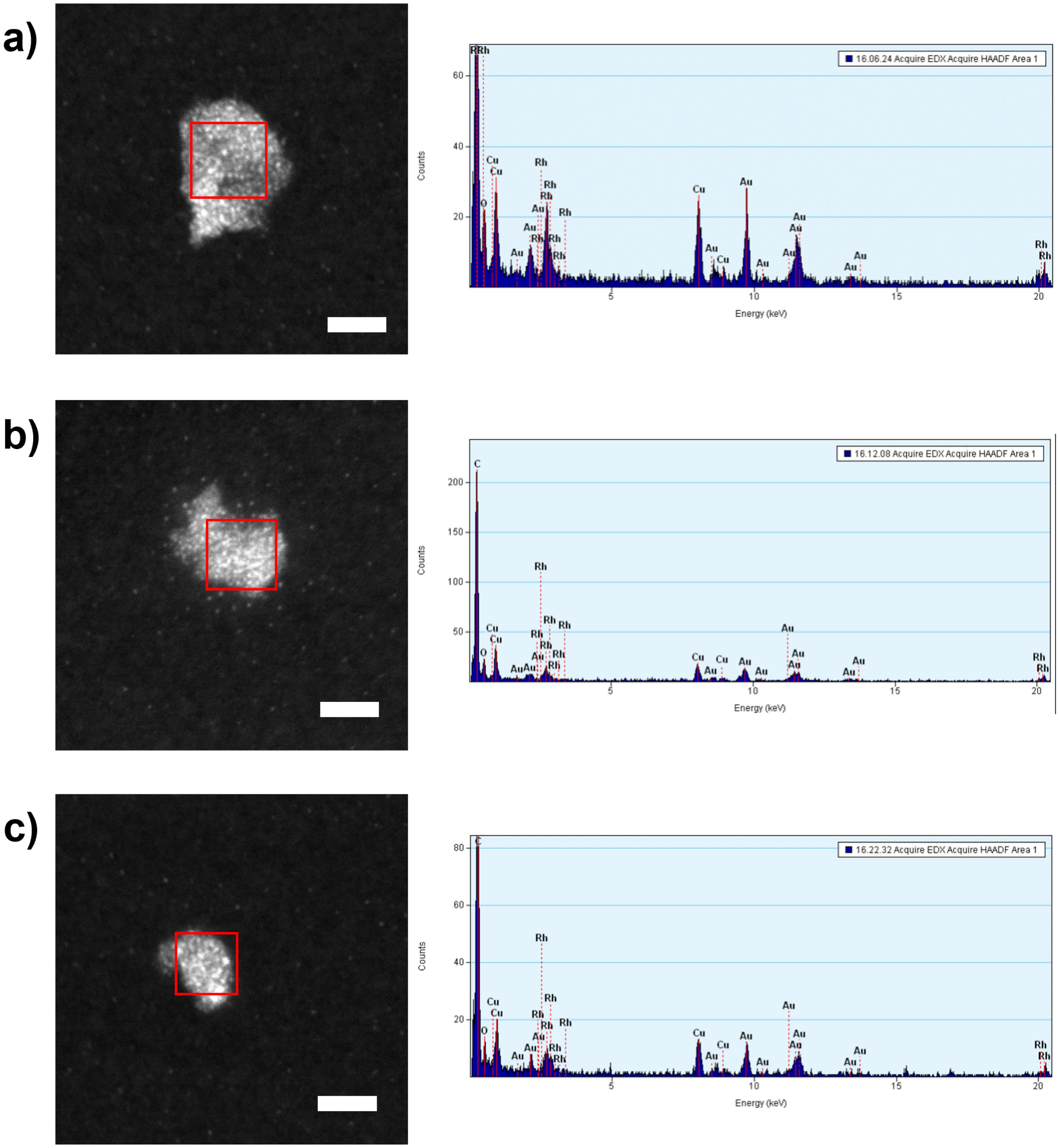

Figure S61 (a-c) Representative HAADF-STEM images (left) and the corresponding EDX spectra (right) collected in single RhCu-(NH_2)btc-HKUST-1 particles (right). Scale bars: 20 nm (a, b, c). Note that Au signals stem from the TEM grid used for these measurements.

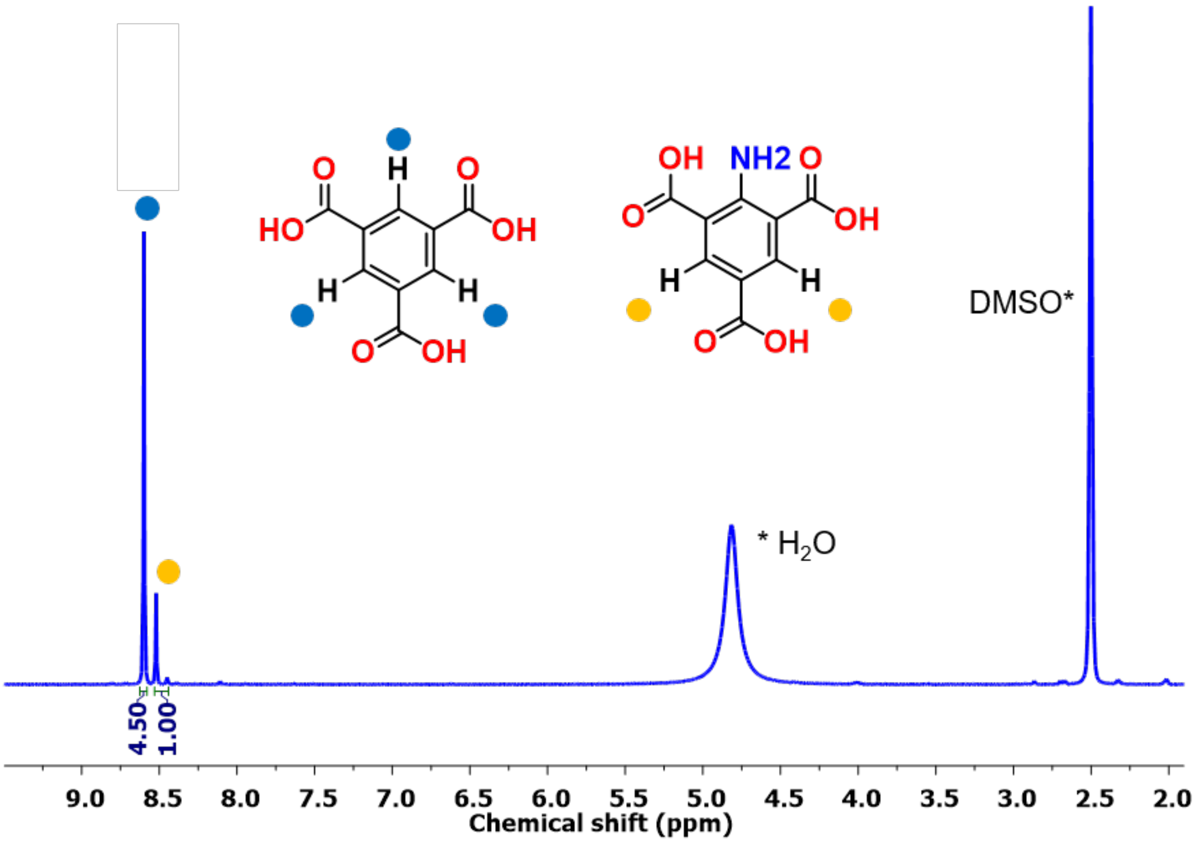

Figure S62 ^{1}H NMR spectra (300 MHz, DMSO-d_6) of fully digested RhCu-(NH_2)btc-HKUST-1. Note that the connectivity of RhCu-(NH_2)btc-HKUST-1 should define a COOH-RhMOP:(NH_2)btc ratio of 1:8. As the complete acid digestion of COOH-RhMOP should release 24 btc linkers, the expected btc:(NH_2)btc molar ratio should be 24:8 or 3:1. Moreover, taking into account that each signal of btc integrates three protons (blue dot) and that each signal of (NH_2)btc integrates two protons (yellow dot), the ratio of the signals ascribed to btc and (NH_2)btc should be 4.5:1. Therefore, the ^{1}H-NMR spectra of the fully digested RhCu-(NH_2)btc-HKUST-1 confirms that the btc:(NH_2)btc molar ratio is indeed 3:1.

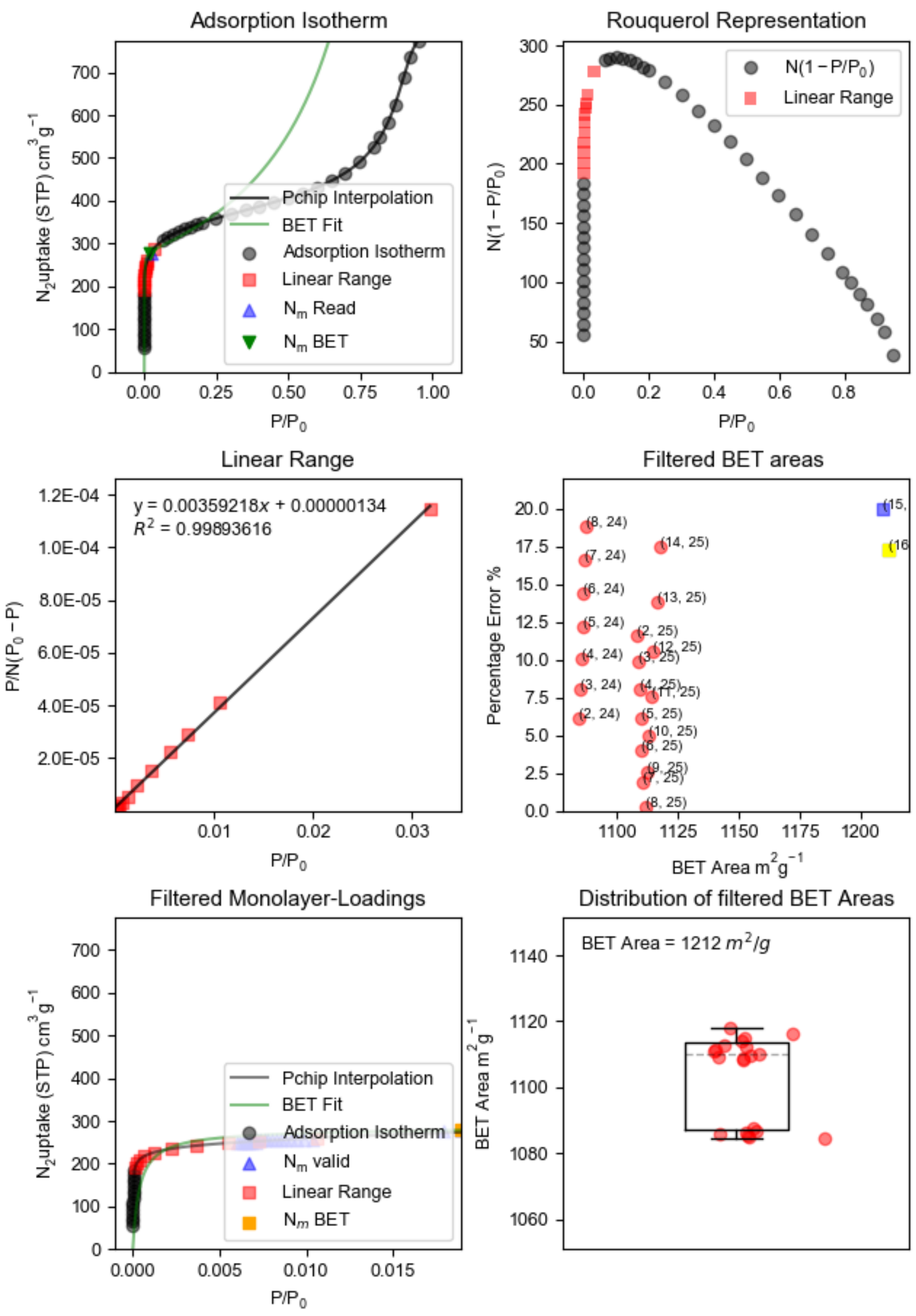

Figure 63 BETSI analysis of RhCu-(NH_2)btc-HKUST-1.

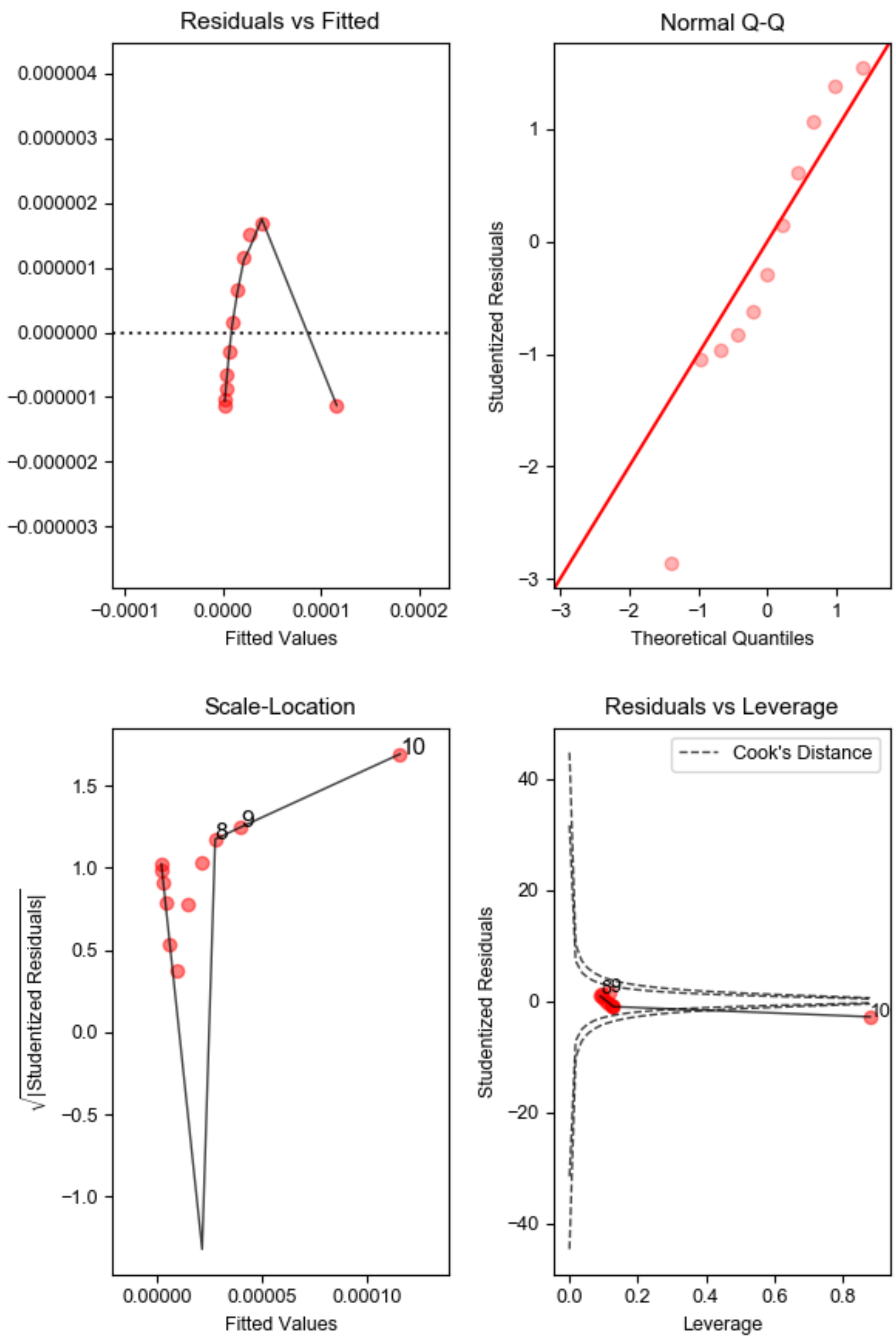

Figure S64 BETSI regression diagnostics of RhCu-(NH_2)btc-HKUST-1.

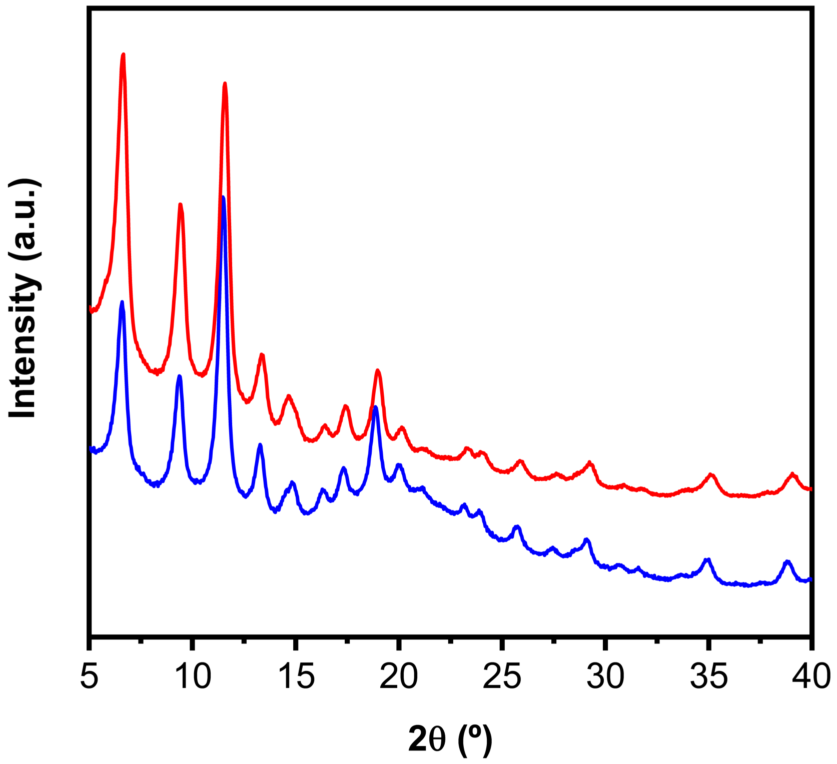

Figure S65 PXRD of the as-made RhCu-(NH_2)btc-HKUST-1 (blue) and activated RhCu-(NH_2)btc-HKUST-1 (red).

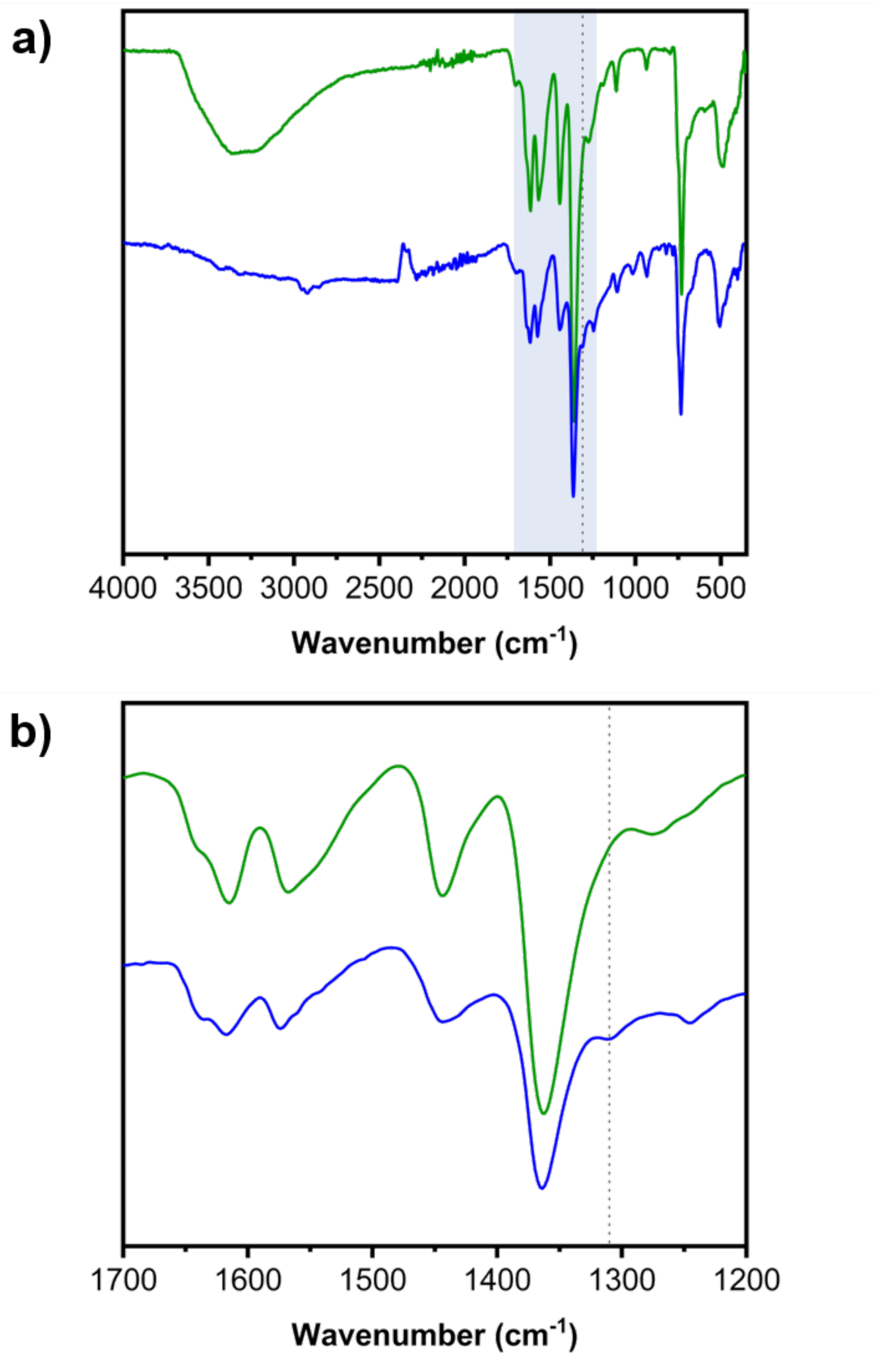

Figure S66 (a) Full FT-IR spectra of RhCu-btc-HKUST-1 (green) and RhCu-(NH_2)btc-HKUST-1 (blue), and (b) magnified area of the spectra. Note that RhCu-(NH_2)btc-HKUST-1 presents a shoulder at 1310 cm^{-1}, which is ascribed to the C-N stretching band of the aromatic amino groups of the functionalized (NH_2)btc linker.

S8. Characterization of RhCu-(COOH)btc-HKUST-1.

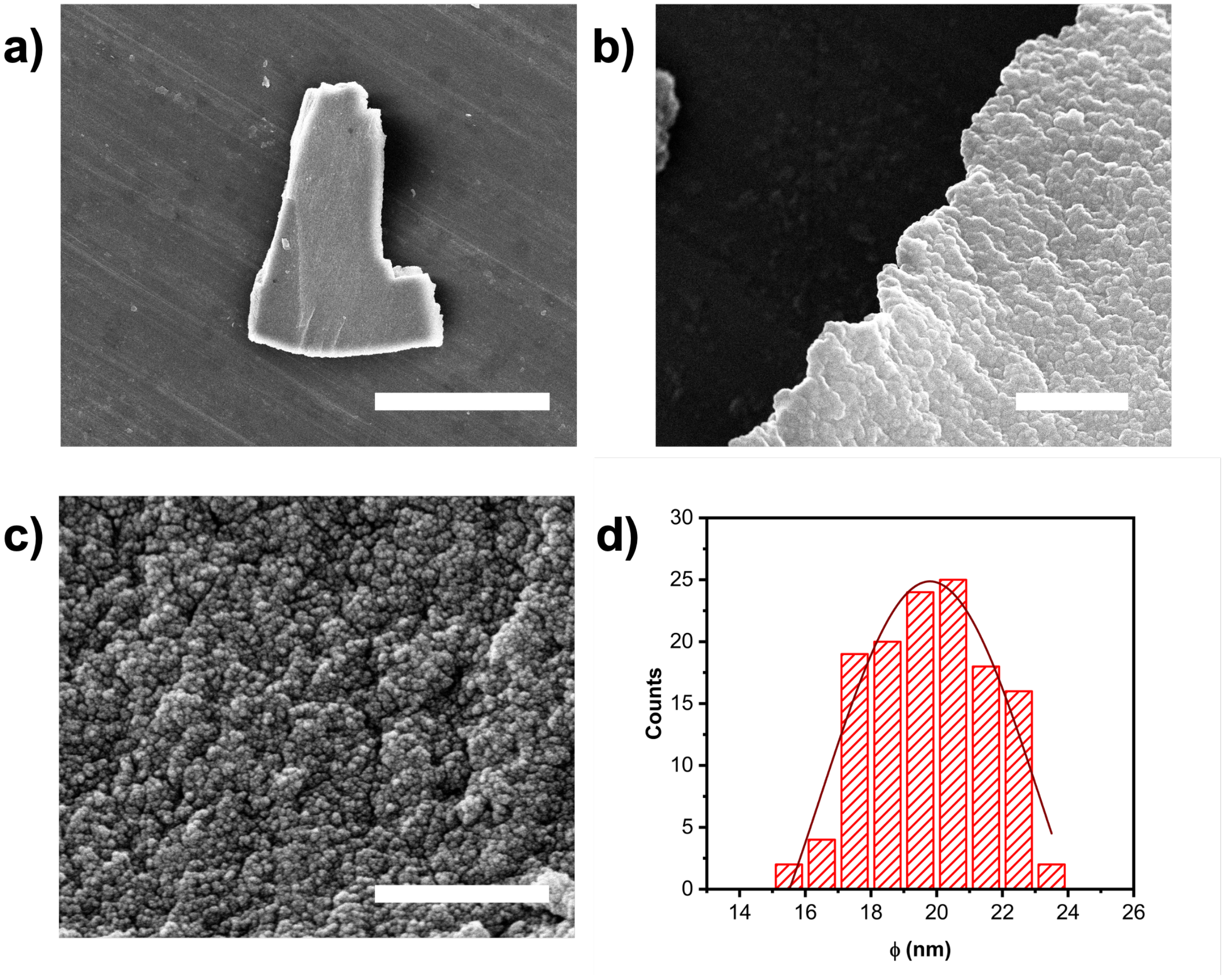

Figure S67 a) FESEM image of monolithic aggregate of RhCu-(COOH)btc-HKUST-1 nanoparticles. (b, c) High magnification images of the surface of the monolithic aggregate showing that it is composed of the close packing of nanoparticles. d) Particle size distribution histogram of RhCu-(COOH)btc-HKUST-1 with a mean particle size of 20 ± 3 nm. Scale bars: 20 μm (a), 1 μm (b), and 500 nm (c).

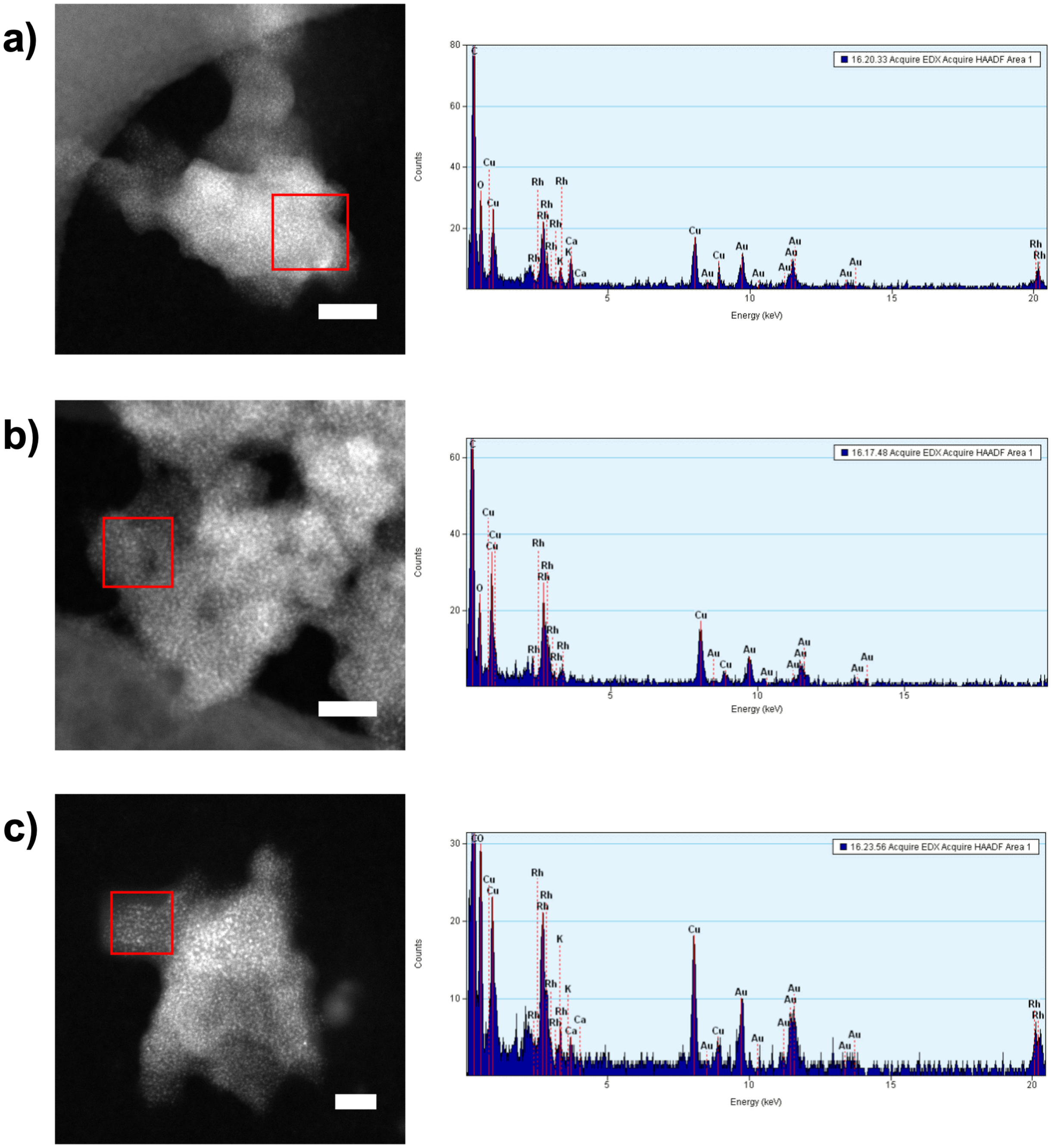

Figure S68 (a-c) Representative HAADF-STEM images (left) and the corresponding EDX spectra (right) collected in single RhCu-(COOH)btc-HKUST-1 nanoparticles (right). Scale bars: 20 nm (a, b, c). Note that Au signals stem from the TEM grid used for these measurements.

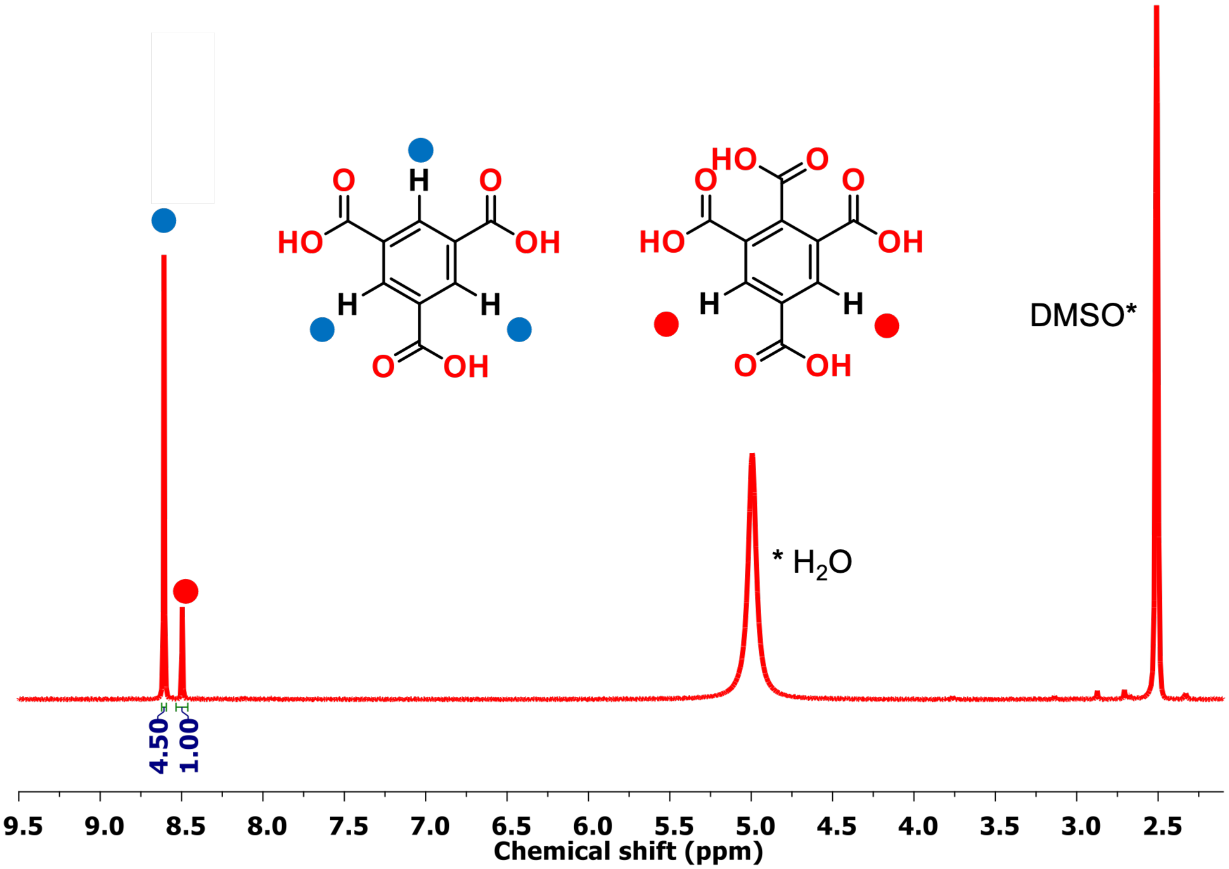

Figure S69 ^{1}H NMR spectra (300 MHz, DMSO-d_6) of fully digested RhCu-(COOH)btc-HKUST-1. Note that the connectivity of RhCu-(COOH)btc-HKUST-1 should define a COOH-RhMOP:(COOH)btc ratio of 1:8. As the complete acid digestion of COOH-RhMOP should release 24 btc linkers, the expected btc:(COOH)btc molar ratio should be 24:8 or 3:1. Moreover, taking into account that each signal of btc integrates three protons (blue dot) and that each signal of (COOH)btc integrates two protons (red dot), the ratio of the signals ascribed to btc and (COOH)btc should be 4.5:1. Therefore, the ^{1}H-NMR spectra of the fully digested RhCu-(COOH)btc-HKUST-1 confirms that the btc:(COOH)btc molar ratio is indeed 3:1.

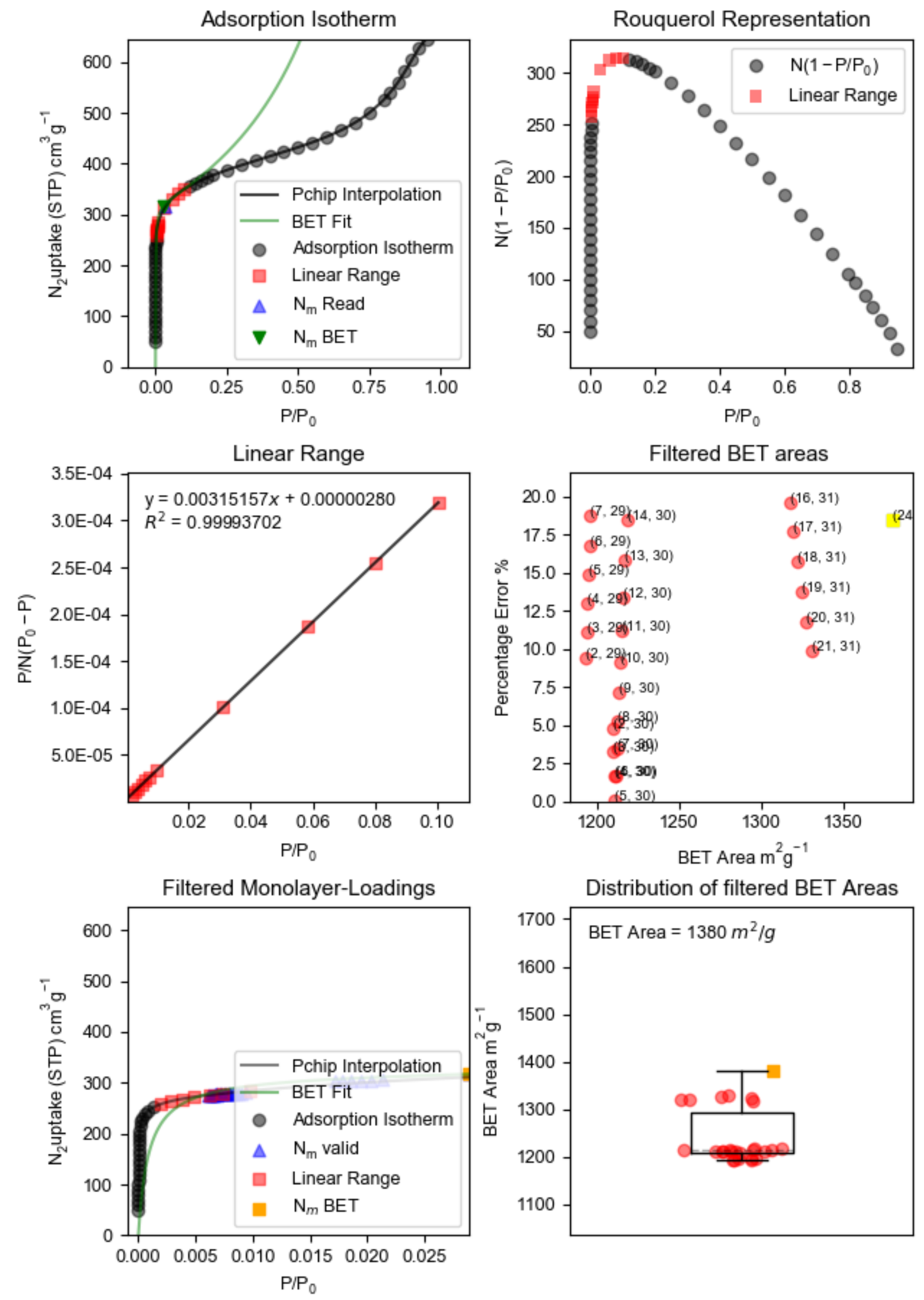

Figure 70 BETSI analysis of RhCu-(COOH)btc-HKUST-1.

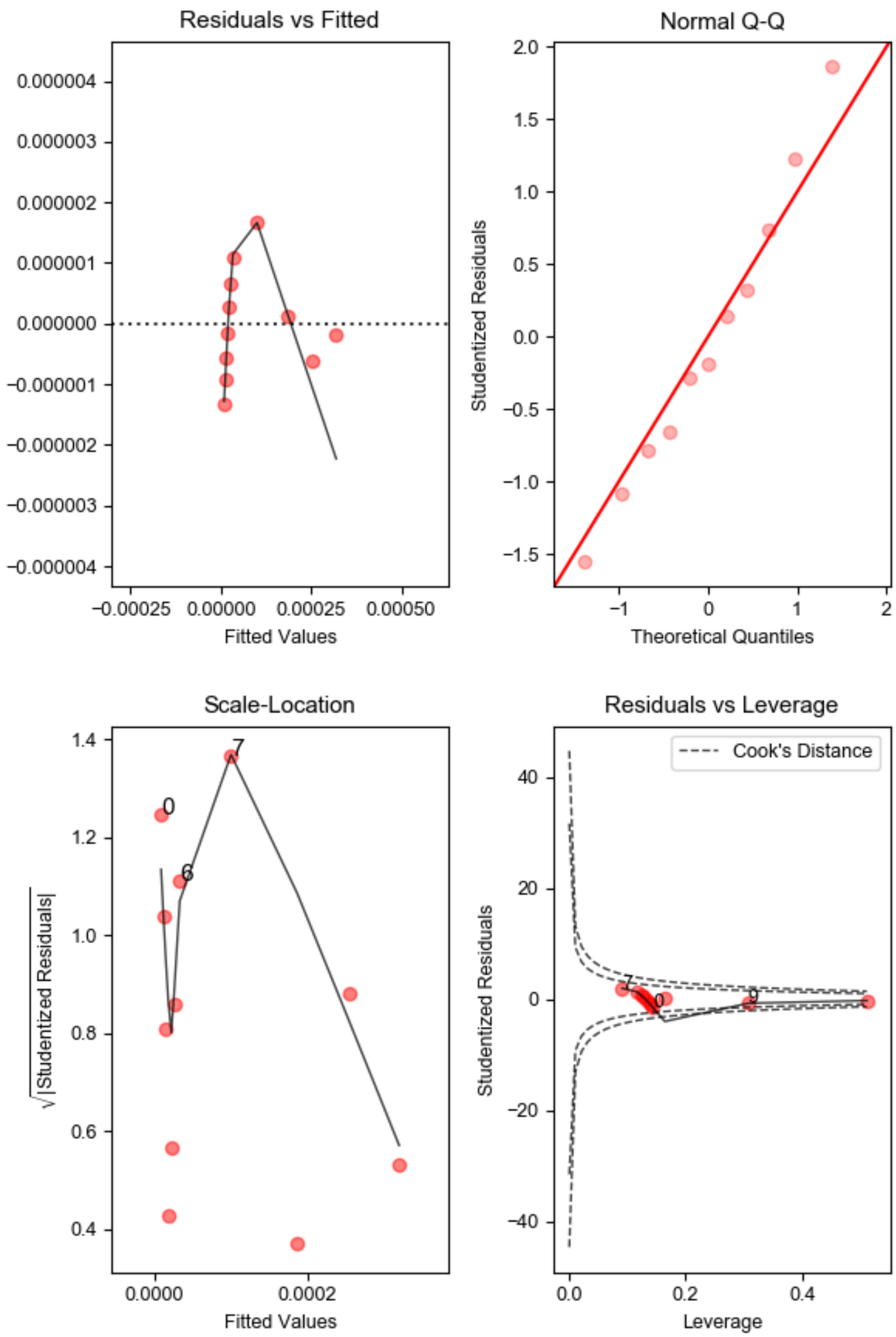

Figure S71 BETSI regression diagnostics of RhCu-(COOH)btc-HKUST-1.

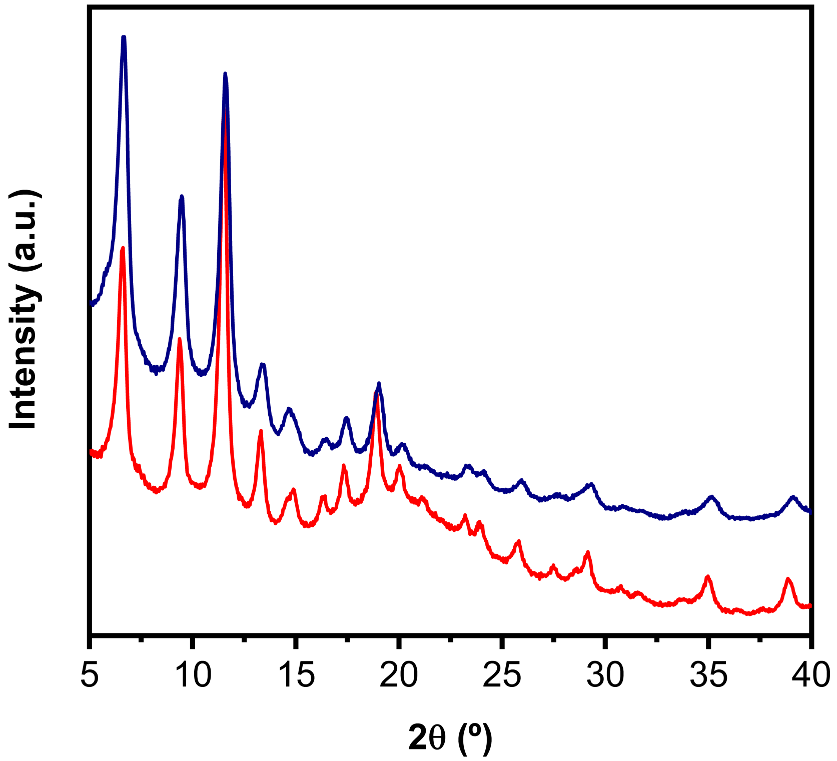

Figure S72 PXRD of the as-made RhCu-(COOH)btc-HKUST-1 (red) and activated RhCu-(COOH)btc-HKUST-1 (blue).

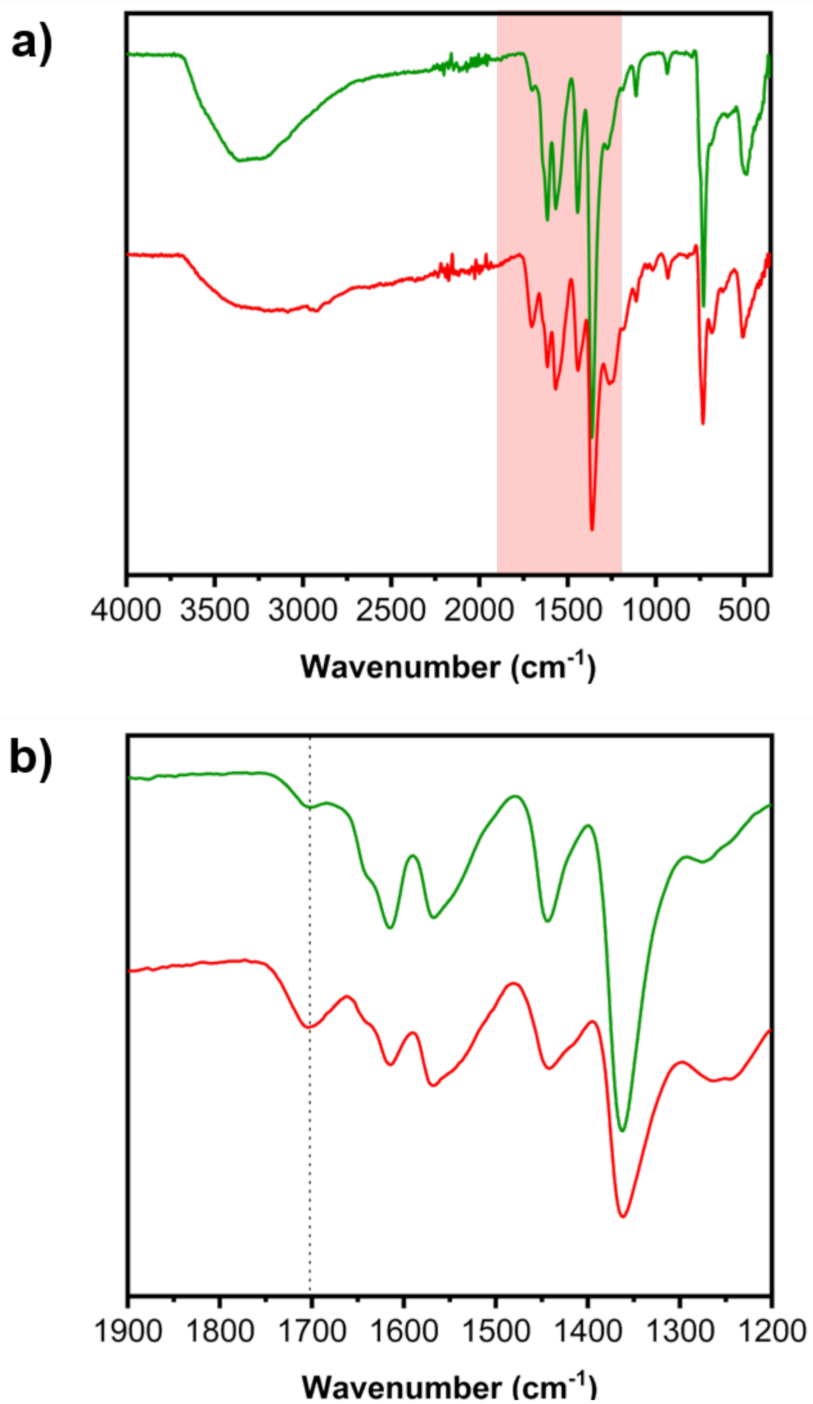

Figure S73 (a) Full FT-IR spectra of RhCu-btc-HKUST-1 (green) and RhCu-(COOH)btc-HKUST-1 (red), and (b) magnified area of the spectra. Note that RhCu-(COOH)btc-HKUST-1 present a more intense peak at 1702 cm^{-1} than RhCu-btc-HKUST-1. This could be ascribed to the C=O stretching band of the un-coordinated carboxylic groups of the functionalized (COOH)btc ligand.

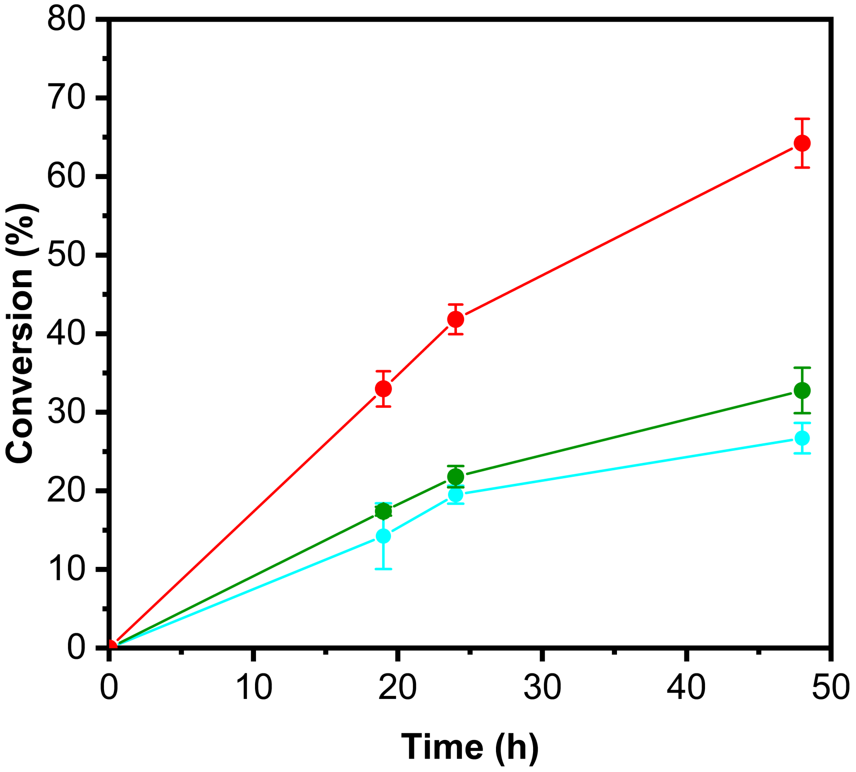

Figure FS74 Conversion of benzaldehyde dimethyl acetal to benzaldehyde as a function of time in the presence of 13 mg of RhCu-(COOH)btc-HKUST-1, 13 mg of RhCu-btc-HKUST-1 (green) and without any catalyst (light blue).

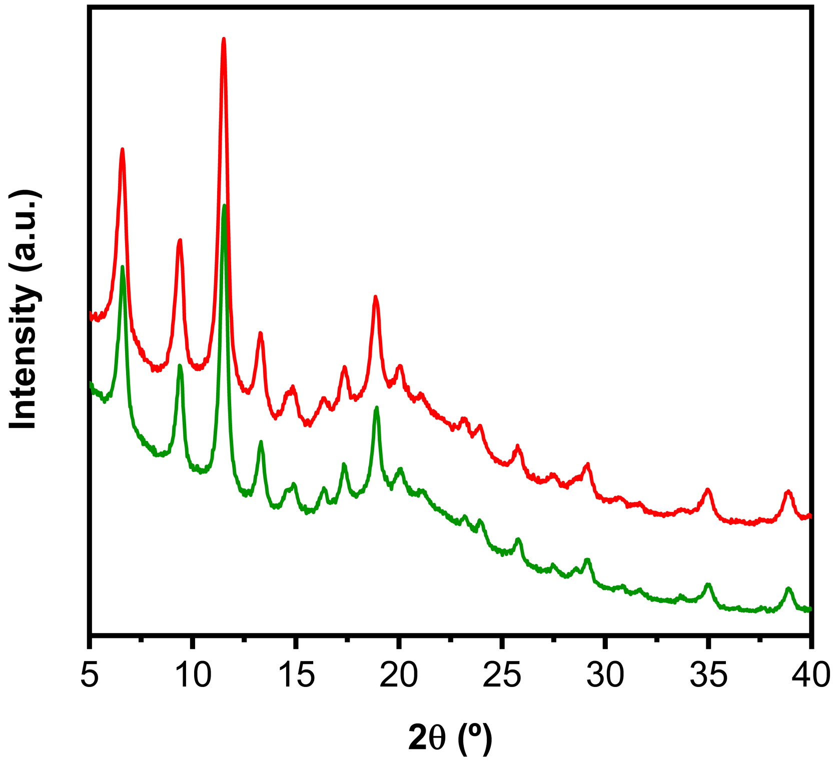

Figure S75 PXRD of RhCu-btc-HKUST (green) and RhCu-(COOH)btc-HKUST-1 (red) after the acid catalysis. Note that both materials maintain the crystallinity after the catalysis reaction.

S9. References.

(1) Osterrieth, J.; Rampersad, J.; Madden, D. G.; Rampal, N.; Skoric, L.; Connolly, B.; Allendorf, M.; Stavila, V.; Snider, J.; Ameloot, R.; Marreiros, J.; Ania, C. O.; Azevedo, D. C. S.; Vilarrasa-García, E.; Santos, B. F.; Bu, X.-H.; Zang, X.; Bunzen, H.; Champness, N.; Griffin, S. L.; Chen, B.; Lin, R.-B.; Coasne, B.; Cohen, S. M.; Moreton, J. C.; Colon, Y. J.; Chen, L.; Clowes, R.; Coudert, F.-X.; Cui, Y.; Hou, B.; D'Alessandro, D.; Doheny, P. W.; Dinca, M.; Sun, C.; Doonan, C.; Huxley, M.; Evans, J. D.; Falcaro, P.; Riccò, R.; Farha, O. K.; Idrees, K. B.; Islamoglu, T.; Feng, P.; Yang, H.; Forgan, R.; Bara, D.; Furukawa, S.; Sanchez, E.; Gascon, J.; Telalovic, S.; Ghosha, S. K.; Mukherjee, S.; Hill, M. R.; Sadiq, M. M.; Horcajada, P.; Salcedo-Abraira, P.; Kaneko, K.; Kukobat, R.; Kenvin, J.; Keskin, S.; Kitagawa, S.; Otake, K.; Lively, R. P.; DeWitt, S. J. A.; Llewellyn, P. L.; Lotsch, B.; Emmerling, S. T.; Pütz, A.; Martí-Gastaldo, C.; Muñoz, N.; Garcia-Martinez, J.; Linares, N.; Maspoch, D.; Suarez, J. A.; Moghadam, P.; Oktavian, R.; Morris, R.; Wheatley, P.; Navarro, J.; Petit, C.; Danaci, D.; Rosseinsky, M.; Katsoulidis, A.; Schroder, M.; Han, X.; Yang, S.; Serre, C.; Mouchaham, G.; Sholl, D.; Thyagarajan, R.; Siderius, D.; Snurr, R. Q.; Goncalves, R. B.; Telfer, S. G.; Lee, S. J.; Ting, V.; Rowlandson, J.; Uemura, T.; Iiyuka, T.; Veen, M. van der; Rega, D.; Vanspeybroeck, V.; Lamaire, A.; Rogge, S.; Walton, K.; Bingel, L. W.; Wuttke, S.; Andreo, J.; Yaghi, O.; Zhang, B.; Yavuz, C.; Nguyen, T.; Zamora, F.; Montoro, C.; Zhou, H.-C.; Angelo, K.; Fairen-Jimenez, D. How Reproducible Are Surface Areas Calculated from the BET Equation? *Adv. Mater*. **2022**, 2201502.

(2) Juanhuix, J.; Gil-Ortiz, F.; Cuní, G.; Colldelram, C.; Nicolás, J.; Lidón, J.; Boter, E.; Ruget, C.; Ferrer, S.; Benach, J. Developments in optics and performance at BL13-XALOC, the macromolecular crystallography beamline at the Alba Synchrotron. *J. Synchrotron Rad.* **2014,** 21, 679-689

(3) Hammersley, A. P. FIT2D: a multi-purpose data reduction, analysis and visualization program. *J. Appl. Cryst.* **2016**, 49, 646-652

(4) Basham, M.; Filik, J.; Wharmby, M. T.; Chang, P. C. Y.; El Kassaby, B.; Gerring, M.; Aishima, J.; Levik, K.; Pulford, B. C. A.; Sikharulidze, I.; Sneddon, D.; Webber, M.; Dhesi, S. S.; Maccherozzi, F.; Svensson, O.; Brockhauser, S.; Náray, G.; Ashtona, A. W. Data analysis workbench (DAWN). *J. Synchrotron Radiat*. **2015**, 22, 853–858.

(5) Juhás, P., Davis, T., Farrow, C.L.; Billinge, S. J. L. PDFgetX3: A rapid and highly automatable program for processing powder diffraction data into total scattering pair distribution functions. *J. Appl. Crystallogr*. **2013**, 46, 560-566.

(6) Yang, X.; Juhas, P.; Farrow, C. L.; Billinge, S. J. L. xPDFsuite: an end-to-end software solution for high throughput pair distribution function transformation, visualization and analysis. arXiv 1402.3163, **2014**.

(7) Albalad, J.; Carné-Sánchez, A.; Grancha, T.; Hernández-López, L.; Maspoch, D. Protection Strategies for Directionally-Controlled Synthesis of Previously Inaccessible Metal–Organic Polyhedra (MOPs): The Cases of Carboxylate- and Amino-Functionalised Rh(II)-MOPs. *Chem. Commun.* **2019**, *55*, 12785–12788.

(8) Invernizzi, C.; Dalvit, C.; Stoeckli-Evans, H.; Neier, R. Synthesis and NMR Spectroscopic Study of the Self-Aggregation of 2-Substituted Benz-ene-1,3,5-Tricarboxamides. *Eur. J. Org. Chem.* **2015**, 23, 5115–5127.

(9) Grancha, T.; Carné-Sánchez, A.; Zarekarizi, F.; Hernández-López, L.; Albalad, J.; Khobotov, A.; Guillerm, V.; Morsali, A.; Juanhuix, J.; Gándara, F.; Imaz, I.; Maspoch, D. Synthesis of Polycarboxylate Rhodium(II) Metal–Organic Polyhedra (MOPs) and Their Use as Building Blocks for Highly Connected Metal–Organic Frameworks (MOFs). *Angew. Chem. Int. Ed.* **2021**, *60*, 5729–5733.

(10) Frisch, M. J.; G.Trucks, G. W.; Schlegel, H. B.; Scuseria, G. E.; Robb, M. A.; Cheeseman, J. R.;Scalmani, G.; Barone, V.; Petersson, G. A.; Nakatsuji, H.; et al. Gaussian 16, Revision A. 03, Gaussian Inc., Wallingford CT, **2016**.
(11) Zhao, Y.; Truhlar, D. G. The M06 suite of density functionals for main group thermochemistry, thermochemical kinetics, noncovalent interactions, excited states, and transition elements: two new functionals and systematic testing of four M06-class functionals and 12 other functionals. *Theor. Chem. Acc.* **2008**, 120, 215-41.
(12) Tomasi, J.; Mennucci, B.; Cammi, R. Quantum Mechanical Continuum Solvation Models *Chem. Rev.* **2005**, 105, 2999 –3094.
(13) Gallardo-Basile, F.-J. Metal Organic Frameworks: An interplay between experimental and theoretical information. Universidad de Sevilla, https://hdl.handle.net/11441/125961
(14) W.-T. Chan, R. Fournier. Binding of ammonia to small copper and silver clusters. *Chem. Phy. Lett.* **1999**, 315, 257-265.
(15) Humphrey, W.; Dalke, A.; Schulten, K. VMD - Visual Molecular Dynamics. J. Molec. Graphics, **1996**, 14, 33-38.
(16) Gopiraman, M.; Deng, D.; Ganesh Babu, S.; Hayashi, T.; Karvembu, R.; Kim, I. S. Sustainable and Versatile CuO/GNS Nanocatalyst for Highly Efficient Base Free Coupling Reactions. *ACS Sustain. Chem. Eng.* **2015**, *3*, 2478–2488.
(17) Shi, Y.; Hu, X.; Zhu, B.; Wang, S.; Zhang, S.; Huang, W. Synthesis and Characterization of TiO_2 Nanotube Supported Rh-Nanoparticle Catalysts for Regioselective Hydroformylation of Vinyl Acetate. *RSC Adv.* **2014**, *4*, 62215–62222.
(18) Kirchon, A.; Day, G. S.; Fang, Y.; Banerjee, S.; Ozdemir, O. K.; Zhou, H.-C. Suspension Processing of Microporous Metal-Organic Frameworks: A Scalable Route to High-Quality Adsorbents. *iScience* **2018**, *5*, 30–37.
(19) Chui, S. S.-Y.; Lo, S. M.-F.; Charmant, J. P. H.; Orpen, A. G.; Williams, I. D. A Chemically Functionalizable Nanoporous Material $[Cu_3(TMA)_2(H2O)_3]n$. *Science* **1999**, *283*, 1148–1150.
(20) Majano, G.; Martin, O.; Hammes, M.; Smeets, S.; Baerlocher, C.; Pérez-Ramírez, J. Solvent-Mediated Reconstruction of the Metal–Organic Framework HKUST-1 ($Cu_3(BTC)_2$). *Adv. Funct. Mater.* **2014**, *24*, 3855–3865.
(21) Todaro, M.; Buscarino, G.; Sciortino, L.; Alessi, A.; Messina, F.; Taddei, M.; Ranocchiari, M.; Cannas, M.; Gelardi, F. M. Decomposition Process of Carboxylate MOF HKUST-1 Unveiled at the Atomic Scale Level. *J. Phys. Chem. C.* **2016**, *120*, 12879–12889.
(22) Álvarez, J. R.; Sánchez-González, E.; Pérez, E.; Schneider-Revueltas, E.; Martínez, A.; Tejeda-Cruz, A.; Islas-Jácome, A.; González-Zamora, E.; Ibarra, I. A. Structure Stability of HKUST-1 towards Water and Ethanol and Their Effect on Its CO2 Capture Properties. *Dalton Trans.* **2017**, *46*, 9192–9200.
(23) Lal, G.; Gelfand, B. S.; Lin, J.-B.; Banerjee, A.; Trudel, S.; Shimizu, G. K. H. Three Sequential Hydrolysis Products of the Ubiquitous Cu_{24} Isophthalate Metal−Organic Polyhedra. *Inorg Chem.* **2019,** 58, 9874-9881.
(24) Hernández-López, L.; Martínez-Esaín, J.; Carné-Sánchez, A.; Grancha, T.; Faraudo, J.; Maspoch, D. Steric Hindrance in Metal Coordination Drives the Separation of Pyridine Regioisomers Using Rhodium(II)-Based Metal–Organic Polyhedra. *Angew. Chem. Int. Ed.* **2021,** 60, 11406-11413.
